# Comparative genomics and transcriptomics of *Chrysolophus* provide insights into the evolution of complex plumage coloration

**DOI:** 10.1093/gigascience/giy113

**Published:** 2018-09-06

**Authors:** Guangqi Gao, Meng Xu, Chunling Bai, Yulan Yang, Guangpeng Li, Junyang Xu, Zhuying Wei, Jiumeng Min, Guanghua Su, Xianqiang Zhou, Jun Guo, Yu Hao, Guiping Zhang, Xukui Yang, Xiaomin Xu, Randall B Widelitz, Cheng-Ming Chuong, Chi Zhang, Jun Yin, Yongchun Zuo

**Affiliations:** 1 The State key Laboratory of Reproductive Regulation and Breeding of Grassland Livestock, Inner Mongolia University, No.235, University West Road, Saihan District,Hohhot, Inner Mongolia, 010021, China; 2 College of Life Science, Inner Mongolia University, Hohhot, 010070, China; 3 BGI Genomics, Co., Ltd. Buiding No.7, BGI Park, No.21 Hongan 3rd Street, Yantian District, Shenzhen, 518083, China; 4 College of Life Science, Inner Mongolia Agricultural University, No.306, Zhaowuda Road, Saihan District, Hohhot, Inner Mongolia, 010018; 5 Department of Pathology, Keck School of Medicine, Universit of Southern California, 2011 Zonal Avenue, HMR315B, Los Angeles, CA90033, USA

**Keywords:** genome, transcriptome, *Chrysolophus*, plumage, coloration

## Abstract

**Background:**

As one of the most recognizable characteristics in birds, plumage color has a high impact on understanding the evolution and mechanisms of coloration. Feather and skin are ideal tissues to explore the genomics and complexity of color patterns in vertebrates. Two species of the genus *Chrysolophus*, golden pheasant (*Chrysolophus pictus*) and Lady Amherst's pheasant (*Chrysolophus amherstiae*), exhibit brilliant colors in their plumage, but with extreme phenotypic differences, making these two species great models to investigate plumage coloration mechanisms in birds.

**Results:**

We sequenced and assembled a genome of golden pheasant with high coverage and annotated 15,552 protein-coding genes. The genome of Lady Amherst's pheasant is sequenced with low coverage. Based on the feather pigment identification, a series of genomic and transcriptomic comparisons were conducted to investigate the complex features of plumage coloration. By identifying the lineage-specific sequence variations in *Chrysolophus* and golden pheasant against different backgrounds, we found that four melanogenesis biosynthesis genes and some lipid-related genes might be candidate genomic factors for the evolution of melanin and carotenoid pigmentation, respectively. In addition, a study among 47 birds showed some candidate genes related to carotenoid coloration in a broad range of birds. The transcriptome data further reveal important regulators of the two colorations, particularly one splicing transcript of the microphthalmia-associated transcription factor gene for pheomelanin synthesis.

**Conclusions:**

Analysis of the golden pheasant and its sister pheasant genomes, as well as comparison with other avian genomes, are helpful to reveal the underlying regulation of their plumage coloration. The present study provides important genomic information and insights for further studies of avian plumage evolution and diversity.

## Background

The plumage colors of birds serve functions in crypsis, social signaling, and mate choice [1]. Due to the diversity of colors and ease of observation, plumage provides an ideal model to explore the formation and genomic evolution of coloration patterns in animals. Studies on birds and mammals suggest that the integument colors are regulated by several mechanisms. Melanin, which is produced by neural crest cell-derived melanocytes, is a major contributor to pigmentation in avian feathers and mammalian hairs [2]. Black and brown feathers are derived from the deposition of eumelanin, whereas reddish and light-yellow feathers are due to pheomelanin. Carotenoids are chemicals for vitamin synthesis and act as antioxidants for the immune system [2]. Some birds can use dietary-derived carotenoids, such as lutein, zeaxanthin, β-cryptoxanthin, and β-carotene, to produce yellow, orange, and red in their feathers [3]. Red colors may also come from other rare pigments, such as porphyrins in black-shouldered kites [4], psittacofulvins in parrots [5], iron oxide in *Gypaetus barbatus*, and turacin in *Tauraco macrorhynchus* [6]. In addition, feather coloration may also be a result of specific structures that combine with noniridescent colors and iridescent metal lusters [2].

Feather complex coloration is likely coordinated through multiple genes that regulate diverse mechanisms. The melanogenesis biosynthetic pathway has been elucidated [7, 8], and previous studies have revealed the DNA polymorphisms of several genes that lead to variations in melanin-based coloration [9]. However, some details regulating the switch of eu-/pheomelanin remain unresolved [10]. Some candidate genes for carotenoid-related functions in mammals and invertebrates have been documented, and their homologous genes may also be present in birds [11]. However, the production metabolism of carotenoid pigments has not been well characterized. Additionally, the nanostructural colors of feathers are related to keratinization and affected by keratin genes [12, 13]. In birds, α- and β-keratin families include nearly 200 members [14] whose functions should be investigated. Genome information could provide new perspectives to study the mechanisms of bird coloration. In 2014, the most extensive comparative analysis of avian species at the genome level to date was published, revealing two genes with a negative correlation between color discriminability and *dN/dS* across birds [15, 16]. However, this work included only 15 genes without distinguishing melanin, carotenoid, or other pigments. Thus, further studies are necessary to investigate the candidate molecular mechanisms of avian plumage coloration.

**Figure 1:**
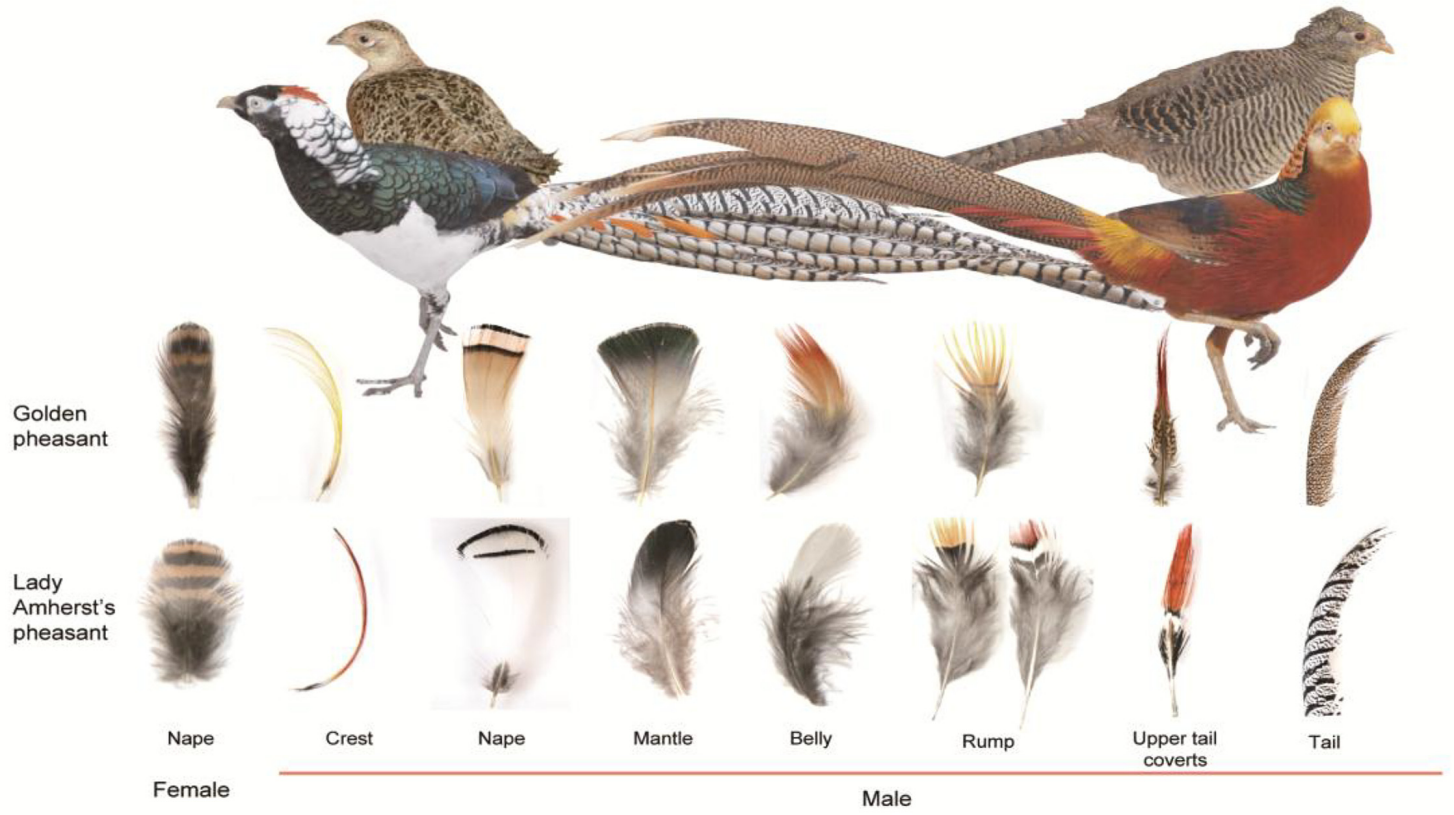
Profile of golden pheasant (upper right) and Lady Amherst's pheasant (upper left) and their feathers from different body parts (lower part). Both male species (near) are more colorful than females (far). The female feathers are represented by the napes.

In the present study, we focused on the plumage coloration issues of golden pheasant (*Chrysolophus pictus*) at the genome and transcriptome levels, together with its sister species, the Lady Amherst's pheasant (*Chrysolophus amherstiae*). These species are two important organisms for studies of plumage coloration because of their phenotypic differences and close relationship. These two species can even crossbreed to produce fertile offspring under human feeding conditions. In adult male golden pheasant, both the crest and rump feathers are golden-yellow in color, the belly and upper tail coverts are dark red, the nape feathers are light orange with two black stripes, the mantle is iridescent green, and the tail is black spotted with cinnamon (Fig. 1, Supplementary Fig. S1). The golden pheasant is a colorful avian species with distinct brilliant feather colors in adult males, which can be observed with obvious characteristics of melanin and carotenoid pigments. By comparison, adult male Lady Amherst's pheasants have red and yellow feathers exclusively distributed over small parts of the body, including the crest, rump, and upper tail coverts, while most of the other body parts are white or black (Fig. 1, Supplementary Fig. S1). Carotenoids were present in the yellow back feathers of golden pheasant, but it was unclear whether they were present in Lady Amherst's pheasant [17]. In the present study, we sequenced the genome and transcriptome of these two pheasants and identified the melanin and carotenoid pigments in plumages of the two pheasant species by using high-performance liquid chromatography (HPLC) and Raman spectroscopy (RS) methods. Then, we conducted a comprehensive comparative analysis with 51 other sequenced avian references [15, 18, 19] at a suitable level to investigate the evolution of the plumage coloring of golden pheasant or *Chrysolophus*.

## Results and Discussion

### Genome assembly and annotation

The genomic DNA of golden pheasant was extracted by using blood genomic DNA from a male adult from Foping National Nature Reserve in Shaanxi and fed in Jilin, China. A series of paired-end libraries with different insert sizes were constructed and sequenced by using the Illumina Hiseq 2000 platform (Supplementary Table S1). The *de novo* assembly size was 1.029 Gb, with a contig N50 size of 34.4 kb and a scaffold N50 size of 1.55 Mb (Table 1). Assembly quality was assessed by aligning the total small insert size reads (170 ∼ 800 bp) to the assembly. These reads covered 99.92% of the genome, and 99.17% of the alignment could be mapped by more than 10 reads (Supplementary Table S2 and Fig. S2). In addition, the assembly covered more than 95.71% of the transcriptome-assembled transcripts (102,426 of 107,012; Supplementary Table S3), indicating the high quality of the golden pheasant assembly.

**Table 1:** Statistics of assembly and annotation for the golden pheasant genome.

**Genome characteristics**	**Data**
Assembly features	
Estimate of genome size	1032,423,981 bp
Total size of assembled scaffolds	1028,603,357 bp
Scaffold N50	1547,393 bp
Longest scaffold	18,323,375 bp
Total size of assembled contigs	1003,285,807 bp
Contig N50	34,356 bp
Longest contig	257,270 bp
GC content (excluding Ns)	40.80%
Annotation features	
Number of gene models	15,552
Mean coding sequence length	1705.27 bp
Mean number of exons per gene	9.94
Mean exon length	171.62 bp
Mean intron length	2397.68 bp
Total size of REs	112,429,773 bp
REs share in genome	10.93%

*RE, repetitive elements.

**Figure 2:**
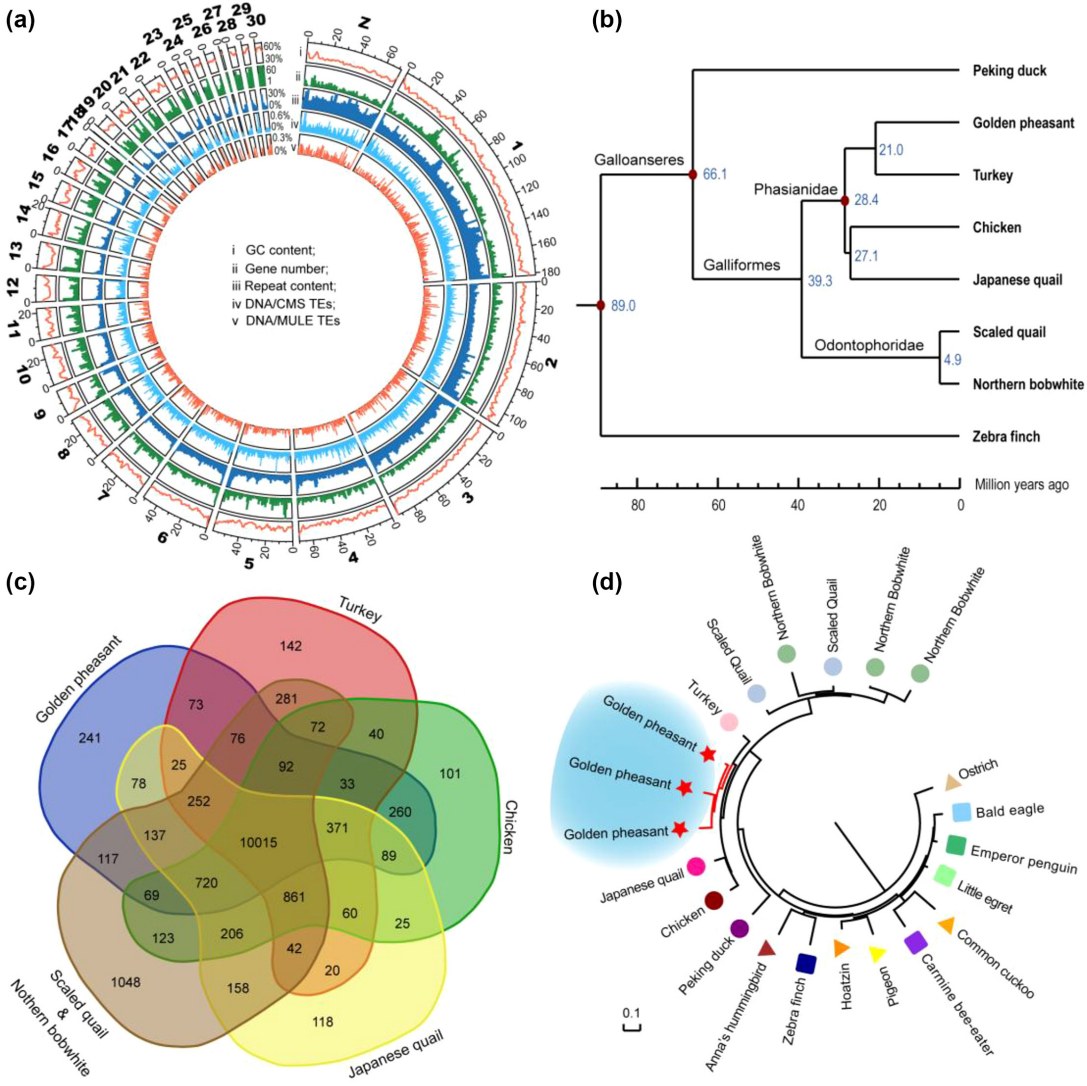
Comparative genomic analyses among the golden pheasant and other avian species. **(a)** Global view of the golden pheasant genome using the pseudochromosomes. **(b)** The maximum likelihood phylogenetic relationships of the golden pheasant in Galliformes. The tree was constructed based on 996,755 bp 4-fold degenerate sites, from 6,538 single-copy orthologous genes among six sequenced Galliformes genomes (golden pheasant, chicken, turkey, Japanese quail, northern bobwhite, and scaled quail), the sequenced Anseriformes (duck), and the zebra finch (as outgroup). **(c)** Venn diagram of the shared orthologous gene families among the Galliformes species. **(d)** The maximum likelihood phylogeny tree of CYP2D genes in 17 avian species. The background species are selected based on Galliformes species and Jarvis's phylogeny for the 48 avian genomes [16], of which 11 birds with high quality of genome build from 10 different clades are selected in this analysis.

To obtain a global view of potential specific elements in golden pheasant, 93.9% of the assembly was linked to pseudochromosomes by using turkey chromosomes as a reference (Fig. 2a). The genomic DNA from a male Lady Amherst's pheasant was sequenced with relatively low coverage (approximately 43×). We identified 7.26 million single-nucleotide polymorphisms (SNPs) and 0.45 million insertions and deletions (indels) (1–5 bp per indel, total 0.83 Mb length) (Supplementary Table S4) in Lady Amherst's pheasant by using the assembly of golden pheasant as a reference, indicating that the divergence between these two pheasants was approximately 0.84%. Moreover, the golden pheasant genome was used as a reference to align the transcriptome sequences from these two species. The average mapping rates of golden pheasant and Lady Amherst's pheasant are 85.82% and 81.83%, respectively. These results imply a close relationship between these two species.

Combining the homology-based and transcriptome-assisted methods, 15,552 protein-coding genes were identified in the assembly of golden pheasant, of which 98.69% of the genes were homologous to public databases (SwissProt, Nr, and Kyoto Encyclopedia of Genes and Genomes [KEGG]) (Supplementary Table S5), and 89.43% of the genes were supported by transcriptome sequences (reads per kilobase million [RPKM] >1 in at least one sample). Moreover, repetitive elements (REs) comprised approximately 10.93% of the golden pheasant genome, with the chicken repeat 1 elements being the most abundant class (83.14% of REs; 0.093 Gb), which was similar to that for chicken (Supplementary Table S6). The expanded satellite DNAs in the golden pheasant genome were 5.5- and 18.2-fold that of the chicken and zebra finch genomes, respectively (Supplementary Fig. S3 and Table S7). There were no lineage-specific REs identified in golden pheasant, but a similar evolutionary trend of a class of DNA transposon,usually used with DNA as DNA/CMC and DNA/MULE (DNA/Mus like element,usually used with DNA as DNA/MULE) transposable elements (TEs) were found between the golden pheasant and turkey (Supplementary Table S7). Increasing evidence has suggested that TEs might play a role as candidate gene expression regulators, especially in the modulation of abutting gene expression [20-22]. Thus, genes within 2 kb up- and downstream of these TEs were examined. The flanking genes of the satellite DNAs, CMC, and MULE could be enriched in sodium-potassium exchange ATPase activity Gene Ontology (GO: 0 005391, adjusted *P* value = 0.02295), cell development (GO: 00 48468, adjust *P* value = 1.93E-08), and kidney development (GO: 0 001822, adjusted *P* value = 0.00128), respectively (Supplementary Fig. S4). Functional enrichment showed that the specific or expanded REs may be involved in the adaptive evolution of golden pheasant or turkey.

### Evolution analysis within Galliformes

The phylogenetic placement is a critical background for many comparative genomic analyses. To assess the phylogenetic position of the golden pheasant in Galliformes, a phylogenetic tree was constructed with five other sequenced Galliformes (chicken [23], turkey [24], Japanese quail [18], northern bobwhite,[19] and scaled quail [19]); the sequenced Anseriformes (duck [25]), which is closest to Galliformes; and a model species (zebra finch [26]) as an outgroup. The phylogenetic analysis of 48 birds concluded that protein-coding genes might reflect life history traits more than phylogeny topology would [16]. Therefore, we constructed the phylogeny tree using 996,755 4-fold degenerate (4D) sites (from 6,538 one-to-one orthologous genes) that are sites that do not change the amino acid and are typically considered to be less subject to selective pressure. The result showed that the golden pheasant is taxonomically closer to turkey than to chicken (Fig. 2b, Supplementary Fig. S5). The relationship was consistent with the above REs analysis that golden pheasant and turkey had similar divergence distribution (Supplementary Fig. S3) and shared some common specific REs, which belong to noncoding regions (Supplementary Table S7). This phylogeny was also uncontroversial with a previous study that was based on six nuclear intron sequences and two mitochondrial regions [27]. Furthermore, the divergence time of the golden pheasant and turkey was estimated approximately 13 million years ago (Mya) by using MCMCTree (Fig. 2b).

Sequence divergences and/or gene duplications have been proposed as important mechanisms in the course of evolution [28]. Identifying these variations may provide clues for the next investigations. Positive Darwinian selection is a universal strategy to identify candidates of adaptive evolution at the DNA sequence level. For the 6,538 one-to-one orthologous genes in eight birds, 676 positive selected genes were identified in golden pheasant by using branch site model (Supplementary Tables S8 and S9). For the multicopy gene families, 241 lineage-specific gene families were identified in golden pheasant (Fig. 2c) by hierarchical clustering. Additionally, we identified 132 expanded and 18 contracted gene families through a maximum likelihood framework (Supplementary Tables S10 and S11). It is noteworthy that cytochrome P450 family 2 subfamily D member 6 (*CYP*2*D*6) was duplicated to three copies in the golden pheasant genome (Fig. 2d, Supplementary Fig. S6), whereas only one copy was found in 48 other birds [16]. Although multiple copies of this gene are present in northern bobwhite and scaled quail, it is likely that independent duplication events occurred in the two Odontophoridae species and golden pheasant, respectively, based on our phylogeny (Fig. 2d). The CYP enzymes were considered good candidates for carotenoid ketolases [29]. Recently, a comparative analysis among 65 bird genomes revealed the *CYP*2*J*19 gene, which belonged to the same clan as that of *CYP*2*D* and was a carotenoid ketolase functional in synthesizing red carotenoids from yellow carotenoids [30], and two other population studies revealed the *CYP*2*J*19 was associated with red carotenoid-based coloration phenotypes in zebra finches and canaries [29, 31]. Compared with other non-carotenoid Galliformes, copy number and protein sequence of *CYP*2*J*19 in golden pheasant are conserved (the sequence is more similar to that of the turkey). However, the expression of *CYP*2*J*19 in the orange nape of golden pheasant was significantly higher than other colored feathers. It could be suggested that *CYP*2*J*19 might influence coloration at the transcriptional level in *Chrysolophus*. Additionally, *CYP*2*D*6 had the maximum allelic polymorphism among the CYP family in human [32] and was responsible for approximately 25% of the metabolism of known drugs [33], indicating the wide range of functions of the *CYP*2*D* gene. The expanded *CYP*2*D*6 genes in golden pheasant may function in metabolism or biotransformation of some foreign chemicals and could be a candidate for carotenoid deposition in its feathers.

### Lineage-specific variations and alternative splicing of melanin genes in *Chrysolophus*

Melanin is the most common and widespread pigment in avian feathers and yields black, gray, brown, rufous, and buff shades and patterns [2]. Both *Chrysolophus* species possessed darker eumelanic and brighter pheomelanic colors in their integument plumage, particularly the most impressive bright red and yellow feathers in male individuals (Fig. 1). A previous investigation concluded that human hairs with six different colors, varying from black to brown to red, all contained both eumelanin and pheomelanin and that their proportions determined the visual colors. The eumelanin content and proportions were the highest in black hairs, while red hairs contained comparable levels of eumelanin and pheomelanin [34]. The present HPLC results also showed that feathers with different colors from golden pheasant and Lady Amherst's pheasant varied according to the ratio of eu-/pheomelanin (Supplementary Fig. S7). This finding could indicate that the clear feather colors of the two pheasant species might result from the relatively extreme mixture ratio of eu-/pheomelanin. Based on this information, we focused on the genetic regulations of the eu-/pheomelanin switch in *Chrysolophus* birds from both genomic and transcriptomic perspectives.

**Figure 3:**
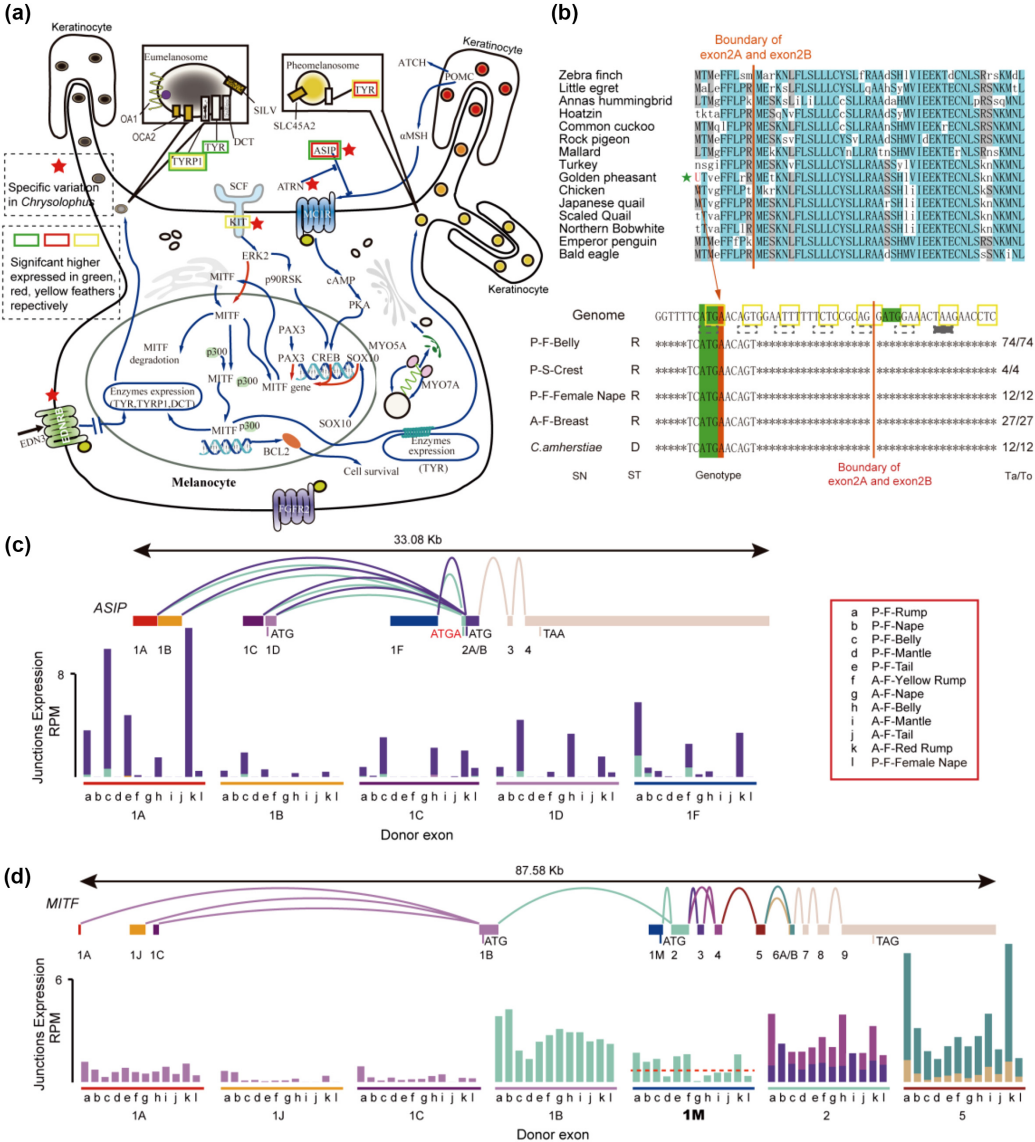
The variation and alternative splicing of some regulator genes in the eu-/pheomelanin synthesis metabolism. **(a)** The pathway of eu-/pheomelanin synthesis metabolism. The lineage-specific varied genes in *Chrysolophus* are marked by a red star. The significant higher expressed genes in feathers with green, red, and yellow color are marked by the colorful rectangle, respectively; all use the white feathers (A-F-Nape and A-F-Belly) as control. **(b)** The single-nucleotide insertion in the ASIP gene of the *Chrysolophus*. A base of adenine inserts after the initiation codon of the open reading frame at exon 2A. This insertion was verified in another five *Chrysolophus* individuals (lower part). SN, sample name; ST, sequencing type; R, RNA sequencing; D, DNA sequencing; Ta/To, the number of reads support the shown genotype/the number of total mapped reads. **(c)** The RNA alternative splicing of the *ASIP* gene. The upper section shows the alternative splicing models of *ASIP*. Rectangles represent exons, and curves represent junctions between the exons. The size scale ratio between exons and introns is 1:10. The lower section is the expression the histogram of the junctions. Reads per million mapped reads was used to normalize expression levels. The color of the column matches the accepter exon color. The color of the footstone matches the donor exon color. **(d)** The RNA alternative splicing of *MITF* gene. Descriptions are that same as in **(c)**. The description of sample name: P, golden pheasant; A, Lady Amherst's pheasant; F, feather; S, skin.

We identified the lineage-specific varied genes in *Chrysolophus* by making comparisons with the five other Galliformes species and 11 additional birds with high quality of genome build that belong to 11 different clades in the phylogeny tree of the 48 birds [16]. Four melanogenesis-associated genes have specific mutated sites in *Chrysolophus* species, including attractin (ATRN), endothelin receptor B (EDNRB), KIT proto-oncogene tyrosine-protein kinase (KIT), and agouti signaling protein (ASIP) (Fig. 3a). ATRN has at least eight sites under positive selection, with >1 (BEB test [35], *P* > 0.98), which could prevent the formation of the “Kelch repeat type 1” domain (PF01344) based on the InterProScan annotation [36] (Supplementary Fig. S8). EDNRB has a three-amino acid deletion in the “G protein-coupled receptor, rhodopsin-like” domain (PF00001; Supplementary Fig. S9), and KIT has a two-amino acid deletion in the C-terminal region, which is conserved in other birds and even in green anole (Supplementary Fig. S10). In the *ASIP* gene, a single nucleotide is inserted after the initiation codon at exon 2A, which may impact 50% of ASIP isoforms by disabling this initiation codon or causing a frameshift, resulting in a premature transcription termination at the 13th cordon (Fig. 3b). Melanogenesis is under multiple levels of complex regulation, mainly through the transcriptional and post-transcriptional regulation of the *MITF* gene, which can stimulate the transcription of genes that function in producing melanin [37-40]. The classic transcriptional regulator of *MITF* is the melanocortin-1 receptor (MC1R) with its ligands, alpha-melanocyte-stimulating hormone (α-MSH) and ASIP. ASIP can competitively antagonize α-MSH to bind MC1R, and ATRN is an obligatory accessory receptor for ASIP that enhances ASIP-Mc1R binding [37]. From another aspect, KIT can mediate the phosphorylation of MITF protein at Ser73 through the mitogen-activated protein kinase (MAPK) pathway and trigger short-lived MITF activation as well as ubiquitin-dependent proteolysis [38, 39]. Moreover, EDNRB stimulation not only activates MITF expression but also elicits MAPK-mediated MITF phosphorylation [40]. As located in the upstream of the melanogenesis pathway, variations of these four genes may amplify the biosynthesis or switches of eumelanin and pheomelanin through a signaling cascade [38, 39], resulting in a more extreme mixture ratio of eu-/pheomelanin in *Chrysolophus*.

Gene variations can alter plumage color traits among different birds; however, the diversity of colors and patterning present in one individual may be due to gene expression or alternative splicing [41]. Two promoters of the *ASIP* gene, the proximal hair cycle-specific promoter and the distal ventral-specific promoter, have been identified in mice and rabbits [42, 43]. Recent reports identified three conserved classes of ASIP mRNA variants that are specifically expressed in the dorsal and ventral feather follicles of chickens [44, 45]. We sequenced the RNA of feather follicles from different body parts in two pheasants and identified at least 10 *ASIP* mRNA isoforms generated by alternative splicing (Supplementary Table S12), in which *ASIP*-1A isoforms are highly expressed in red-pheomelanin feathers, while *ASIP*-1F isoforms are abundant in yellow-pheomelanin feathers (Fig. 3c). MITF, another central regulatory element of the melanogenesis pathway, regulates at least 11 melanogenesis genes directly or indirectly through feedback loops [46] and exhibits a complex alternative splicing pattern in *Chrysolophus* feather follicles. The *MITF* consists of at least 13 exons and two opening reading frames (ORFs) that are translated from exon-1B and exon-1M (Fig. 3d; Supplementary Table S13). We demonstrate herein that *MITF-M* isoforms are preferentially expressed in pheomelanin-containing feathers (fold change = 3.80, adjusted *P* value = 1.35E-11; Fig. 3d). *MITF-M* has been thought to be specifically expressed by melanocytes, but its expression has been identified in the retinal pigment epithelium [47]. This result indicates that the *MITF*-1M isoform may be a key factor in regulation of pheomelanin synthesis in the feather follicles of *Chrysolophus*.

### Carotenoid utilization in *Chrysolophus* plumage

**Figure 4:**
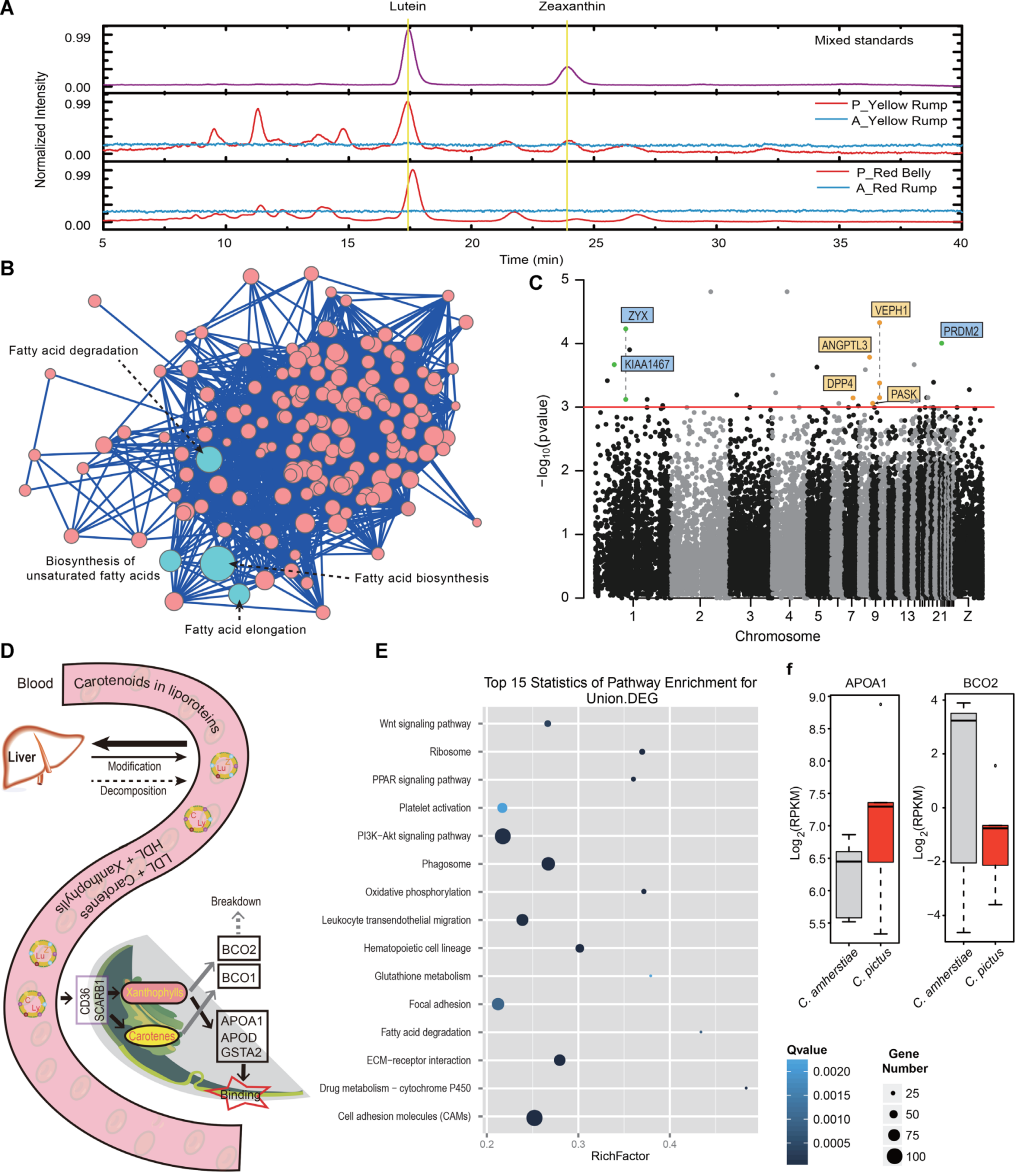
The comparative analysis and RNA expression of the carotenoid accumulation in feather. **(a)** HPLC analysis of lutein and zeaxanthin in *Chrysolophus* red and yellow feathers. **(b)** The KEGG pathway annotation of the genes that is lineage specific in golden pheasant but the same in 40 other nonfeather-carotenoid birds. The scoring standard of each pathway is described in the method. **(c)** The orthologous genes wide association study to the carotenoids accumulation. The coordinates are based on chicken chromosomes. Dashed lines indicate the connected sites belonging to the same gene. The green spots are genes that also contain lineage-specific varied sites in golden pheasant. The orange spots are lipid-related genes. **(d)** The theoretical process of carotenoids transportation and deposition. **(e)** The KEGG pathway enrichment of the union differentially expressed genes (DEGs) between feather follicles of the two pheasants. The RichFactor = the number of DEGs in this pathway/the number of gene sets in this pathway. More details are described in Additional File 3, note 4.2. **(f)** The expression of the *APOA*1 and *BCO*2 genes in the two pheasants.

Carotenoids, a class of organic fat-soluble compound, are synthesized by plants, bacteria, and fungi and utilized by animals through their diets [48]. Depending on the chemical structure, these pigments typically appear yellow, orange, or red in avian plumage [2]. In the present study, both pheasant species have yellow to red plumage, but carotenoids were only found in the golden pheasant. RS [49] showed carotenoid bands in golden pheasant feathers but not in Lady Amherst's pheasant feathers (Supplementary Fig. S11a and S11b). Further identification by HPLC revealed that these carotenoids included lutein and zeaxanthin (Fig. 4a, Supplementary Fig. S12, Supplementary Table S14).

The two other sequenced Galliformes, chicken and turkey, also do not accumulate carotenoids in feathers. It is likely that golden pheasant acquired this new ability. Thus, the variations after its speciation from the ancestral species, but conserved in nonfeather-carotenoid birds, may contain the clues related to the new phenotype of feather carotenoids. In the 48 published avian genomes [15], four birds (rifleman, carmine bee-eater, white-tailed tropicbird, and American flamingo) have been found to possess carotenoids in their feathers, while 39 birds showed the absence of carotenoids in a previous study that used HPLC and RS methods [17]. With the Lady Amherst's pheasant and 39 nonfeather-carotenoid birds as background, we selected the lineage-specific nonsynonymous variations in golden pheasant but conserved in the other 40 birds. Finally, we identified 258 genes containing such variations in golden pheasant (Supplementary Table S15). KEGG pathway annotation revealed that the top four scored pathways belonged to “lipid metabolism” (Fig. 4b). The lineage-specific varied genes also contain another lipid transport gene, apolipoprotein B (*APOB*), which is the main apolipoprotein of chylomicrons and low-density lipoproteins (LDL). The biological functions of lipids include the storage and transportation of fat-soluble vitamins, including carotenoids. The transportation of carotenes requires LDL, and the transportation of xanthophylls requires high-density lipoprotein (HDL) [2]. The evolution of those lipid-related genes may change the storage and transportation of carotenoids in golden pheasant, which may be related to the accumulation of carotenoids in its feathers.

In addition, the five feather-carotenoid birds are from five different clades (Passerimorphae, Coraciimorphae, Phaethontimorphae, Phoenicopterimorphae, and Galliformes), indicating that these birds may have independently acquired this ability. To detect whether some genes experience potential convergent variations in feather-carotenoid birds, we separated the birds into two groups, feather-carotenoid and nonfeather-carotenoid, and then performed a whole orthologous gene-wide association study between the two groups. We identified 48 genes containing genotypes (at the amino acid level) that might be associated with the accumulation of carotenoids in feathers (hypergeometric test, *P* < 0.001; Supplementary Table S16). One of these genes, *Zyxin* (*ZYX*), is present at cell-cell contact sites and shuttles to the nucleus, where it affects cell fate and growth [50]. *ZYX* participates in an interaction network with the gamma subfamily of peroxisome proliferator-activated receptor (PPAR-gamma) [51], which is a nuclear hormone receptor that regulates adipocyte differentiation and lipid metabolism [52, 53]. The 48 genes also included four other lipid-associated genes and three genes that overlapped with the lineage-specific varied genes in golden pheasant (Fig. 4b, 4c). These varied genes in golden pheasant and even in more carotenoid birds may be candidate factors of carotenoid deposition in avian plumage, especially lipid-related genes. As a kind of lipochrome, carotenoids are circulated in the same way with lipids. They are packaged into chylomicron fractions *in vivo* and enter and transport in the bloodstream, where they are incorporated with lipoproteins, such as HDL and lLDL [2] (Fig. 4d). Thus, our results indicate that the phenotype of carotenoid deposition in feathers may be controlled or impacted by multiple genes and provide some candidate genes that may associate to this phenotype via a genome-wide comparison.

Transcriptome analysis showed that DEGs between golden pheasant (carotenoid contained) and Lady Amherst's pheasant (non-carotenoid contained) feathers were enriched in the PPAR signaling pathway (Fig. 4e), which mediates the effects of fatty acids and their derivatives [52]. In this pathway, the apolipoprotein gene *APOA*1 was upregulated in golden pheasant plumage (Fig. 4f). In addition, another xanthophyll carotenoid cleavage enzyme gene, *BCO*2, was expressed at a low level in golden pheasant plumage (Fig. 4f). APOA1 is the major protein component of HDL [54], which is the predominant carrier of xanthophylls in plasma [2]. Given the presence of lutein and zeaxanthin and the expression pattern of *APOA*1, APOA1 may be a carotenoid-binding protein in golden pheasant feather follicles. The BCO2 enzyme can cleave xanthophyll carotenoids at 9–10 or 9’-10’ carbon-carbon double bonds [55]. A nonsense mutation or inefficiency of BCO2 results in the abnormal accumulation of carotenoids in livestock adipose tissue [56, 57], primate retina [58], chicken skin [59], and golden-winged warbler feathers [60]. Based on these results, we could hypothesize a process that after transportation into feather follicles, carotenoids bind to APOA1, while the expression of *BCO*2 affects carotenoid deposition (Fig. 4d).

### Connections of β-keratin in plumage coloration and genome quality

Beta-keratins are major components of plumage, and evolution of the β-keratin multigene family may contribute to the novel characteristics of feathers [14, 61]. In the present study, 66 β-keratin genes were identified in the golden pheasant assembly, including 42 feather β-keratins, 9 scale β-keratins, 6 claw β-keratins, and 9 keratinocyte β-keratins. The feather β-keratin occupied the largest proportion in golden pheasant, while the number of claw β-keratins was the least (Supplementary Table S17). The significantly higher expressed genes in feathers were enriched in β-keratins (59 of 827, adjust *P* value = 1.23E-61; Supplementary Table S18). The differentially expressed genes in various colored feathers (white vs iridescent green, white vs red, white vs yellow, iridescent green vs yellow, and iridescent green vs red) were also enriched in β-keratins (Supplementary Table S19). Compared with white feathers, common DEGs in the three other colored feathers included 12 β-keratins that were comprised of one claw β-keratin, three feather β-keratins, three scale β-keratins, and five keratinocyte β-keratins. In addition, common DEGs between carotenoid contained and non-carotenoid contained feathers included three feather β-keratins. These findings suggest that some of the β-keratins may be related to feather colors. The proportions of the four β-keratin subfamilies to the total number of β-keratins were considered to be associated with avian lifestyles in a previous report [14]. In our investigation, to further evaluate the relationship between β-keratin and feather color at the genomic level, we compared the copy number variations of β-keratins in golden pheasant and 51 other avian species. However, no obvious rules have been found between feather color and copy numbers or subfamily proportions compared to other avian species. Nevertheless, the copy numbers of the β-keratin gene were positively correlated with the quality of the assemblies. The coefficient of determination (R2) between β-keratin copy numbers of β-keratin and contig N50 of each genome assembly was 0.77 (*P* value = 1.99E-16, Pearson test; Supplementary Figs. S13, S14). We constructed a phylogenetic tree for the β-keratins of six Galliformes and found that many clades only contained β-keratins from one species and with small divergence (Supplementary Fig. S15). This phylogeny implied there were independent duplication events after the speciation, resulting in young paralogs with high similarity, which may increase the difficulty of the assembly. In the golden pheasant assembly, we found two instances where a feather keratin protein had three alignments in the golden pheasant assembly with sequencing depth of 886 and another feather keratin protein had one alignment with a sequencing depth of 957, which were 9–10 times the mean sequencing depth (92.5) of the whole assembly (Supplementary Table S20). This indicated that there might be 9 and 10 copies of these 2 β-keratins, but that only 3 and 1 copies were assembled, respectively, because of the high similarity among different copies. Therefore, it is likely that the copy number of β-keratins was underestimated in most sequenced birds because of the incomplete genome assembly, particularly for the recently duplicated copies. As a whole, the DEGs indicate that the β-keratins should be related to feather development, but additional genomics comparison is limited because of the underestimation of the actual copy number. As the assembly level increases following the upgrade of sequencing technologies in the future, particularly long-read sequencing technologies, the keratins warrant further comprehensive comparative analysis.

### Conclusions

In the present study, we provided a genome assembly for the golden pheasant and sequenced a genome of Lady Amherst's pheasant with low coverage. Combined with transcriptome analyses, as well as 51 other birds with available genomes, we studied the plumage coloration in *Chrysolophus*. For melanin pigmentation, by identifying the lineage-specific variations in *Chrysolophus*, four melanogenesis genes might be associated with the evolution of eumelanin/pheomelanin regulation in the two pheasants. Additionally, the RNA sequencing (RNA-seq) data showed that the alternative splicing of *ASIP* and *MITF* were consistent with pigment composition in red and yellow feathers of *Chrysolophus*, particularly the *MITF*-1M transcript. For carotenoid pigmentation, we first identified genes that recently varied in golden pheasant but were conserved in the other 40 nonfeather-carotenoid birds, and the results indicated that the evolution of lipid-related genes might be highly related to carotenoid consumption in golden pheasant. Second, by a whole orthologous gene-wide association study between the sequenced feather-carotenoid and nonfeather-carotenoid birds, we identified 48 candidate genes that contain some lipid-related genes directly or indirectly, which may be associated with the carotenoid deposition in a broad range of avian plumage. In addition, the DEGs between the two pheasants were also enriched in some lipid-related pathways. It could be proposed that extraordinarily complex plumage patterns are not only encoded by the genome but also produced by the mechanisms underlying multilayered plumage coloring (Supplementary Fig. S16). As a whole, the present genome comparative results provide some insight into the evolution of color pigmentation, and the transcriptome results show some potentially new regulatory mechanism. However, although the color is easily observed, the visual estimation may not be accurate because of the complex coloration in feathers. The phenotypes quantified by chemical or physical methods should be more accurate and better for further analysis. However, the quantification for a wide range of birds is not feasible at this time, particularly for the eumelanin and pheomelanin, which limits the genomic comparison of plumage coloration in a broad range of avian species. Species models of coloration can provide insight into the evolution and regulation of plumage coloration. Here, we present the golden pheasant and its sister pheasant genomes to serve as candidate models for future studies on plumage coloration.

## Methods

### Genome sequencing and *de novo* assembly

The genomic DNA from blood samples of a male golden pheasant was sequenced on the Illumina Hiseq 2000 platform. A series of paired-end sequencing libraries with insert sizes of 170 bp, 500 bp, 800 bp, 2 kb, 5 kb, 10 kb, and 20 kb were constructed, sequenced, and assembled using SOAPdenovo (SOAPdenovo, RRID:SCR_010752) [62]. Contigs were constructed by adopting the de Bruijn graph-based algorithm from the clean data short-insert reads (∼98.4-fold). Scaffolds were constructed from short reads and long mate-paired information (∼138.06-fold).

Taking advantage of the close evolutionary relationship between golden pheasant and turkey, the turkey genome was used as a reference and linked the assembled genome of golden pheasant to construct pseudochromosomes. The genome of golden pheasant was aligned to the genome of turkey using LASTZ (http://www.bx.psu.edu/miller_lab/dist/README.lastz-1.02.00/README.lastz- 1.02.00a.html). More details about the methods are described in a study of the Chinese rhesus macaque genome [63].

### Genome annotation

Homology-based and RNA-seq combined data were used to annotate coding genes of golden pheasant. For the homology-based prediction, protein sequences of *Gallus gallus, Meleagris gallopavo*, and *Taeniopygia guttata* were downloaded from Ensembl (release 74) and mapped onto the golden pheasant genome using TblastN [64]. Next, high-scoring segment pair (HSP) segments were concatenated between the same pair of proteins by Solar. Homologous genome sequences were then aligned against the matching proteins using Genewise (GeneWise, RRID:SCR_015054) [65] to define accurate gene models. Finally, redundancy was filtered based on the score of the Genewise.

The RNA-seq data are a good supplement for gene annotation because most of the homology alignments have no intact ORFs. Almost 100 G RNA-seq data from 25 samples were used and assembled into transcripts as follows. The reads were mapped to the golden pheasant genome using Tophat (version 2.0.8) [66]. Cufflinks (Cufflinks, RRID:SCR_014597) [67] was used to assemble transcripts. Then, we tried all the possible translations for RNA to protein (three phases for plus and minus strands, respectively) and selected the longest ORF for each transcript. Finally, the Genewise results were extended using the transcript ORFs as the strategy of the Ensembl gene annotation system [68].

Gene functions were assigned according to the best match of the alignment to the public databases, including Swiss-Prot, KEGG, and National Center for Biotechnology Information (NCBI) NR protein databases. Gene Ontology was annotated by Basic Local Alignment Search Tool (BLAST)2GO based on the alignment with the NCBI NR database. The motifs and domains in protein sequences were annotated using InterProScan (InterProScan, RRID:SCR_005829) [36] by searching publicly available databases, including Pfam, PRINTS, PANTHER, PROSITE, ProDom, and SMART.

Tandem repeat searching was carried out using Tandem Repeats Finder [69]. TEs in the genome were predicted by a combination of homology-based and *de novo* approaches. For the homology-based prediction, RepeatProteinMask and RepeatMasker (RepeatMasker, RRID:SCR_012954) [70] against Repbase (http://www.girinst.org/repbase/) [71] were used with default parameters. For the *de novo* approach, RepeatModeler (RepeatModeler, RRID:SCR_015027) and LTR-FINDER (LTR_Finder, RRID:SCR_015247) [72] were used to build the *de novo* repeat library, and then RepeatMasker was used to find TEs in the genome using the *de novo* repeat library. For the comparative analysis, the TEs of chicken, turkey, and zebra finch were annotated using the same pipeline to avoid the influence of different releases of the Repbase database or a different prediction pipeline.

### Transcriptome sequencing

A total of 22 libraries of different organizations or different color feathers from golden pheasants and Lady Amherst's pheasants (detailed descriptions see Additional File 1) were constructed by using the Illumina TruSeq RNA sample preparation kit according to manufacturer's instructions. The libraries (insertion size ∼200 bp) were sequenced 90 bp at each end by using the Illumina Hiseq 2000 platform. We achieved 48∼83 million reads per library (Supplementary Table S21). RNA reads were mapped by Tophat (version 2.0.8) with parameter “-p 6 –b2-very-sensitive –solexa1.3-quals –segment-length 30 –segment-mismatches 2 –read-edit-dist 4 –read-mismatches 4 -r 20 –mate-std-dev 20 –library-type fr-unstranded.” Then, we quantitated the gene expression level by using unique mapped reads and normalized them by using RPKM [73]. For alternative splicing analysis, we quantitated and normalized the junctions by using per million mapped reads. For detecting DEGs between different samples, we used Noiseq [74] with a cutoff probability ≥0.8. The differentially expressed junctions are identified by DEGseq [75] with a MA plot-based method with a random sampling model.

### Phylogenetic analysis and gene family analyses

The Treefam pipeline [76] was used to determine orthologous groups among eight birds (golden pheasant, chicken, turkey, Japanese quail, northern bobwhite, scaled quail, zebra finch, and duck). The detailed steps were performed as follows: (1) protein sequences were mapped by BLASTP (for protein) to identify potential homologous genes; (2) the raw BLASTP results were refined by using Solar, in which the HSPs were conjoined; and (3) similarities between protein sequences were evaluated by using bit-score, followed by clustering protein sequences into gene families by using hcluster_sg, a hierarchical clustering algorithm in the TreeFam pipeline (version 0.50) with the parameters of “-w 5 -s 0.33 -m 100 000.” The identified 6,538 one-to-one orthologous genes among eight species were used to construct the phylogenetic tree. Alignment was performed by using MUSCLE (MUSCLE, RRID:SCR_011812) for the protein sequences and then guided to align the corresponding coding sequences (CDS). A total of 996,755 4D synonymous sites were obtained and used in the phylogenomic construction. The phylogenome was constructed by using RAxML (RAxML, RRID:SCR_006086) (version 8.1.19) [77] with the GTRGAMMA model. The Bayesian relaxed-molecular clock method, implemented in the MCMCTree program [78], was used to estimate the divergence time between golden pheasant and other species. Three calibration time points based on Jarvis's analysis [16], chicken-turkey (28∼29 Mya), chicken-duck (65∼67 Mya), and chicken-zebra finch (88∼90 Mya), were used as constrains in the MCMCTree estimation.

### Positively selected genes and gene family evolution

For the 6,538 one-to-one orthologous paired genes (from the TreeFam pipeline as above described) in the eight avian species, the selected positive genes in golden pheasant were investigated. The protein sequences of orthologues were aligned by using the Muscle [79] software with default parameters. Then, the protein alignment was employed as a guide for aligning CDS. All positions with gaps in the alignments were also removed. Positive selection analysis was conducted by using the refined branch-site model [80], which is implemented in the Codeml program of the PAML (PAML, RRID:SCR_014932) package (version 4) [78]. *P* values were computed by using the Chi-square statistics adjusted by the false discovery rate method to enable multiple testing, and the cutoff was used as 0.01. Further, the selected positive sites were retained by the homology prediction and RNA transcripts to avoid false-positive results from the assembly error or different splicing transcripts.

For the multicopy families, using the gene family results and timed-tree generated in the above as inputs, we studied the expansion and contraction of gene families using the Computational Analysis of Gene Family Evolution (version 2.1) [81], which inferred the dynamics of a gene family under a stochastic birth and death model. The filtering cutoff used the Viterbi *P* value ≤ 0.01.

### SNP and indel detection in Lady Amherst's pheasant

A total of 46.65 Gb paired-end data (read length 100 bp) of the Lady Amherst's pheasant were sequenced from a library with an insert size of 500 bp, and 44.88 Gb high-quality data were generated. All short reads were aligned twice to the golden pheasant genome using SOAP2 (version 2.22) [82]. The first alignment was conducted with an insert size limit of “20 ∼ 1,000 bp.” To reduce the false pair-end alignment, the second alignment was with insert size limit “Median – 3*left-SD (standard deviation) ∼ Median + 3*right-SD.” Based on the alignment, the SNP calling was performed by using SOAPsnp (SOAPsnp, RRID:SCR_010602) [83], which uses a Bayesian model by carefully considering the character of the Solexa sequencing data and experimental factors. Potential SNPs that met the following criteria were filtered: quality score <20 (on the Phred scale) and the total map depth of this location <5 or >120. Based on the pair-end alignment, the 1–5 bp indels variations were identified. To minimize the alignment error, the following set of criteria were applied to the alignment: only one gap, maximum 5 bp, was allowed in a single read; if one read in a pair had a gap in the alignment, the other end had to be gap free, and the orientation and distance had to meet the parameters of the library; no gap was allowed within 5 bp of the ends of a read; no mismatch was allowed within the gap-containing read; and the total map depth of this location was ≥5 and ≤120.

By using the assembly of golden pheasant as a reference, we obtained the putative gene sequences of Lady Amherst's pheasant by changing the assembly of golden pheasant at the homozygous SNP and indel detections. The sites with map depth <5 or >120 are replaced by “N.”

### Lineage-specific varied genes in *Chrysolophus*

In the studies of 48 birds as background [15, 16], the chicken genome was used as a reference, and syntenic orthologous genes between chicken and the other 47 birds were identified. The orthologous relationship was merged using the chicken as a bridge and 8,295 1:1 syntenic orthologous genes among 48 birds were obtained [84]. In the present study, the orthologous gene pairs between the golden pheasant and chicken were identified through the reciprocal best hit and gene synteny relationship. The orthologous genes between golden pheasant and chicken were merged to the orthologous genes of 48 birds, forming orthologous set 1 (OS1) of 52 birds. The genes of 49 birds were clustered by using the TreeFam pipeline and 595 single-copy orthologous families beyond the OS1 were identified. Finally, 8,890 orthologous genes of the 49 birds were obtained by merging the OS1 and the TreeFam single-copy families. We also identified syntenic orthologous genes between Japanese quail and chicken, northern bobwhite and chicken, and scaled quail and chicken, respectively.

After identifying the orthologous gene pairs, we used five other Galliformes (chicken, turkey, Japanese quail, northern bobwhite, and scaled quail) and 11 other birds (duck, zebra finch, carmine bee-eater, bald eagle, little egret, emperor penguin, hoatzin, Anna's hummingbird, common cuckoo, pigeon, and common ostrich) from 11 different clades according to the phylogenetic analysis of 48 birds as background. The orthologous protein sequences of golden pheasant, Lady Amherst's pheasant (putative gene sequences as above described), and the 16 birds were aligned using Muscle. The alignments were compared site by site. We selected the gene containing the site that was same in the 16 birds but specific in *Chrysolophus*. To avoid false-positive results from different splicing transcripts, only the results supported by both the homology prediction and RNA transcripts were retained.

### Carotenoid accumulation related genes

Previous investigations have displayed the avian species that did or did not express carotenoid in their feathers [17]. These referenced species overlapped with the genomes published for 48 birds [15, 16], resulting in 4 carotenoid-containing and 39 non-carotenoid-containing species with constructed assemblies. Based on these studies, comparative analyses were performed to explore the carotenoid accumulation related candidate genes in golden pheasant by using two strategies. Given the close relationship and the difference in carotenoid utilization between the two *Chrysolophus* species, lineage-specific varied genes in golden pheasant as well as conserved in Lady Amherst's pheasant and the other 39 non-carotenoid birds were selected. A multisequences alignment was conducted by using MUSCLE to select the genes containing pheasant-specific sites, which is common in Lady Amherst's and the other 39 birds. Finally, 258 recent varied genes were annotated to KEGG pathways and scored by the following methods: if pathway A has total number of N(a) genes in golden pheasant and there are number n(a) genes in the 258 recent varied genes, then the score of pathway A is S(A) + = n(a)/N(a), and if pathway B has number of O(ab) genes shared with pathway A and has total number of N(b) genes in golden pheasant, then there are number of e(b) genes in pathway B, except the members shared with pathway A, and the S(A) + = e(b)/N(b)*O(ab)/N(a).

Otherwise, we referred to the population resequencing analysis strategy, such as that in Hilma Holm's research [85], and divided 45 birds and golden pheasant into carotenoid and non-carotenoid groups. The two uncertain birds with bright yellow or orange or red feathers (golden-collared manakin and bar-tailed trogon) were classified into carotenoid experiential. The genotypes of the carotenoid birds were examined for randomness among all species by using a hypergeometric site by site test with a *P* value < 0.001.

### Keratin family analysis

To avoid bias from different prediction methods applied in different bird genomes, we downloaded protein sequences of keratin genes of chicken from NCBI and then mapped against golden pheasant and 52 other avian genomes by using the same pipeline. Homology-based gene prediction was obtained by using the gene prediction pipeline mentioned above, except the threshold alignment rate was greater than 50%. Domain annotation was performed by using InterProScan, and only the results with domain of IPR003461 (avian keratin), IPR002957 (Type I keratin), or IPR003054 (Type II keratin) were retained. The correlation between the copy number of β-keratin and the assembly quality (contig N50) refers to the studies of the 48 birds (part of “Correlation between average substitution rates and number of species within different avian orders” and “color Discriminability”) [15]. The subfamilies of β-keratins were classified based on the best hit to the β-keratins of zebra finch, which had been classified by Greenwold [86]. We also performed another version of this study by using the keratin gene numbers from evolutionary research of the keratins in the 48 birds [14].

### Pigment identification

Both melanin and carotenoid pigments in feathers were examined in two ways: spectrum and chromatogram. Raman spectroscopy was carried out using a Labram HR1800 spectrometer (HORIBA JobinYvon, France), referring to the strategy of Galvan [87] and Thomas [49] for melanin and carotenoid detection, respectively. HPLC was carried out using an SIL-20A HPLC system equipped with an SPD-20A UV/Vis detector (Shimadzu, Japan), referring to the strategy of McGraw [88] and Wakamatsu [34] for melanin and carotenoid detection, respectively. More details of pigment identification are described in Supplementary Notes 1.

## Availability of supporting data

The genome assembly is available via GenBank. The *Chrysolophus pictus* genome assembly has been deposited under the accession number SAMN02980944 (BioProject PRJNA257945). Raw Illumina sequencing reads of the *C. pictus* reference genome have been deposited at NCBI in the SRA under accession number SRA753467. The *C. pictus* RNA-seq reads have been deposited at NCBI in the SRA under accession number SRA743586. The *C. amherstiae* RNA-seq reads have been deposited at NCBI in the SRA under accession number SRA743973. Supporting data, including assembly and annotation files, have been deposited in the *GigaScience* database GigaDB [89].

## Additional files

**Additional file 1:** This doc file contains the supplementary figures: S1–S20.

**Additional file 2:** This xls file contains the supplementary tables: S1-S24.

**Additional file 3:** This doc file contains supplementary notes of pigments identification, animal sampling and transcriptome analysis.

## Competing Interests

The authors declare that they have no competing financial interests.

## Abbreviations

α-MSH: alpha-melanocyte-stimulating hormone; 4D: 4-fold degenerate; ASIP: agouti signaling protein; ATRN: attractin; BLAST: Basic Local Alignment Search Tool; CDS: corresponding coding sequence; CMC: ; DEG: differentially expressed gene; EDNRB: endothelin receptor B; GO: ; HDL: high-density lipoprotein; HPLC: high-performance liquid chromatography; HSP: high-scoring segment pair; indels: insertions and deletions; KEGG: Kyoto Encyclopedia of Genes and Genomes; KIT: KIT proto-oncogene tyrosine-protein kinase; LDL: low-density lipoprotein; MAPK: mitogen-activated protein kinase; MC1R: melanocortin-1 receptor; MITF: ; MULE: ; Mya: million years ago; NCBI: National Center for Biotechnology Information; ORF: open reading frame; OS: orthologous set; PPAR-gamma: peroxisome proliferator-activated receptor; RE: repetitive element; RNA-seq: RN

## Author contributions

G.P.L., J.Y., and C.Z. conceived the study. J.Y., G.Q.G., Y.C.Z., J.G., and X.K.Y. prepared the samples. M.X., J.M.M., Y.L.Y., H.M.C., and C.Z. performed genome sequencing, assembly, and annotation. G.P.L. and C.Z. supervised genome sequencing, assembly, and annotation. M.X., G.Q.G., Y.L.Y., J.M.M., X.Q.Z., X.M.X., and J.Y.X. performed genome analyses. G.Q.G., M.X., C.L.B., and G.H.S. carried out the transcriptome analyses. G.Q.G., Z.Y.W., and Y.H. carried out carotenoids and eu-/pheomelanins analysis. R.B.W., G.P.Z., and C.M.C. discussed the data. All authors contributed to data interpretation. G.P.L., G.Q.G., and M.X. wrote the paper with significant contributions from Y.C.Z., C.L.B., J.Y., C.M.C., and C.Z.

## Ethics approval and consent to participate

This study was approved by the Institutional Animal Care and Use Committee of the Inner Mongolia University.

## Funding

This research was partly funded by the State Key Development Program for Basic Research of China, 973 Program (2012CB22306), the Open Project of Key Development Program for Basic Research of Inner Mongolia Autonomous Region, National Natural Science Foundation of China (31660301), Natural Science Foundation of Inner Mongolia (2013ZD06), and State Key Laboratory of Agricultural Genomics (2011DQ782025).

## Supplementary Material

GIGA-D-18-00007_(Original_Revision).pdf

GIGA-D-18-00007_Revision_1.pdf

GIGA-D-18-00007_Revision_2.pdf

Response_to_Reviewer_Comments_Report_(Original_Submission).pdf

Response_to_Reviewer_Comments_Report_Revision_1.pdf

Reviewer_1_Report_(Original_Submission) -- Christopher Emerling 2/12/2018 Reviewed

Reviewer_1_Report_Revision_1 -- Christopher Emerling 7/8/2018 Reviewed

Reviewer_2_Report_(Original_Submission) -- Matthew Greenwold 4/1/2018 Reviewed

Reviewer_2_Report_Revision_1 -- Matthew Greenwold 7/18/2018 Reviewed

Supplemental Files
