# Supplementary material for: Comparative genomics and transcriptomics of Chrysolophus provide insights into the evolution of complex plumage coloration
Source: Gigascience. 2018 Sep 6;7(10):giy113. doi: 10.1093/gigascience/giy113 (PMC6204425; doi:10.1093/gigascience/giy113)
Supplement: GIGA-D-18-00007_(Original_Revision).pdf [file giy113_giga-d-18-00007_(original_revision).pdf]

GigaScience

Comparative genomics and transcriptomics of Chrysolophus provide insights into the evolution of complex plumage coloration

--Manuscript Draft--

**Manuscript Number:**	GIGA-D-18-00007
**Full Title:**	Comparative genomics and transcriptomics of Chrysolophus provide insights into the evolution of complex plumage coloration
**Article Type:**	Research
**Funding Information:**	State Key Development Program for Basic Research of China, 973 Program (2012CB22306) — Prof. Guangpeng Li<br>he Open Project of Key Development Program for Basic Research of Inner Mongolia Autonomous Region, National Natural Science Foundation of China (30960244) — Prof. Guangpeng Li<br>Natural Science Foundation of Inner Mongolia (2013ZD06) — Dr. Yongchun Zuo<br>State Key Laboratory of Agricultural Genomics (2011DQ782025) — Dr. Chi Zhang
**Abstract:**	Background: As one of the most recognizable characteristics in birds, plumage color has a high impact on understanding evolution and mechanisms of coloration. Feather and skin are ideal tissues to explore the genomics and complexity of color patterns in vertebrates. Both two species of the genus Chrysolophus, golden pheasant (Chrysolophus pictus) and Lady Amherst's pheasant (Chrysolophus amherstiae), exhibit brilliant colors in their plumage, but with extremely phenotypic differences. This makes the two species can be of great models to investigate plumage coloration mechanisms in birds.<br>Results: We sequence and assemble a genome of golden pheasant with high-coverage and annotate 15,552 protein-coding genes. The genome of Lady Amherst's pheasant was sequenced with low-coverage. Based on the feather pigments identification, a series of genomic and transcriptomic comparisons are conducted to investigate the complex features of plumage coloration. Through identifying the lineage-specific sequence variations in Chrysolophus and golden pheasant, against different background, we find that four melanogenesis biosynthesis genes and lipid related genes may be candidate genomic factors for the evolution of their melanin and carotenoid pigmentation, respectively. In addition, a whole orthologous genes wide association study among 47 birds shows some candidate genes related to carotenoid coloration in a broad range of birds. The transcriptome data further reveal some important regulators of the two colorations, especially the MITF-1M splicing for the pheomelanin synthesis.<br>Conclusions: Analysis of the golden pheasant and its sister pheasant genomes, as well as comparing with other avian genomes, are helpful to reveal the underlying regulation of their plumage coloration. This study provides important genomic information and insights for further study of avian plumage evolution and diversity.
**Corresponding Author:**	Meng Xu<br>BGI<br>Shenzhen, Guangdong CHINA
**Corresponding Author Secondary Information:**	
**Corresponding Author's Institution:**	BGI
**Corresponding Author's Secondary Institution:**	
**First Author:**	Meng Xu

Powered by Editorial Manager® and ProduXion Manager® from Aries Systems Corporation

**First Author Secondary Information:**	
**Order of Authors:**	Meng Xu
	Guangqi Gao
	Yongchun Zuo
	Yulan Yang
	Chunling Bai
	Junyang Xu
	Zhuying Wei
	Jiumeng Min
	Guanghua Su
	Xianqiang Zhou
	Jun Guo
	Yu Hao
	Guiping Zhang
	Xukui Yang
	Xiaomin Xu
	Randall B Widelitz
	Cheng-Ming Chuong
	Chi Zhang
	Jun Yin
	Guangpeng Li
**Order of Authors Secondary Information:**	
**Opposed Reviewers:**	
**Additional Information:**	
**Question**	**Response**
Are you submitting this manuscript to a special series or article collection?	No
**Experimental design and statistics**<br><br>Full details of the experimental design and statistical methods used should be given in the Methods section, as detailed in our Minimum Standards Reporting Checklist. Information essential to interpreting the data presented should be made available in the figure legends.<br><br>Have you included all the information requested in your manuscript?	Yes
**Resources**<br><br>A description of all resources used,	Yes

including antibodies, cell lines, animals and software tools, with enough information to allow them to be uniquely identified, should be included in the Methods section. Authors are strongly encouraged to cite Research Resource Identifiers (RRIDs) for antibodies, model organisms and tools, where possible.<br><br>Have you included the information requested as detailed in our Minimum Standards Reporting Checklist?	
**Availability of data and materials**<br><br>All datasets and code on which the conclusions of the paper rely must be either included in your submission or deposited in publicly available repositories (where available and ethically appropriate), referencing such data using a unique identifier in the references and in the "Availability of Data and Materials" section of your manuscript.<br><br>Have you have met the above requirement as detailed in our Minimum Standards Reporting Checklist?	Yes

Comparative genomics and transcriptomics of Chrysolophus provide insights into the evolution of complex plumage coloration

Guangqi Gao[1,2†], Meng Xu[3†], Yongchun Zuo[1,2†], Yulan Yang[3†], Chunling Bai[1,2†], Junyang Xu[3†], Zhuying Wei[1,2], Jiumeng Min[3], Guanghua Su[1,2], Xianqiang Zhou[3], Jun Guo[4], Yu Hao[4], Guiping Zhang[3], Xukui Yang[3], Xiaomin Xu[3], Randall B Widelitz[5], Cheng-Ming Chuong[5], Chi Zhang[3*], Jun Yin[4*], Guangpeng Li[1,2*]

[1]The State key Laboratory of Reproductive Regulation and Breeding of Grassland Livestock, Inner Mongolia University, Hohhot, 010070, China.

[2]College of Life Science, Inner Mongolia University, Hohhot, 010070, China.

[3]BGI Genomics, BGI-Shenzhen, Shenzhen 518083, China

[4]College of Life Science, Inner Mongolia Agricultural University, Hohhot, 010018, China.

[5]Department of Pathology, Keck School of Medicine, University of Southern California, Los Angeles, CA 90033, USA.

[†]Co-first author

[*]Correspondence: gpengli@imu.edu.cn, zhangchi2@genomics.cn, yinjun@imau.edu.cn.

Abstract

Background: As one of the most recognizable characteristics in birds, plumage color has a high impact on understanding evolution and mechanisms of coloration. Feather and skin are ideal tissues to explore the genomics and complexity of color patterns in vertebrates. Both two species of the genus *Chrysolophus*, golden pheasant (*Chrysolophus pictus*) and Lady Amherst's pheasant (*Chrysolophus amherstiae*),

exhibit brilliant colors in their plumage, but with extremely phenotypic differences. This makes the two species can be of great models to investigate plumage coloration mechanisms in birds.

Results: We sequence and assemble a genome of golden pheasant with high-coverage and annotate 15,552 protein-coding genes. The genome of Lady Amherst's pheasant was sequenced with low-coverage. Based on the feather pigments identification, a series of genomic and transcriptomic comparisons are conducted to investigate the complex features of plumage coloration. Through identifying the lineage-specific sequence variations in *Chrysolophus* and golden pheasant, against different background, we find that four melanogenesis biosynthesis genes and lipid related genes may be candidate genomic factors for the evolution of their melanin and carotenoid pigmentation, respectively. In addition, a whole orthologous genes wide association study among 47 birds shows some candidate genes related to carotenoid coloration in a broad range of birds. The transcriptome data further reveal some important regulators of the two colorations, especially the MITF-1M splicing for the pheomelanin synthesis.

Conclusions: Analysis of the golden pheasant and its sister pheasant genomes, as well as comparing with other avian genomes, are helpful to reveal the underlying regulation of their plumage coloration. This study provides important genomic information and insights for further study of avian plumage evolution and diversity.

Keywords: genome, transcriptome, *Chrysolophus*, plumage, coloration

Background

The plumage colors of birds serve functions in crypsis, social signaling and mate choice (1). Due to the diversity of colors and easy to observe, plumage provides an ideal model to explore the formation and genomic evolution of coloration patterns in animals.

Studies on birds and mammals suggested that the integument colors are regulated by several mechanisms. Melanin, which is produced by neural crest cell-derived melanocytes, is the major contributor of pigmentation in avian feathers and mammalian hairs (2). Black and brown feathers are derived from the deposition of the eumelanin, whereas reddish and light-yellow feathers are caused by pheomelanin. Carotenoids are chemicals for vitamin synthesis, and act as anti-oxidants for the immune system (2). Some birds can use dietary derived carotenoids to produce yellow, orange and red in their feathers, such as lutein, zeaxanthin, β-cryptoxanthin, and β-carotene (3). Red colors may also come from other rare pigments, such as porphyrins in black-shouldered kites (4), psittacofulvins in parrots (5), iron oxide in *Gypaetus barbatus* and turacin in *Tauraco macrorhynchus* (6). In addition, feather coloration may also be a result of specific structures that combine with non-iridescent colors and iridescent metal lusters (2).

Feather complex coloration should be under the coordination of multiple genes regulating diverse mechanisms. The biosynthetic pathway of melanogenesis has been elucidated (7, 8), and former studies have revealed the DNA polymorphisms of dozens of genes that are lead to variations in melanin-based coloration (9). But some details regulating the switch of eu-/pheomelanin remain to be resolved (10). Some candidate genes for carotenoid-related functions in mammals and invertebrates were documented, and some of their homologous genes may also present in birds (11). However, the production metabolism of carotenoid pigments has not been well characterized. Besides, the nanostructural colors of feathers are deemed related to keratinization and affected by keratin genes (12, 13). But the keratins are belong to a huge family with dozens to nearly two hundred members in birds (14) and which one (or ones) plays the key role still be unknown. Genome information could provide new perspectives to study the

mechanisms of bird coloration. In 2014, the most extensive comparative analysis of avian species at the genome level to date was published and revealed two genes have a negative correlation between color discriminability and *dN/dS* across birds (15, 16). But their work included only 15 genes without distinguishing melanin, carotenoid, or other pigments. Based on this, we thought that more genomic research could be done to investigate the candidate molecular mechanisms of avian plumage coloration.

In this study, we focused on the plumage coloration issues of golden pheasant (*Chrysolophus pictus*) at genomic and transcriptomic level. And with its sister species, the Lady Amherst's pheasant (*Chrysolophus amherstiae*), are two important organisms for the study of plumage coloration because of their phenotypic differences and close relationship which can even cross breed to produce fertile offspring under human feeding condition. In adult male golden pheasant, the crest and rump feathers are both golden-yellow in color, the belly and upper tail coverts are dark red, the nape feathers are light orange with two black stripes, the mantle is iridescent green, and the tail is black spotted with cinnamon (Figure 1, Additional file 1: Figure S1). The golden pheasant is an accepted colorful avian species with distinct brilliant feather colors in adult males, which can be observed with obvious characteristics of melanin and carotenoid pigments. By comparison, for the adult male Lady Amherst's pheasants, the red and yellow feathers only distribute in small parts of the body including crest, rump and upper tail coverts, while most of the other body parts are white or black (Figure 1, Additional file 1: Figure S1). Carotenoids were present in the yellow back feathers of golden pheasant, but were uncertain in Lady Amherst's pheasant (17). In this study, we sequenced the genome and transcriptome of these two pheasants, and identified the melanin and carotenoid pigments in plumages of two pheasants using High Performance Liquid Chromatography (HPLC) and Raman spectroscopy (RS) methods.

Then we performed comprehensive comparative analysis with other 48 sequenced avian references (15) at a suitable level to investigate the evolution of plumage coloring of golden pheasant or *Chrysolophus*.

Results and discussions

Genome assembly and annotation

The genomic DNA of golden pheasant was extracted using blood genomic DNA from a male adult from Foping National Nature Reserve in Shaanxi and fed in Jilin of China. A series of paired-end libraries with different insert size were constructed and sequenced using Illumina Hiseq 2000 platform (Additional file 2: Table S1). The *de novo* assembly size was 1.029 Gb, with a contig N50 size of 34.4 Kb and scaffold N50 size of 1.55 Mb, respectively (Table 1). Assembly quality was assessed by aligning the total small insert size reads (170bp ~ 800bp) back to the assembly. These reads covered 99.92% of the genome, and 99.17% of the alignment could be mapped by over 10 reads (Additional file 2: Tables S3; Additional file 1: Figure S2). In addition, the assembly covered more than 95.71% of the transcriptome-assembled transcripts (102,426 out of 107,012, Additional file 2: Tables S4), indicating that the golden pheasant genome was of high quality. To have a global view of some potential specific elements in golden pheasant, 93.9% of the assembly was linked to pseudo-chromosomes using the turkey chromosomes as a reference (Figure 2a). The genomic DNA from a male Lady Amherst's pheasant was sequenced with a relatively low coverage (approximately 43×). We identified 7.26 million SNPs and 0.45 million InDels (1-5 bp per InDel, total 0.83 Mb length) (Additional file 2: Table S5) in Lady Amherst's pheasant using the assembly of golden pheasant as reference, indicating the divergence between these two pheasants is about 0.84%. Moreover, the golden pheasant genome was used as a reference to align the transcriptome sequences from the two species. The average mapping rates of golden

pheasant and Lady Amherst's pheasant are 85.82% and 81.83%, respectively. These results imply a close relationship between the two species.

Combining the homology-based and transcriptome-assisted methods, 15,552 protein-coding genes were identified in the assembly of golden pheasant, of which 98.69% were homologous to public databases (Swissprot, Nr, and KEGG) (Additional file 2: Table S6), and 89.43% were supported by transcriptome sequences (RPKM > 1 in at least one sample). Moreover, repetitive elements (REs) comprised around 10.93% of the golden pheasant genome, with the chicken repeat 1 (CR1) elements being the most abundant class (83.14% of REs; 0.093 Gb), which was similar to chicken (Additional file 2: Table S7). The satellite DNAs expanded in golden pheasant genome, which were 5.5 and 18.2 folds to the chicken and zebra finch genome, respectively (Additional file 1: Figure S3; Additional file 2: Table S8). There was no lineage-specific REs identified in golden pheasant, but similar evolutionary trend of DNA/CMC and DNA/MULE transposable elements (TEs) were found between the golden pheasant and turkey (Additional file 2: Table S8). More and more data were provided to support that TEs might play a role as candidate gene expression regulators, especially in modulation of abutting gene expression (18-20). Thus, genes within 2kb up- and downstream of these TEs were focused. The flanking genes of the satellite DNAs, CMC, and MULE could be enriched in sodium-potassium exchange ATPase activity (GO: 0005391, Adjust P-value = 0.02295), cell development (GO: 0048468, Adjust P-value = 1.93E-08), and kidney development (GO: 0001822, Adjust P-value =0.00128) respectively (Additional file 2: Table S9-11). The functional enrichment showed that the specific or expanded REs may be involved in some adaptive evolution of golden pheasant or turkey.

Evolution analysis within Galliformes

The phylogenetic placement is a critical background for many comparative genomic

analyses. To assess the phylogenetic position of the golden pheasant in Galliformes, phylogenetic tree was constructed with two other sequenced Galliformes (chicken (21) and turkey (22)), the sequenced Anseriformes (duck (23)) which is closest to Galliformes, and a model species (zebra finch (24)) as an outgroup. According to the taxonomy browser of NCBI, golden pheasant has the closest relationship with chicken by the morphological studies. However, a recent molecular phylogeny study showed that golden pheasant was closer to turkey, but they only used 12 mitochondrial genes and not all branches with 100% bootstrap support (BS) (25). Here, we confirmed that the golden pheasant is taxonomically closer to turkey with 100% BS with the data of 8,079 single-copy orthologous genes (Figure 2b, Additional file 1: Figure S5a). Another study concluded that protein-coding genes might reflect life history traits more than phylogeny topology (16). Therefore, a phylogeny tree was constructed based on 1,487,987 4-fold degenerate sites (4D sites) which do not affect the amino acids coding and is usually considered to subject selective pressure less. This tree was same with the former one and also with 100% BS (Additional file 1: Figure S5b). The relationship was consistent with the above REs analysis that golden pheasant and turkey had the similar divergence distribution (Additional file 1: Figure S3) and shared some common specific REs, which belong to non-coding regions (Additional file 2: Table S8). Thus, our results resolved the uncertain phylogenetic placement of golden pheasant. Furthermore, the divergence time of the golden pheasant and turkey was estimated to be approximately 13 million years ago using MCMCTree (Figure 2b). .

Sequence divergences and/or gene duplications have been proposed as important mechanisms in the course of evolution (26). The positive Darwinian selection is a universal strategy to find candidate clues of adaptive evolution at the DNA sequence level. For the 8079 single-copy orthologous genes in five birds (three Galliformes

species: golden pheasant, chicken, turkey, and other two related species: duck and zebra finch), 688 positive selected genes were identified in golden pheasant using branch site model (Additional file 2: Table S12 and S13). For the multi-copy families, 31 lineage specific gene families were identified in golden pheasant (Figure 2c) by hierarchical clustering. Besides, we identified 102 expanded and 24 contracted gene families respectively through a maximum likelihood framework (Additional file 2: Table S14 and S15). The expanded cytochrome P450 (CYP) family gains two more CYP2D copies in golden pheasant, and this result was further verified in another 9 or even 48 avian species (Figure 2d, Additional file 1: Figure S6). The CYP enzymes use molecular oxygen to modify substrate structure, participate a huge number of physiological, ecological and toxicological processes, including oxidative metabolism of steroid hormones, fatty acids, drugs, and environmental pollutants (27). In human, CYP2D6 is one of most extensively studied member, which is responsible for about 25% of the metabolism of known drugs (28). The CYPs are also considered as good candidates for carotenoid ketolases (29). Recently, two studies found CYP2J19 which belongs to same clan with CYP2D, associated with carotenoid-based coloration phenotypes in zebra finches and canaries respectively (29, 30). The expanded CYP2D6 genes may benefit the metabolism or biotransformation of some foreign chemicals, and a candidate evolution factor for carotenoid deposition in feathers of golden pheasant.

Specific mutation and alternative splicing of melanin genes in *Chrysolophus*

Melanin is the most common and widespread pigment in avian feathers, and yield black, gray, brown, rufous and buff shades and patterns (2). Both two *Chrysolophus* species possessed darker eumelanic and brighter pheomelanic color in their integument plumage, especially the most impressive bright red and yellow feathers in male individuals (Figure 1a). A previous investigation concluded that human hairs with six

different colors varying from black, brown, to red, all contain both eumelanin and pheomelanin but their proportions determine the visual colors (31). Black hair has the highest level of eumelanin and highest combine ratio of eumelanin/pheomelanin, with red hair has the highest level of pheomelanin and highest combine ratio of pheomelanin/eumelanin. Our HPLC also showed that the colored feathers from different parts of golden pheasant and Lady Amherst's pheasant contained two melanins at the same time (Additional file 1: Figure S7). Indicating the clear feather colors of two pheasants may result from the relatively extreme mixture ratio of eu-/pheomelanin. Based on these information, we paid more attention to genetic regulations to eu-/pheomelanin switch in male *Chrysolophus* birds from both genomic and transcriptomic perspectives.

We firstly identified the lineage specific varied genes in *Chrysolophus* by comparing the genomes of *Chrysolophus* and other 13 avian species which belong to 13 different clades in the phylogeny tree of the 48 birds (16). We found that four melanogenesis associated genes have specific mutated sites in *Chrysolophus* species, including attractin (ATRN), endothelin receptor B (EDNRB), KIT proto-oncogene tyrosine-protein kinase (KIT), and agouti signaling protein (ASIP) (Figure 3a). ATRN has at least 8 sites under positive selective with $\omega>1$ (BEB test, $P > 0.98$) (32) which could prevent formation of the "Kelch repeat type 1" domain (PF01344) based on the InterProScan annotation (33) (Additional file 1: Figure S8). The EDNRB has a three amino acids deletion in the "G protein-coupled receptor, rhodopsin-like" domain (PF00001, Additional file 1: Figure S9) and the KIT has a two amino acids deletion in the C-terminal regions, which are conserved in other birds and even in green anole (Additional file 1: Figure S10). In ASIP gene, a single nucleotide inserts after the initiation codon at exon 2A, which might disable this initiation codon or cause

frameshift resulting in a premature transcription termination at the thirteenth cordon (Figure 3b). Anyway, both cases would impact 50% kind of ASIP isoforms. The melanogenesis is under multiple levels of complex regulation, mainly through the transcriptional and post-transcriptional regulation of microphthalmia-associated transcription factor (MITF) gene which can stimulate the transcription of genes that function in producing melanin (34-37). The classic transcriptional regulator of MITF is the melanocortin-1 receptor (MC1R) with its ligands, alpha-melanocyte-stimulating hormone (α-MSH) and ASIP. The ASIP can competitively antagonize α-MSH to bind MC1R, and ATRN is an obligatory accessory receptor for ASIP and enhance ASIP-Mc1R binding (34). From another aspect, KIT can mediate phosphorylation of MITF protein at Ser73 through mitogen activated protein kinase (MAPK) pathway, trigger a short-lived MITF activation as well as an ubiquitin-dependent proteolysis (35, 36). Besides, EDNRB stimulation can not only activate MITF expression but also elicit MAPK-mediated MITF phosphorylation (37). As locating in the upstream of melanogenesis pathway, variations of these four genes may amplify the biosynthesis or switches of eumelanin and pheomelanin through a signaling cascade (35, 36), resulting in a more extreme mixture ratio of eu-/pheomelanin in *Chrysolophus*..

Gene mutations can alter plumage color traits among different birds, but the diversity of colors and patterning present in one individual may due to gene expression or alternative splicing (38). We sequenced the RNA of feather follicles from different body parts in two pheasants respectively. We found the ASIP exists at least 10 splicing isoforms (Additional file 2: Table S16), in which ASIP-1A isoforms are highly expressed in red-pheomelanin feathers, while ASIP-1F isoforms are abundant in yellow-pheomelanin feathers (Figure 3c). MITF, another central regulatory element of the melanogenesis pathway, regulates at least 11 melanogenesis genes directly or non-

directly through the feedback loops (39), also has complex alternative splicing pattern in *Chrysolophus* feather follicles. The MITF consists of at least 13 exons and two ORFs which are translated from exon-1B and exon-1M (Figure 3d; Additional file 2: Table S17). Interestingly, the expression of MITF-1M exon in six red/yellow feathers was significantly higher than that in the six black/white feathers (fold_change=3.80, adjust_P=1.35e-11, Figure 3d). This result indicates that the MITF-1M isoform may be a key factor to regulate pheomelanin synthesis in feather follicles of *Chrysolophus*.

Carotenoid utilization in *Chrysolophus* plumage

Carotenoids, a class of organic fat-soluble compound, are synthesized by plants, bacteria or fungi, and utilized by animals through their diet (40). Depending on the chemical structure, these pigments usually appear yellow, orange or red in avian plumage (2). In our study, both pheasant species have yellow to red plumage, but carotenoids were only found in golden pheasant. Raman spectroscopy (RS) (41) showed carotenoid bands in golden pheasant feathers but not in Lady Amherst's pheasant feathers (Additional file 1: Figure S11a and b). Further identification by HPLC revealed that these carotenoids included lutein and zeaxanthin (Figure 4a, Additional file 1: Figure S12, Additional file 2: Table S18).

The two other sequenced Galliformes, chicken and turkey, also can't accumulate carotenoids in feathers. It seems like that golden pheasant acquires this new ability. So the variations after its speciation from the ancestral species, but conserved in non-feather-carotenoid birds, may contain the clues related to the newly phenotype of feather carotenoids. In the 48 published avian genomes (15), four birds (Rifleman, Carmine Bee-eater, White-tailed Tropicbird and American Flamingo) have been revealed the presence of carotenoids in their feathers and 39 birds are absent, by a former research using HPLC and RS methods (17). With the Lady Amherst's pheasant

and 39 non-feather-carotenoid birds as background, we picked the genotype which was lineage-specific in golden pheasant, but same in other 40 birds. Finally, we identified 258 genes containing these variations in golden pheasant (Additional file 2: Table S19). With the KEGG pathway annotation, we found that the top four scored pathways were all belong to "Lipid metabolism" (Figure 4b). Except these four pathway, there is another lipid transport gene, apolipoprotein B (APOB), is the main apolipoprotein of chylomicrons and low density lipoproteins (LDL). The biological functions of lipids include the storage and transportation of fat-soluble vitamin, including carotenoid. Such as the transportation of carotenes need low density lipoprotein (LDL) and transportation of xanthophylls need high density lipoprotein (HDL) [2]. The evolution of those lipid related genes may change the storage and transportation of carotenoids in golden pheasant, which may be related to the accumulation of carotenoids in its feathers.

Another intriguing thing is that, the five feather-carotenoid birds are from five different clades (Passerimorphae, Coraciimorphae, Phaethontimorphae, Phoenicopterimorphae, and Galliformes), indicating they may get the ability independently. To detect if there are some genes experiencing potential convergent variations in feather-carotenoid birds, we separated the birds into two groups: feather-carotenoid and non-feather-carotenoid, and then performed a whole orthologous genes wide association study between the two groups. As a result, we identified 48 genes containing genotype that might associate to accumulate carotenoids in feather (hypergeometric test, $P < 0.001$, Table S20). One of the genes, Zyxin (ZYX) gene, has been shown to be present at cell-cell contact sites and are known to shuttle into the nucleus where they can affect cell fate and growth (42). ZYX has interaction network with the gamma subfamily of peroxisome proliferator-activated receptor (PPAR-gamma) gene (43) which is a nuclear hormone receptors, and regulates adipocytic

differentiation and lipid metabolism (44, 45). The 48 genes also included other four lipid associated genes and three genes which are overlapped with the lineage-specific varied genes in golden pheasant (Figure 4b, 4c). Our results showed some candidate genes that may associate with the evolution of carotenoids deposition in avian plumage.

Transcriptome analysis showed that differentially expressed genes (DEGs) between golden pheasant (carotenoid contained) and Lady Amherst's pheasant (non-carotenoid contained) feathers were enriched in PPAR signaling pathway (Figure 4e) which mediate the mediate the effects of fatty acids and their derivatives (44). In this pathway, the apolipoprotein gene APOA1 was up-regulated in golden pheasant plumage (Figure 4f). Besides, another xanthophyll carotenoid cleavage enzyme gene, BCO2, was expressed at a low level in golden pheasant plumage (Figure 4f). APOA1 is the major protein component of HDL(46), which is the predominant carrier of xanthophylls in plasma (2). Given the presence of lutein and zeaxanthin, and the expression pattern of APOA1 gene, it could be speculated that APOA1 may be a carotenoid binding protein (CBP) in golden pheasant feather follicles. BCO2 enzyme can cleave xanthophyll carotenoids at the 9-10 or 9'-10' carbon-carbon double bonds (47). A nonsense mutation or inefficiency of BCO2 has been shown to result in the abnormal accumulation of carotenoids in livestock adipose tissue (48, 49), primate retina (50), chicken skin (51) and golden-winged warbler feathers (52). Based on these results, we could hypothesize a process that after transported into feather follicles, carotenoids bind to APOA1 to settle down while expression of BCO2 affect carotenoid deposition (Figure 4d).

Connections of β-keratin in plumage coloration and genome quality

Keratins are major components of plumage, and evolution of keratin multigene family is thought to have contributed to the novel characters of feathers (14, 53). In our study, the significantly higher expressed genes in feathers were enriched in β-keratins (59 out

of 827, adjust P-value=7.21E-62, Additional file 2: Table S21). And the differentially expressed genes in various color feathers (white vs iridescent green, white vs red, white vs yellow, iridescent green vs yellow, and iridescent green vs red) were also enriched in β-keratins (adjust P-value < 1E-10, Additional file 2: Table S22). This suggests that some of the β-keratins may be related to feather colors. To further detect the relationship between β-keratin and feather color at the genomic level, we did comparison about the copy number variations of β-keratins in golden pheasant and other 48 avian species. However, we found that the copy numbers of the β-keratin gene were positively correlated with the quality of assemblies. The coefficient of determination (R^2) between β-keratin copy numbers of β-keratin and contig N50 of each genome assembly was 0.47 (P=1.92e-07, Pearson's test, Additional file 1: Figure S13, S14). Moreover, one feather keratin proteins had three alignments in the golden pheasant assembly with sequencing depth of 886, and another feather keratin protein had one alignment with a sequencing depth of 957. These were 9-10 times of the mean sequencing depth (92.5) of the whole assembly (Additional file 2: Table S24). This indicates that there may be nine and ten copies of the two keratins respectively, but only assembled three and one copies because of the high similarity among different copies. Therefore, it seems like that the copy number of β-keratins was underestimated in most sequenced birds because of the incomplete genome assembly. As a whole, the DEGs indicate the β-keratins should be related to feather development, but the further genomics comparison is limit because of underestimate of real copy number. As these assembly level rises following the upgrading of sequencing technologies in the future, especially the long-read sequencing technologies, the keratins should be worth for comprehensive comparative analysis.

Conclusions

In this study, we provided a genome assembly for the golden pheasant. We also

sequenced a genome of Lady Amherst's pheasant with low-coverage and transcriptomes from the two pheasants. Besides, with the help of other 48 sequenced birds, we did multi-level comparison analysis to investigate the plumage coloration in golden pheasant or *Chrysolophus*. For melanin pigmentation, through identifying the lineage specific variations in *Chrysolophus*, we found four genes locating in the upstream of three different regulator paths of melanogenesis biosynthesis, which might be associated to evolution of eumelanin/pheomelanin colors in two pheasants' feather. Meanwhile, the RNA-seq data showed that the alternative splicing of ASIP and MITF were in accordance with pigment composition in red and yellow feathers of *Chrysolophus,* especially the MITF-1M transcript. For carotenoid pigmentation, we firstly identified genes which varied recently in golden pheasant but conserved in other 40 non-feather-carotenoid birds, and the results indicated that the evolution of lipid related genes may be highly related to the carotenoids consumption in golden pheasant. Secondly, by a whole orthologous genes wide association study between the sequenced feather-carotenoid and non-feather-carotenoid birds, we found 48 candidate genes that contain some lipid-related genes directly or indirectly, which may be associated to the carotenoids deposition in a broad range of avian plumage. In addition, the DEGs between the two pheasants also enriched in some lipid-related pathways. As a whole, our genome comparative results provide some insight into the evolution of color pigmentation and the transcriptome results show some potential newly regulatory mechanism. On the other hand, although the color is easy to observe, the visual estimation may be not accurate in many times because of the complex coloration in feathers. The phenotypes quantified by chemical or physical methods should be more accurate and better for further analysis. However, the quantifying for a wide range of birds is not enough so far, especially for the eumelanin and pheomelanin, which limit

the genomic comparative of plumage coloration in a broad range of avian species. Good models can provide a lot of help, and because of they are external features and close relationship, the golden pheasant and its sister pheasant should be another good model to investigate the evolution and regulation of plumage coloration.

Methods

Genome sequencing and *de novo* assembly

The genomic DNA from blood samples of a male golden pheasant was sequenced on Illumina Hiseq 2000 platform. A series of paired-end sequencing libraries with insert sizes of 170 bp, 500 bp, 800 bp, 2 kb, 5 kb, 10 kb and 20 kb was constructed, sequenced and assembled using SOAPdenovo (54). Contigs were constructed by adopting the de Bruijn graph-based algorithm from the clean data short-insert reads (~98.4-fold). Scaffolds were constructed from short reads and long mate-paired information (~138.06-fold).

Taking advantage of the close evolutionary relationship between golden pheasant and turkey, the turkey genome was used as a reference and linked the assembled genome of golden pheasant to construct pseudochromosomes. The genome of golden pheasant was aligned to the genome of turkey using LASTZ (http://www.bx.psu.edu/miller_lab/dist/README.lastz-1.02.00/README.lastz-1.02.00a.html). More details about the method are described in the study of Chinese rhesus macaques genome (55).

Genome annotation

Homology-based and RNA-seq combined data were used to annotate coding genes golden pheasant. For the homology-based prediction, protein sequences of *Gallus*

gallus, *Meleagris gallopavo* and *Taeniopygia guttata* were downloaded from Ensembl (release 74) and mapped onto the golden pheasant genome using TblastN (56). Secondly, high-scoring segment pairs (HSPs) segments were concatenated between the same pair of proteins by Solar. Thirdly, homologous genome sequences were aligned against the matching proteins using Genewise (57) to define accurate gene models. Finally, redundancy was filtered based on the score of the Genewise.

The RNA-seq data are good supplement for gene annotation because most of the homology alignments have no intact ORFs. Almost 100G RNA-seq data from 25 samples were used and assembled them into transcripts as follows. Firstly the reads were mapped to the golden pheasant genome using Tophat (58). Secondly, Cufflinks (59) was used to assemble transcripts. Thirdly, the longest ORF from six kinds of phase was selected. Finally, the Genewise's results were extended using the transcripts ORFs as the strategy of Ensembl gene annotation system (60).

Gene functions were assigned according to the best match of the alignment to the public databases, including Swiss-Prot, KEGG and NCBI NR protein databases. Gene Ontology was annotated by Blast2GO based on the alignment with NCBI NR database. The motifs and domains in protein sequences were annotated using InterProScan (33) by searching publicly available databases, including Pfam, PRINTS, PANTHER, PROSITE, ProDom, and SMART.

Tandem repeat searching was carried out using Tandem Repeats Finder (61). Transposable elements (TEs) in the genome were predicted by a combination of homology-based and *de novo* approaches. For the homology-based prediction,

RepeatProteinMask and RepeatMasker (62) against Repbase (http://www.girinst.org/repbase/) (63) were used with default parameters. For the *de novo* approach, RepeatModeler and LTR-FINDER (64) were used to build the *de novo* repeat library, and then RepeatMasker was used to find TEs in the genome using the *de novo* repeat library. For the comparative analysis, the TEs of chicken, turkey and zebra finch were annotated using the same pipeline to avoid the influence of different release of Repbase database or different prediction pipeline.

Transcriptome sequencing

22 libraries of different organizations or different color feathers from golden pheasants and Lady Amherst's pheasants (detailed descriptions see Additional file 1) were constructed using the Illumina TruSeq RNA sample preparation kit according to manufacturer's instructions. The libraries (insertion size ~200 bp) were sequenced 90 bp at each end using Illumina Hiseq 2000 platform. We achieved 48~83 million reads per library (Additional file 2: Table S28). RNA reads were mapped by Tophat and subsequently analyzed with in-house Perl scripts. We quantitated the gene expression level using unique mapped reads and normalized using per kilobase of transcript per million mapped reads (RPKM) (65). For alternative splicing analysis, we quantitated the junctions using per million mapped reads (RPM). For detecting differentially expressed genes (DEGs) between different individual samples, we used a method described by Chen et al (66). In this study, we defined DEGs using two criterions: a) RPKM is at least two-fold difference; b) the false discovery rate (FDR) is less than 0.001. For detecting DEGs between different groups which can contain multiple

samples, we used Noiseq (67) with cutoff Probability >= 0.8. The differentially expressed junctions are identified by DEGseq(68) with MA-plot-based method with Random Sampling model.

Phylogenetic analysis and gene family ananlysis

Treefam pipeline (69) was used to determine orthology groups among five birds (golden pheasant, chicken, turkey, zebra finch, Peking duck). The detailed steps as follows: 1) protein sequences were mapped by BLASTP and to identify potential homologous genes; 2) the raw BlastP results were refined using Solar by which the HSPs were conjoined; 3) similarity between protein sequences were evaluated using bit-score, followed by clustering protein sequences into gene families using hcluster_sg, a hierarchical clustering algorithm in the Treefam pipeline (version 0.50) with the parameters of "-w 5 -s 0.33 -m 100000". The identified 8,079 one-to-one orthologous genes among five species were used to construct phylogenetic tree. Alignment was performed using MUSCLE for the protein sequences firstly, then guided to align corresponding coding sequences (CDS). A total of 1,487,987 fourfold degenerate (4D) synonymous sites were obtained and were used to in the phylogenomics construction. The phylogenomics was constructed using RAxML (version 8.1.19) (70) with the "GTRGAMMA" model. The Bayesian relaxed-molecular clock (BRMC) method, implemented in the MCMCTree program (71), was used to estimate the divergence time between golden pheasant and other species. Three calibration time points based on Jarvis's analysis (16), chicken-turkey (28~29 Mya), chicken-Peking duck (65~67 Mya) and chicken-zebra finch (88~90 Mya), were used as constrains in the MCMCTree

estimation.

Positively selected genes and gene family evolution

For the 8,079 one-to-one orthologous paired genes (from the Treefam pipeline as above described) in the golden pheasant, chicken, turkey, Peking duck and zebra finch, the positive selected genes in golden pheasant were investigated. The protein sequences of orthologs were aligned using the Muscle (72) software with default parameters. Then, the protein alignment was employed as a guild for aligning CDS. All positions with gaps in the alignments were also removed. Positive selection analysis was conducted using the refined branch-site model (73) which is implemented in the codeml program of PAML package (version 4) (71). P-values were computed using the Chi-square statistics adjusted by the false discovery rate (FDR) method to allow for multiple testing and the results were filtered with adjusted P-value > 0.01. Further, the positive selected sites were retained by the homology prediction and RNA transcripts to avoid the false positive.

For the multi-copies families, CAFE (version 2.1) (74) was used to detect the gene family evolution in golden pheasant. The gene family results from Treefam pipeline and the estimated divergence time between species were employed as inputs with the Viterbi P-value ≤ 0.01.

SNP and InDel detection in Lady Amherst's pheasant

A total of 46.65Gb paired-end data (reads length 100bp) of the Lady Amherst's pheasant were sequenced from a librariy with insert size of 500bp and 44.88Gb clean data were generated. All the short reads were aligned twice to the golden pheasant

genome using SOAP2 (version 2.22) (75). The first alignment was with the insert size limit “20 ~ 1000bp”. Aiming to reduce the false pair-end alignment, the second alignment was with insert size limit “Median – 3*left-SD (standard deviation) ~ Median + 3*right-SD”. Base on the alignment**,** the single nucleotide polymorphism (SNP) calling was performed by means of SOAPsnp (76), which uses a Bayesian model by carefully considering the character of the Solexa sequencing data and experimental factors. Potential SNPs were filtered that met the following criteria: 1) quality score <20 (on the Phred scale); 2) the total map depth of this location <5 or >120. Based on the pair-end alignment, the 1-5bp insertion/deletion variations were identified. In order to minimize the alignment error, the following set of criteria were applied to the alignment: 1) only one gap, maximum 5-bp, was allowed in a single read; 2) if one read in a pair had a gap in the alignment, the other end had to be gap-free, and the orientation and distance had to meet the parameters of the library; 3) no gap was allowed within 5-bp of the ends of a read; 4) no mismatch was allowed within the gap-containing read; 5) the total map depth of this location≥5 and ≤120.

Using the assembly of golden pheasant as reference, we get the putative gene sequences of Lady Amherst’s pheasant by changing the assembly of golden pheasant at the homozygous SNP and Indel detections. The sites with map depth <5 or >120 are replaced by “N”.

Lineage specific mutated genes in *Chrysolophus*

Using the available genome of 48 birds as background (15, 16), the lineage specific mutated genes were searched in *Chrysolophus*. The research of 48 birds had identified

the orthologous gene pairs between chicken and other 47 birds respectively and constructed the orthologous relationship of the 48 birds through merging pair-wise orthologs according to the chicken reference. The orthologous genes pairs between the golden pheasant and chicken were identified through the reciprocal best hit (RBH) and gene synteny relationship as the method in the 48 birds study. Finally, the orthologous genes between golden pheasant and chicken were merged to the orthologous of 48 birds, forming the orthologous set 1 (OS1) of 49 birds. Besides, the genes of 49 birds were clustered using the TreeFam pipeline and identified 595 single copy orthologous families beyond the OS1. Finally, 8,890 orthologous genes of the 49 birds were obtained by merging the OS1 and the TreeFam single copy families. The protein sequences of orthologs were aligned using the Muscle. The alignments were compared site by site to select the site which is same in other three Galloanseres birds (chicken, turkey, and peking duck) and other ten birds from ten different clades (zebra finch, Carmine bee-eater, bald eagle, little egret, emperor penguin, hoatzin, anna's hummingbird, common cuckoo, pigeon, and common ostrich) but specific in *Chrysolophus*. To avoid the false positive from annotation, the results were retained which were both supported by the homology prediction and RNA transcripts.

Carotenoids accumulation related genes

Previous investigations have displayed the avian species which expressed carotenoid in their feathers or not (17). These referenced species overlapped with the 48 genome published birds (15, 16) resulted four carotenoid contained and 39 non-carotenoid contained species with constructed assemblies. Based on these studies, comparative

analyses were performed to explore the carotenoid accumulation related candidate genes in golden pheasant using the two follow strategies. 1) Given the close relationship and the difference of carotenoid utilizing between the two *Chrysolophus* species. Genes with recent variations in golden pheasant against Lady Amherst's pheasant and the other 39 non-carotenoid birds were selected. Firstly, the putative gene sequences of the Lady Amherst's pheasant were identified through the homozygous SNPs and InDels. Then a multi-sequences alignment was carried out using MUSCLE to select the genes which contain the pheasant specific sites which is common in Lady Amherst's and other 39 birds. Finally, the total of 258 recent varied genes were annotated to the KEGG pathways and scored by the following methods: i) if pathway A has total number of N(a) genes in golden pheasant and there are number n(a) genes in the 258 recent varied genes. The score of pathway A, S(A) += n(a)/N(a). ii) if pathway B has number of O(ab) genes shared with pathway A and has total number of N(b) genes in golden pheasant. There are number of e(b) genes in pathway B but except the shared members with pathway A. the S(A) += e(b)/N(b)*O(ab)/N(a). The results show that the top four scored pathways are belong to "Lipid metabolism" (Figure 4c).

Otherwise, we refer to the population re-sequencing analysis strategy, like Hilma Holm's research (77), divided 45 published birds and golden pheasant into carotenoid and non-carotenoid groups. The two uncertain birds with bright yellow or orange or red feathers (Golden-collared manakin and Bar-tailed trogon) were classified into carotenoid experiential. The genotypes of the carotenoid birds were tested the randomness among the all species using hypergeometric test site by site with the P-

value < 0.001.

Keratin family analysis

To avoid bias from different prediction methods applied in different bird genomes, we download protein sequences of keratin genes of chicken from NCBI, and then mapped against golden pheasant and other 48 bird genomes using the same pipeline. Homology-based gene prediction was taken using the gene prediction pipeline mentioned above, except for the threshold that alignment rate was greater than 50%. We did domain annotation using InterProScan and only retained the results with domain of IPR003461 (avian keratin), IPR002957 (Type I keratin), or IPR003054 (Type II keratin). The correlation between the copy number of beta-keratin and the assembly quality (contig N50) refer to the method in 48 birds' research (part of "Correlation between average substitution rates and number of species within different avian orders" and "Color Discriminability") (15). We also did another version using the keratin genes number from evolution research of keratins in 48 birds (14).

Pigments identification

Both melanin and carotenoid pigments in feathers were examined in two ways, methods of spectrum and chromatogram. Raman spectroscopy was carried out through a Labram HR1800 spectrometer (HORIBA JobinYvon, France), referring the strategy of Galvan (78) and Thomas (41) for melanin and carotenoid detection, respectively. High-performance liquid chromatography was carried out through an SIL-20A HPLC system equipped with an SPD-20A UV/Vis detector (Shimadzu, Japan), referring the strategy of McGraw (79) and Wakamatsu (31) for melanin and carotenoid detection, respectively.

More details of pigment identification are described in Additional file 3: Notes 1.

Additional files

Additional file 1: This doc file contains the supplementary figures: S1–S17.

Additional file 2: This xlsx file contains the supplementary tables: S1-S28.

Additional file 3: This doc file contains supplementary notes of pigments identification, animal sampling and Transcriptome analysis.

ACKNOWLEDGEMENTS

We owe many thanks to Dr. Kazumasa Wakamatsu from Fujita Health University for providing the TTCA and PTCA standards. We are indebted to Cai Li and Hailin Pan from China National GeneBank for giving advices in genome analysis. We are also grateful to Tianyuan Wang from Yuanfeng wild animal farm (Jilin province, China) for taking care of the experimental birds. Many thanks to the people whose names are not included in the author list, but did some contribution to this project.

FUNDING

This research was partly funded by the State Key Development Program for Basic Research of China, 973 Program (2012CB22306), the Open Project of Key Development Program for Basic Research of Inner Mongolia Autonomous Region, National Natural Science Foundation of China (30960244), Natural Science Foundation of Inner Mongolia (2013ZD06). State Key Laboratory of Agricultural Genomics（No. 2011DQ782025）

DATA AVAILABILITY

Genome assemblies have been deposited in GenBank. The *Chrysolophus pictus* genome assembly has been deposited under the accession number SAMN02980944.

Author's contributions

GPL, JY and CZ conceived the study. JY, GQG, YCZ, JG and XKY prepared the samples. MX, JMM, YLY HMC and CZ performed genome sequencing, assembly and annotation. GPL and CZ supervised genome sequencing, assembly and annotation. MX, GQG, YLY, JMM, XQZ, XMX and JYX performed genome analyses. GQG, MX, CLB and GHS carried out the transcriptome analyses. GQG, ZYW and YH carried out carotenoids and eu-/pheomelanins analysis. RBW, GPZ and CMC discussed the data. All authors contributed to data interpretation. GPL, GQG and MX wrote the paper with significant contributions from YCZ, CLB, JY, CMC and CZ.

Competing interests

The authors declare no competing financial interests.

Ethics approval and consent to participate

This study was approved by the Institutional Animal Care and Use Committee of the Inner Mongolia University.

References

1. Hill G & McGraw K (2006) *Bird coloration Vol. 2. Function and Evolution* (Harvard University Press, Cambridge, Massachusetts).
2. Hill G & McGraw K (2006) *Bird coloration Vol. 1. Mechanisms and measurements* (Harvard University Press, Cambridge, Massachusetts).
3. Prum RO, LaFountain AM, Berro J, Stoddard MC, & Frank HA (2012) Molecular diversity, metabolic transformation, and evolution of carotenoid feather pigments in cotingas (Aves:

Cotingidae). *J Comp Physiol B* 182(8):1095-1116.
4. Negro JJ, Bortolotti GR, Mateo R, & Garcia IM (2009) Porphyrins and pheomelanins contribute to the reddish juvenal plumage of black-shouldered kites. *Comp Biochem Physiol B Biochem Mol Biol* 153(3):296-299.
5. McGraw KJ & Nogare MC (2005) Distribution of unique red feather pigments in parrots. *Biol Lett* 1(1):38-43.
6. Toral GM, Figuerola J, & Negro JJ (2008) Multiple ways to become red: pigment identification in red feathers using spectrometry. *Comp Biochem Physiol B Biochem Mol Biol* 150(2):147-152.
7. Slominski A, Tobin DJ, Shibahara S, & Wortsman J (2004) Melanin pigmentation in mammalian skin and its hormonal regulation. *Physiol Rev* 84(4):1155-1228.
8. Schiaffino MV (2010) Signaling pathways in melanosome biogenesis and pathology. *Int J Biochem Cell Biol* 42(7):1094-1104.
9. Roulin A & Ducrest AL (2013) Genetics of colouration in birds. *Semin Cell Dev Biol* 24(6-7):594-608.
10. Simon JD, Peles D, Wakamatsu K, & Ito S (2009) Current challenges in understanding melanogenesis: bridging chemistry, biological control, morphology, and function. *Pigment Cell Melanoma Res* 22(5):563-579.
11. Walsh N, Dale J, McGraw KJ, Pointer MA, & Mundy NI (2012) Candidate genes for carotenoid coloration in vertebrates and their expression profiles in the carotenoid-containing plumage and bill of a wild bird. *Proc Biol Sci* 279(1726):58-66.
12. Maia R, Macedo RH, & Shawkey MD (2012) Nanostructural self-assembly of iridescent feather barbules through depletion attraction of melanosomes during keratinization. *J R Soc Interface* 9(69):734-743.
13. Ng CS*, et al.* (2012) The chicken frizzle feather is due to an alpha-keratin (KRT75) mutation that causes a defective rachis. *PLoS Genet* 8(7):e1002748.
14. Greenwold MJ*, et al.* (2014) Dynamic evolution of the alpha (alpha) and beta (beta) keratins has accompanied integument diversification and the adaptation of birds into novel lifestyles. *BMC Evol Biol* 14:249.
15. Zhang G*, et al.* (2014) Comparative genomics reveals insights into avian genome evolution and adaptation. *Science* 346(6215):1311-1320.
16. Jarvis ED*, et al.* (2014) Whole-genome analyses resolve early branches in the tree of life of modern birds. *Science* 346(6215):1320-1331.
17. Thomas DB*, et al.* (2014) Ancient origins and multiple appearances of carotenoid-pigmented feathers in birds. *Proc Biol Sci* 281(1788):20140806.
18. Marino-Ramirez L, Lewis KC, Landsman D, & Jordan IK (2005) Transposable elements donate lineage-specific regulatory sequences to host genomes. *Cytogenet Genome Res* 110(1-4):333-341.
19. Naito K*, et al.* (2009) Unexpected consequences of a sudden and massive transposon amplification on rice gene expression. *Nature* 461(7267):1130-1134.
20. Bolger A*, et al.* (2014) The genome of the stress-tolerant wild tomato species Solanum pennellii. *Nat Genet* 46(9):1034-1038.
21. Wallis JW*, et al.* (2004) A physical map of the chicken genome. *Nature* 432(7018):761-764.
22. Dalloul RA*, et al.* (2010) Multi-platform next-generation sequencing of the domestic turkey (Meleagris gallopavo): genome assembly and analysis. *PLoS Biol* 8(9).

23. Huang Y, *et al.* (2013) The duck genome and transcriptome provide insight into an avian influenza virus reservoir species. *Nat Genet* 45(7):776-783.

24. Warren WC, *et al.* (2010) The genome of a songbird. *Nature* 464(7289):757-762.

25. Li HM, Shi JP, Zeng DL, Zeng ZH, & Qin XM (2011) The complete mitochondrial genome of Chrysolophus pictus (Galliformes: Phasianidae) and a phylogenetic analysis with related species. *Mitochondrial DNA* 22(5-6):159-161.

26. Kondrashov FA (2012) Gene duplication as a mechanism of genomic adaptation to a changing environment. *Proc Biol Sci* 279(1749):5048-5057.

27. Kubota A, *et al.* (2011) Cytochrome P450 CYP2 genes in the common cormorant: Evolutionary relationships with 130 diapsid CYP2 clan sequences and chemical effects on their expression. *Comp Biochem Physiol C Toxicol Pharmacol* 153(3):280-289.

28. Ingelman-Sundberg M (2005) Genetic polymorphisms of cytochrome P450 2D6 (CYP2D6): clinical consequences, evolutionary aspects and functional diversity. *Pharmacogenomics J* 5(1):6-13.

29. Mundy NI, *et al.* (2016) Red Carotenoid Coloration in the Zebra Finch Is Controlled by a Cytochrome P450 Gene Cluster. *Curr Biol* 26(11):1435-1440.

30. Lopes RJ, *et al.* (2016) Genetic Basis for Red Coloration in Birds. *Curr Biol* 26(11):1427-1434.

31. Ito S, *et al.* (2011) Usefulness of alkaline hydrogen peroxide oxidation to analyze eumelanin and pheomelanin in various tissue samples: application to chemical analysis of human hair melanins. *Pigm Cell Melanoma R* 24(4):605-613.

32. Yang Z, Wong WS, & Nielsen R (2005) Bayes empirical bayes inference of amino acid sites under positive selection. *Mol Biol Evol* 22(4):1107-1118.

33. Jones P, *et al.* (2014) InterProScan 5: genome-scale protein function classification. *Bioinformatics* 30(9):1236-1240.

34. Hida T, *et al.* (2009) Agouti protein, mahogunin, and attractin in pheomelanogenesis and melanoblast-like alteration of melanocytes: a cAMP-independent pathway. *Pigment Cell Melanoma Res* 22(5):623-634.

35. Levy C, Khaled M, & Fisher DE (2006) MITF: master regulator of melanocyte development and melanoma oncogene. *Trends Mol Med* 12(9):406-414.

36. Wu M, *et al.* (2000) c-Kit triggers dual phosphorylations, which couple activation and degradation of the essential melanocyte factor Mi. *Genes Dev* 14(3):301-312.

37. Sato-Jin K, *et al.* (2008) Epistatic connections between microphthalmia-associated transcription factor and endothelin signaling in Waardenburg syndrome and other pigmentary disorders. *FASEB J* 22(4):1155-1168.

38. Moroy T & Heyd F (2007) The impact of alternative splicing in vivo: mouse models show the way. *RNA* 13(8):1155-1171.

39. Poelstra JW, *et al.* (2014) The genomic landscape underlying phenotypic integrity in the face of gene flow in crows. *Science* 344(6190):1410-1414.

40. Toews DP, Hofmeister NR, & Taylor SA (2017) The Evolution and Genetics of Carotenoid Processing in Animals. *Trends Genet* 33(3):171-182.

41. Thomas DB, McGraw KJ, James HF, & Madden O (2014) Non-destructive descriptions of carotenoids in feathers using Raman spectroscopy. *Anal Methods-Uk* 6(5):1301-1308.

42. Marie H, *et al.* (2003) The LIM protein Ajuba is recruited to cadherin-dependent cell junctions through an association with alpha-catenin. *J Biol Chem* 278(2):1220-1228.

43. Li B & Trueb B (2001) Analysis of the alpha-actinin/zyxin interaction. *J Biol Chem* 276(36):33328-33335.

44. Hihi AK, Michalik L, & Wahli W (2002) PPARs: transcriptional effectors of fatty acids and their derivatives. *Cell Mol Life Sci* 59(5):790-798.

45. Savage DB (2005) PPAR gamma as a metabolic regulator: insights from genomics and pharmacology. *Expert Rev Mol Med* 7(1):1-16.

46. Poelstra JW, Ellegren H, & Wolf JBW (2013) An extensive candidate gene approach to speciation: diversity, divergence and linkage disequilibrium in candidate pigmentation genes across the European crow hybrid zone. *Heredity* 111(6):467-473.

47. Mein JR, Dolnikowski GG, Ernst H, Russell RM, & Wang XD (2011) Enzymatic formation of apo-carotenoids from the xanthophyll carotenoids lutein, zeaxanthin and beta-cryptoxanthin by ferret carotene-9',10'-monooxygenase. *Arch Biochem Biophys* 506(1):109-121.

48. Vage DI & Boman IA (2010) A nonsense mutation in the beta-carotene oxygenase 2 (BCO2) gene is tightly associated with accumulation of carotenoids in adipose tissue in sheep (Ovis aries). *Bmc Genet* 11.

49. Tian R, Pitchford WS, Morris CA, Cullen NG, & Bottema CDK (2010) Genetic variation in the beta, beta-carotene-9 ', 10 '-dioxygenase gene and association with fat colour in bovine adipose tissue and milk. *Anim Genet* 41(3):253-259.

50. Li BX*, et al.* (2014) Inactivity of human beta,beta-carotene-9 ', 10 '-dioxygenase (BCO2) underlies retinal accumulation of the human macular carotenoid pigment. *P Natl Acad Sci USA* 111(28):10173-10178.

51. Eriksson J*, et al.* (2008) Identification of the Yellow skin gene reveals a hybrid origin of the domestic chicken. *Plos Genetics* 4(2).

52. Toews DP*, et al.* (2016) Plumage Genes and Little Else Distinguish the Genomes of Hybridizing Warblers. *Curr Biol* 26(17):2313-2318.

53. Greenwold MJ & Sawyer RH (2010) Genomic organization and molecular phylogenies of the beta (beta) keratin multigene family in the chicken (Gallus gallus) and zebra finch (Taeniopygia guttata): implications for feather evolution. *BMC Evol Biol* 10:148.

54. Li R*, et al.* (2010) De novo assembly of human genomes with massively parallel short read sequencing. *Genome Res* 20(2):265-272.

55. Yan G*, et al.* (2011) Genome sequencing and comparison of two nonhuman primate animal models, the cynomolgus and Chinese rhesus macaques. *Nat Biotechnol* 29(11):1019-1023.

56. Kent WJ (2002) BLAT - The BLAST-like alignment tool. *Genome Res* 12(4):656-664.

57. Birney E, Clamp M, & Durbin R (2004) GeneWise and genomewise. *Genome Res* 14(5):988-995.

58. Trapnell C, Pachter L, & Salzberg SL (2009) TopHat: discovering splice junctions with RNA-Seq. *Bioinformatics* 25(9):1105-1111.

59. Trapnell C*, et al.* (2010) Transcript assembly and quantification by RNA-Seq reveals unannotated transcripts and isoform switching during cell differentiation. *Nat Biotechnol* 28(5):511-U174.

60. Curwen V*, et al.* (2004) The Ensembl automatic gene annotation system. *Genome Res* 14(5):942-950.

61. Benson G (1999) Tandem repeats finder: a program to analyze DNA sequences. *Nucleic Acids Research* 27(2):573-580.

62. Chen N (2004) Using RepeatMasker to identify repetitive elements in genomic sequences. *Curr*

Protoc Bioinformatics* Chapter 4:Unit 4 10.
63. Jurka J, *et al.* (2005) Repbase Update, a database of eukaryotic repetitive elements. *Cytogenet Genome Res* 110(1-4):462-467.
64. Xu Z & Wang H (2007) LTR_FINDER: an efficient tool for the prediction of full-length LTR retrotransposons. *Nucleic Acids Res* 35(Web Server issue):W265-268.
65. Mortazavi A, Williams BA, McCue K, Schaeffer L, & Wold B (2008) Mapping and quantifying mammalian transcriptomes by RNA-Seq. *Nat Methods* 5(7):621-628.
66. Chen S, *et al.* (2010) De novo analysis of transcriptome dynamics in the migratory locust during the development of phase traits. *PLoS One* 5(12):e15633.
67. Tarazona S, Garcia-Alcalde F, Dopazo J, Ferrer A, & Conesa A (2011) Differential expression in RNA-seq: a matter of depth. *Genome Res* 21(12):2213-2223.
68. Wang L, Feng Z, Wang X, Wang X, & Zhang X (2010) DEGseq: an R package for identifying differentially expressed genes from RNA-seq data. *Bioinformatics* 26(1):136-138.
69. Li H, *et al.* (2006) TreeFam: a curated database of phylogenetic trees of animal gene families. *Nucleic Acids Res* 34(Database issue):D572-580.
70. Stamatakis A (2006) RAxML-VI-HPC: maximum likelihood-based phylogenetic analyses with thousands of taxa and mixed models. *Bioinformatics* 22(21):2688-2690.
71. Yang Z (2007) PAML 4: phylogenetic analysis by maximum likelihood. *Mol Biol Evol* 24(8):1586-1591.
72. Wheeler TJ & Kececioglu JD (2007) Multiple alignment by aligning alignments. *Bioinformatics* 23(13):i559-568.
73. Zhang J, Nielsen R, & Yang Z (2005) Evaluation of an improved branch-site likelihood method for detecting positive selection at the molecular level. *Mol Biol Evol* 22(12):2472-2479.
74. De Bie T, Cristianini N, Demuth JP, & Hahn MW (2006) CAFE: a computational tool for the study of gene family evolution. *Bioinformatics* 22(10):1269-1271.
75. Li R, *et al.* (2009) SOAP2: an improved ultrafast tool for short read alignment. *Bioinformatics* 25(15):1966-1967.
76. Li R, *et al.* (2009) SNP detection for massively parallel whole-genome resequencing. *Genome Res* 19(6):1124-1132.
77. Holm H, *et al.* (2011) A rare variant in MYH6 is associated with high risk of sick sinus syndrome. *Nat Genet* 43(4):316-320.
78. Galvan I, *et al.* (2013) Raman spectroscopy as a non-invasive technique for the quantification of melanins in feathers and hairs. *Pigment Cell Melanoma Res* 26(6):917-923.
79. McGraw KJ, Hill GE, Stradi R, & Parker RS (2002) The effect of dietary carotenoid access on sexual dichromatism and plumage pigment composition in the American goldfinch. *Comp Biochem Physiol B Biochem Mol Biol* 131(2):261-269.

Figure legends

Fig.1 Profile of golden pheasant (upper right) and Lady Amherst's pheasant (upper left) and their feathers from different body parts (lower part). Both male

species (near) are more colorful than females (far). The female feathers are represented by the napes.

Fig. 2 Comparative genomic analysis among the golden pheasant and other avian species. (a) Global view of the golden pheasant genome using the pseudochromosomes. **(b)** The Maximum Likelihood phylogenetic relationships of the golden pheasant in Galloanseres. The tree was constructed based on 1.49 Mb 4-fold degenerate sites, from 8,079 single-copy orthologous genes among four sequenced Galloanseres genomes (golden pheasant, chicken, turkey, and duck) and the zebra finch (as outgroup). **(c)** Venn diagram of the shared orthologous groups among the five species. **(d)** Two more copies of CYP2D gene in golden pheasant. The background species are selected based on Jarvis's phylogeny for the 48 avian genomes, of which three Galloanseres birds and other ten birds from ten different clades are selected in this analysis.

Fig. 3 The variation and alternative splicing of some regulator genes in the eu-/pheomelanin synthesis metabolism. (a) The pathway of Eu-/pheomelanin synthesis metabolism. The lineage specific variation genes in *Chrysolophus* are marked by red star. The significant higher expressed genes in feathers with green, red, and yellow color, are marked by the colorful rectangular respectively, all use the white feathers (A-F-Hackle and A-F-Breast) as control. **(b)** The single nucleotide insertion in the ASIP gene of the *Chrysolophus*. A base of adenine inserts after the initiation codon of the ORF at exon 2A. This insertion was verified in another five *Chrysolophus* individuals (lower part). "SN", sample name; "ST", sequencing type, R, RNA sequencing, D, DNA sequencing; "Ta/To", the number of reads support the shown genotype/the number of total mapped reads. **(c)** The RNA alternative splicing of ASIP gene. The upper section

is the alternative splicing models of ASIP. Rectangles represent exons, and curves represent junctions between the exons. The size scale ratio between exons and introns is 1:10. The lower section is expression histogram of the junctions. RPM (Reads per million mapped reads) was used to normalize expression levels. The color of the column matches the accepter exon color. The color of the footstone matches the donor exon color. **(d)** The RNA alternative splicing of MITF gene. Descriptions are same with Fig. 3c. The description of sample name: “P-”, golden pheasant; “A-”, Lady Amherst’s pheasant; “-F-”, feather; “-S-”, skin; “-Fe-F”, feather from female; “-**”, body part.

Fig.4 The comparative analysis and RNA expression of the carotenoid accumulation in feather. (a) The high performance liquid chromatography (HPLC) analysis of lutein and zeaxanthin in *Chrysolophus* red and yellow feathers. **(b)** The KEGG pathway annotation of the genes which is lineage-specific in golden pheasant but same in other non-feather-carotenoid 40 birds. The scoring standard of each pathway is described in the method. **(c)** The orthologous genes wide association study to the carotenoids accumulation. The coordinates are based on chicken chromosomes. Dashed line indicates the gene has more than one sites with $P < 0.001$. The green spots are genes that also contain recent varied sites. The orange spots are lipid related genes. **(d)** The theoretical process of carotenoids transportation and deposition. **(e)** The KEGG pathway enrichment of the union DEGs between feather follicles of the two pheasants. The “RichFactor” = the number of DEGs in this pathway/the number of gene set in this pathway. More details are described in Additional file 3: Notes 4.2. **(f)** The expression of the APOA1 and BCO2 gene in the two pheasants.

Table 1 Statistics of assembly and annotation for the golden pheasant genome

Genome characteristics	Data
Assembly features	
Estimate of genome size	1,032,423,981 bp
Total size of assembled scaffolds	1,028,603,357 bp
Scaffold N50	1,547,393 bp
Longest scaffold	18,323,375 bp
Total size of assembled contigs	1,003,285,807 bp
Contig N50	34,356 bp
Longest contig	257,270 bp
GC content (excluding Ns)	40.80%
Annotation features	
Number of gene models	15,552
Mean coding sequence length	1705.27 bp
Mean number of exons per gene	9.94
Mean exon length	171.62 bp
Mean intron length	2397.68 bp
Total size of REs	112,429,773 bp
REs share in genome	10.93%

*RE, repetitive elements.

 Click here to download Figure Figures.docx

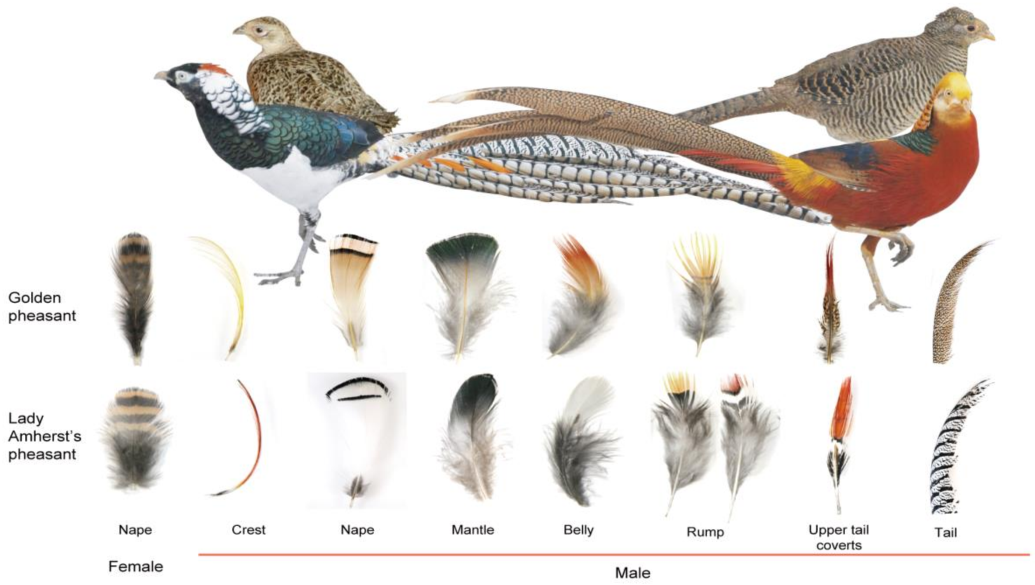

Fig.1 Profile of golden pheasant (upper right) and Lady Amherst's pheasant (upper left) and their feathers from different body parts (lower part). Both male species (near) are more colorful than females (far). The female feathers are represented by the napes.

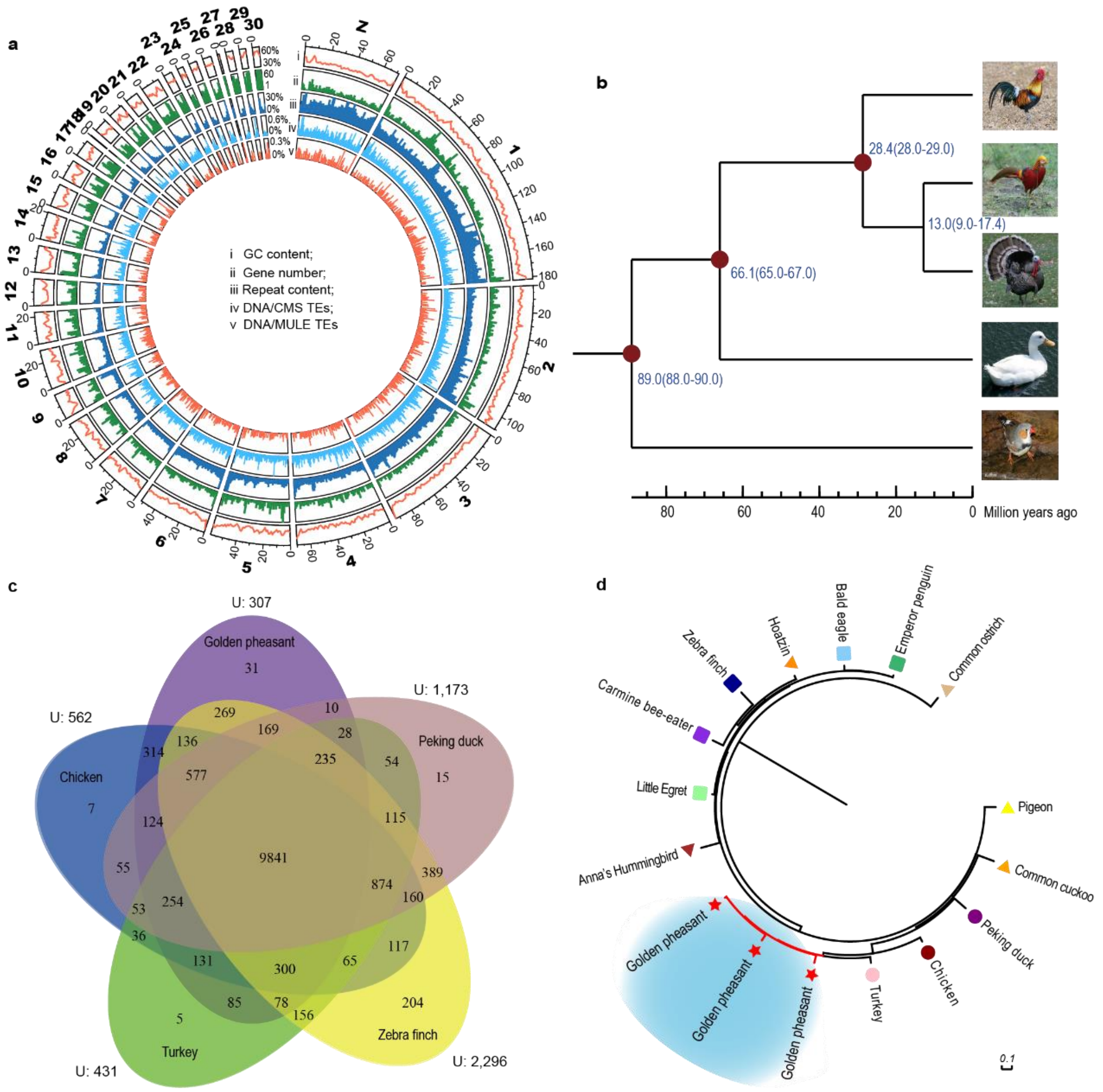

Fig. 2 Comparative genomic analysis among the golden pheasant and other avian species. (a) Global view of the golden pheasant genome using the pseudochromosomes. **(b)** The Maximum Likelihood phylogenetic relationships of the golden pheasant in Galloanseres. The tree was constructed based on 1.49 Mb 4-fold degenerate sites, from 8,079 single-copy orthologous genes among four sequenced Galloanseres genomes (golden pheasant, chicken, turkey, and duck) and the zebra finch (as outgroup). **(c)** Venn diagram of the shared orthologous groups among the five species. **(d)** Two more copies of CYP2D gene in golden pheasant. The background species are selected based on Jarvis's phylogeny for the 48 avian genomes, of which three Galloanseres birds and other ten birds from ten different clades are selected in this analysis.

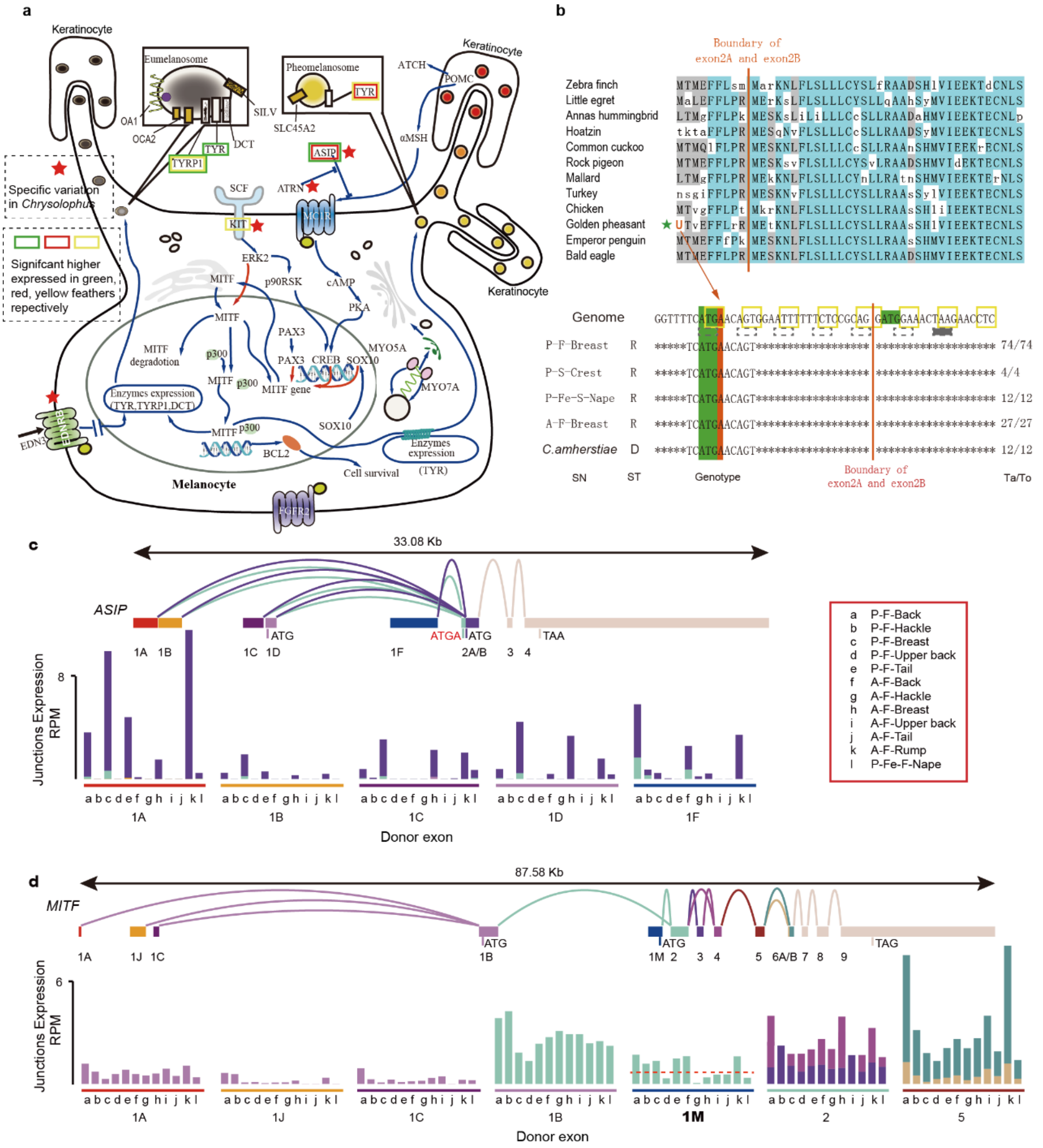

Fig. 3 The variation and alternative splicing of some regulator genes in the eu-/pheomelanin synthesis metabolism. (a) The pathway of Eu-/pheomelanin synthesis metabolism. The lineage specific variation genes in *Chrysolophus* are marked by red star. The significant higher expressed genes in feathers with green, red, and yellow color, are marked by the colorful rectangular respectively, all use the white feathers (A-F-Hackle and A-F-Breast) as control. **(b)** The single nucleotide insertion in the ASIP gene of the *Chrysolophus*. A base of adenine inserts after the initiation codon of the ORF at exon 2A. This insertion was verified in another five *Chrysolophus* individuals (lower

part). “SN”, sample name; “ST”, sequencing type, R, RNA sequencing, D, DNA sequencing; “Ta/To”, the number of reads support the shown genotype/the number of total mapped reads. **(c)** The RNA alternative splicing of ASIP gene. The upper section is the alternative splicing models of ASIP. Rectangles represent exons, and curves represent junctions between the exons. The size scale ratio between exons and introns is 1:10. The lower section is expression histogram of the junctions. RPM (Reads per million mapped reads) was used to normalize expression levels. The color of the column matches the accepter exon color. The color of the footstone matches the donor exon color. **(d)** The RNA alternative splicing of MITF gene. Descriptions are same with Fig. 3c. The description of sample name: “P-”, golden pheasant; “A-”, Lady Amherst’s pheasant; “-F-”, feather; “-S-”, skin; “-Fe-F”, feather from female; “-**”, body part.

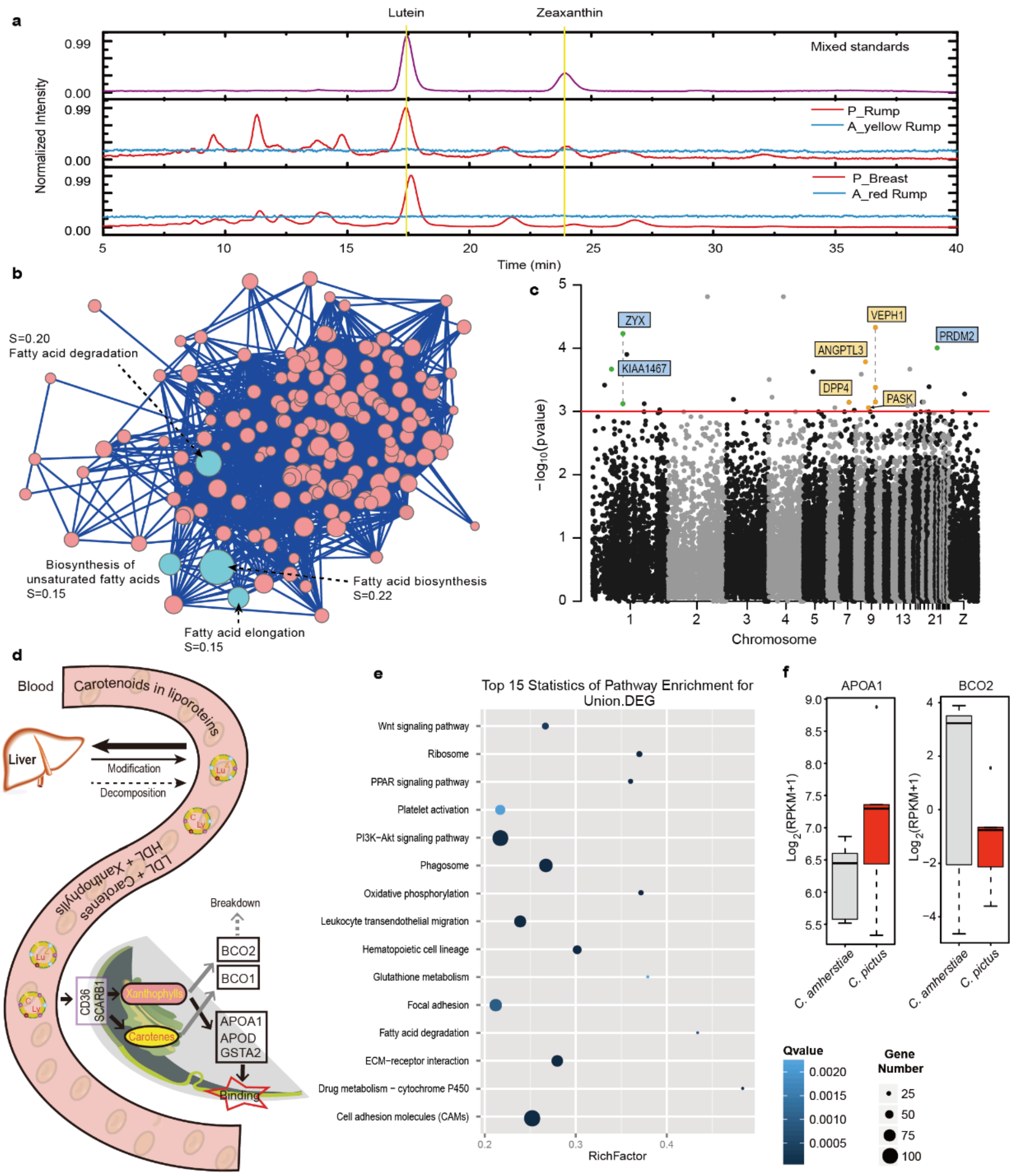

Fig.4 The comparative analysis and RNA expression of the carotenoid accumulation in feather. (a) The high performance liquid chromatography (HPLC) analysis of lutein and zeaxanthin in *Chrysolophus* red and yellow feathers. **(b)** The KEGG pathway annotation of the genes which is lineage-specific in golden pheasant but same in other non-feather-carotenoid 40 birds. The scoring standard of each pathway is described in the method. **(c)** The orthologous genes wide association study to the carotenoids accumulation. The coordinates are based on chicken chromosomes. Dashed line indicates the gene has more than one sites with P < 0.001. The green spots

are genes that also contain recent varied sites. The orange spots are lipid related genes. **(d)** The theoretical process of carotenoids transportation and deposition. **(e)** The KEGG pathway enrichment of the union DEGs between feather follicles of the two pheasants. The “RichFactor” = the number of DEGs in this pathway/the number of gene set in this pathway. More details are described in Additional file 3: Notes 4.2. **(f)** The expression of the APOA1 and BCO2 gene in the two pheasants.

Click here to access/download
Supplementary Material
Additional file 1 Figures.docx

Click here to access/download
Supplementary Material
Additional file 2 Tables.xlsx

Click here to access/download
Supplementary Material
Additional file 3 Notes.docx
