# Supplementary material for: Comparative genomics and transcriptomics of Chrysolophus provide insights into the evolution of complex plumage coloration
Source: Gigascience. 2018 Sep 6;7(10):giy113. doi: 10.1093/gigascience/giy113 (PMC6204425; doi:10.1093/gigascience/giy113)
Supplement: GIGA-D-18-00007_Revision_1.pdf [file giy113_giga-d-18-00007_revision_1.pdf]

GigaScience

Comparative genomics and transcriptomics of Chrysolophus provide insights into the evolution of complex plumage coloration
--Manuscript Draft--

**Manuscript Number:**	GIGA-D-18-00007R1
**Full Title:**	Comparative genomics and transcriptomics of Chrysolophus provide insights into the evolution of complex plumage coloration
**Article Type:**	Research
**Funding Information:**	State Key Development Program for Basic Research of China, 973 Program (2012CB22306) — Prof. Guangpeng Li<br>he Open Project of Key Development Program for Basic Research of Inner Mongolia Autonomous Region, National Natural Science Foundation of China (30960244) — Prof. Guangpeng Li<br>State Key Laboratory of Agricultural Genomics (2011DQ782025) — Dr. Chi Zhang
**Abstract:**	Background: As one of the most recognizable characteristics in birds, plumage color has a high impact on understanding evolution and mechanisms of coloration. Feather and skin are ideal tissues to explore the genomics and complexity of color patterns in vertebrates. Both two species of the genus Chrysolophus, golden pheasant (Chrysolophus pictus) and Lady Amherst's pheasant (Chrysolophus amherstiae), exhibit brilliant colors in their plumage, but with extremely phenotypic differences. This makes the two species can be of great models to investigate plumage coloration mechanisms in birds.<br>Results: We sequence and assemble a genome of golden pheasant with high-coverage and annotate 15,552 protein-coding genes. The genome of Lady Amherst's pheasant was sequenced with low-coverage. Based on the feather pigments identification, a series of genomic and transcriptomic comparisons are conducted to investigate the complex features of plumage coloration. Through identifying the lineage-specific sequence variations in Chrysolophus and golden pheasant, against different background, we find that four melanogenesis biosynthesis genes and lipid related genes may be candidate genomic factors for the evolution of their melanin and carotenoid pigmentation, respectively. In addition, a whole orthologous genes wide association study among 47 birds shows some candidate genes related to carotenoid coloration in a broad range of birds. The transcriptome data further reveal some important regulators of the two colorations, especially the MITF-1M splicing for the pheomelanin synthesis.<br>Conclusions: Analysis of the golden pheasant and its sister pheasant genomes, as well as comparing with other avian genomes, are helpful to reveal the underlying regulation of their plumage coloration. This study provides important genomic information and insights for further study of avian plumage evolution and diversity.
**Corresponding Author:**	Meng Xu<br>BGI<br>Shenzhen, Guangdong CHINA
**Corresponding Author Secondary Information:**	
**Corresponding Author's Institution:**	BGI
**Corresponding Author's Secondary Institution:**	
**First Author:**	Meng Xu
**First Author Secondary Information:**	
**Order of Authors:**	Meng Xu

Powered by Editorial Manager® and ProduXion Manager® from Aries Systems Corporation

	Guangqi Gao
	Yongchun Zuo
	Yulan Yang
	Chunling Bai
	Junyang Xu
	Zhuying Wei
	Jiumeng Min
	Guanghua Su
	Xianqiang Zhou
	Jun Guo
	Yu Hao
	Guiping Zhang
	Xukui Yang
	Xiaomin Xu
	Randall B Widelitz
	Cheng-Ming Chuong
	Chi Zhang
	Jun Yin
	Guangpeng Li
**Order of Authors Secondary Information:**	
**Response to Reviewers:**	Dear editor,<br>Thank you so much for giving us the opportunity to submit a revised version of our manuscript (The manuscript ID is: GIGA-D-18-00007). Frankly, we wanted to apologize for the delay in submitting the manuscript back to you, as the analyses and rewriting have taken too much time. Now we have carefully considered your feedback and submit this revised manuscript, in which the points from both reviewers are fully addressed.<br>For your point of comparisons to other recently completed (Phasianidae) genomes, we failed to use the five birds you advised because of no released assembly (willow grouse, rock ptarmigan, and gunnison sage grouse), poor quality assembly (black grouse, scaffold N50 was only 1,029 bp and maximum scaffold was only 11,073 bp), or the URL does not exist (prairie chicken, https://dovetailgenomics.com/wp-content/uploads/2015/12/Dovetail-Case-Study-Prarie-Chicken). We have supplemented three sequenced Galliformes species instead (Japanese quail, northern bobwhite, and scaled quail) to our comparative analyses to update the genomic comparison.<br>For the comments from Reviewer #1, we have performed expanded studies by adding three other Galliformes genomes in analyses of phylogenetic relationship and specific varied genes in golden pheasant. The study on golden pheasant CYP2J19 gene were supplemented either. For the comments from Reviewer #2, β-keratin subfamilies were re-annotated and analyses of their expressions and proportions were carried out. Besides, journal style was conformed, and DOI link of GigaDB dataset were cited in the "data availability. In the end, we accepted the reviewer's suggestion to improve the style of written English. The language of our revised manuscript has also been edited by American Journal Experts (Order ID J9ZTP83V).<br>Responses to each of the Reviewers' comments are listed below.<br><br>Thank you for your reconsideration.<br><br>Sincerely yours,<br>Meng Xu (On behalf of all the authors)<br>BGI Genomics, BGI-Shenzhen, Shenzhen 518083, China

Powered by Editorial Manager® and ProduXion Manager® from Aries Systems Corporation

Email: xumeng@bgi.com

Reviewer reports:

Reviewer #1: This manuscript used genomics, transcriptomics, spectroscopy and chromatography to investigate the genetic basis for complex coloration in the plumage of Chrysolophus pheasants. Given their striking differences in coloration but close sister relationship, they seem to be an excellent model for understanding pathways leading to divergence of coloration in birds. I do not have any major concerns about this manuscript, but I do have a few suggestions for how to improve it:

General comment 1: Probably the most important issue is that this needs to undergo more thorough editing of its English. It is largely understandable as is, albeit with some confusing passages, but it would benefit from being checked by a fluent English-speaker.
Response: Thanks for your suggestion and sorry for our un-native and poor English. We have revised manuscript carefully. The language of our revised manuscript has also been edited by American Journal Experts (Order ID J9ZTP83V).

General comment 2: I think this manuscript would benefit greatly from some expansion in the discussion regarding the evolution of coloration in birds. The manuscript currently reads as if it is simply reporting results without diving into how these results relate to the broader issue of the evolution of complex coloration in birds. Here we have an excellent example of closely related species with starkly contrasting coloration patterns, with some possible evidence of differences in gene expression and mutations, but not much is made of this, nor or is it related to the broader literature. Has anyone else looked at gene expression differences related to coloration in birds in a genome-wide scale? What about candidate gene approaches such as that of CYP2J19? Are there any overlaps with gene-specific studies? And more broadly, what might this mean about how the evolution of the same coloration can proceed via similar or divergent pathways? This might help draw more readers to your system and make an effort to dive into these questions in more detail. Of course, do not try to stretch your conclusions more than the data allow, and there is no need to review the entire literature, but I think there should certainly be more extensive exploration of what your data mean in the bigger picture.
Response: Thank you for your recognition of our work and giving us most valuable and helpful advices. Previous investigations on bird coloration in a genome-wide scale include crow and ruff genomes, which are based on Population Genomics (Poelstra JW et al. 2014, Science; Lamichhaney S et al. 2016, Nature Genetics; Küpper C et al. 2016, Nature Genetics). These studies identified fragment recombination in the avian genome whose melanin-based colorations are different. For the studies of coat-color related genes, most of the publications explored the polymorphism of single gene involved in melanogenesis, such as Mc1R (Page 3 line 16). Different expressions of ASIP and MITF were investigated mainly in mammals. It may be because there were less avian genomes published by the end of 2014. Therefore, reports on the mechanism of bird coloration at the genomic level are still limited. On the other hand, vertebrate carotenoid-based coloration is specific to avian, compared to mammals. And the utilization of this diet-derived pigment is hard to investigate at gene level. So it is lack of literature about genetic mechanisms on carotenoid-based coloration in avian species. Our current study is based on de novo sequencing of a single individual, which could provide a reference assembly for the next work of population genomes re-sequencing or the transcriptome sequencing in the future. Besides, we focused on both melanin-based and carotenoid-based colors in golden pheasant, because this species is one of the few Galliformes to express carotenoids in the feather follicles. We have added some literatures in the revised manuscript for the ASIP and MITF genes (Page 11 line 2; Page 11 line 17). This study identified a mechanism involving relatively few elements that can regulate the selective deposition of carotenoids, eumelanin/pheomelanin to produce the vast array of color patterns in avian plumage. This study also provides a platform for explaining how the complex patterns and shades of avian plumage colors are produced by differential and combinatorial use of this multi-layered coloring mechanism involving melanins, carotenoids and β-keratins. It could be proposed that extraordinarily complex plumage patterns are not only encoded by the genome but also produced by the mechanisms underlying multi-layered plumage coloring (Page 17 line 7; revised Additional file 1: Figure S16). We

Powered by Editorial Manager® and ProduXion Manager® from Aries Systems Corporation

believe these data are conducive to more extensive and systemically explorations on golden pheasant or even on multiple avian species at the genome level in the future. Analyses of CYP2J19 and specific genes in golden pheasant are supplemented in this revised manuscript as you advised. Details of our responses are described below.
Page 7, lines 6-8: while I appreciate the effort to reconstruct the phylogeny of Chrysolophus relative to Meleagris and Gallus, this seems to be uncontroversial with other studies having supported the Chrysolophus+Meleagris relationship with far better taxon sampling (e.g., Wang et al. 2013 PLoS One). Even though the authors here use many more loci to resolve this relationship, it seems to simply recapitulate a result that was already pretty clear, and it is ultimately not relevant to the focus of this study.
Response: Thanks for your suggestion. We have accepted your advice by adding more species to the comparative genome analyses. According to the species mentioned in the publication (Wang et al. 2013 PLoS One) and genome available avian, we have reconstruct the phylogeny using six Galliformes (golden pheasant, chicken, turkey, Japanese quail, northern bobwhite and scaled quail), the sequenced Anseriformes (duck) which is closest to Galliformes, and a model species (zebra finch) as an outgroup (Page 7 line 4; Figure 2b).

Page 8, lines 15-17: note that the two studies on CYP2J19 only looked at passerines, whereas Emerling 2018 (Molecular Phylogenetics and Evolution) provided evidence that this gene is responsible for the production of red carotenoids more broadly across Aves. I think this is interesting and significant for the broader question of coloration in birds (see General Comment 2 above). For example, why did the Chrysolophus species not co-opt CYP2J19 to create their striking red coloration? This of course is not testable in the current study, and perhaps merely speculative, but I think this is relevant to the broader question of how and why birds convergently evolved similar coloration but often via different mechanisms.
Response: We accepted the reviewer's suggestion. CYP2J19 were comparatively analyzed within the eight avian species mentioned above. We download the protein sequences of CYP2J19(A/B) and CYP2J40 provided by Mundy (Mundy NI et al. 2016, Current Biology, doi:10.1016/j.cub.2016.04.047), and their un- and downstream genes (HOOK1 and NFIA), aligned to the compared genomes using the same pipeline. Through combining the syntenic relationship and alignment identity, we identified the CYP2J19 in the genomes. For copy number, there is only one copy of CYP2J19 in Galliformes and duck genome but two copies in zebra finch. For multiple sequence alignment, CYP2J19 protein sequences are similar in Galliformes. The golden pheasant sequence is more similar to the turkey. The expression of CYP2J19 in the orange nape of golden pheasant was significantly higher than other colored feathers in Chrysolophus. It suggested that CYP2J19 might play roles at transcriptional level in Chrysolophus. These results were supplemented in the last paragraph of "Evolution analysis within Galliformes" of the revised manuscript (Page 8 line 17).
On the other hand, we found the red color of golden pheasant were not red-carotenoid based coloration based on our pigment identification experiments. The extracted lipid pigments from red feathers exhibited visual yellow. The red golden pheasant belly feathers remained the initial color even after the thermochemical procedures, and the aqueous phase was still colored even after treatment with multiple extractions (Figure S17). The aqueous phase of nape orange feathers was colorless after extractions, and the once-pigmented tissues nearly lost their color entirely. It can be indicated that different carotenoids are present in different feathers in golden pheasant (Details are described in Additional file 3: Supplementary Notes 1.2). The different expression of CYP2J19 in golden pheasant feathers may affect the utilization of carotenoids. But the influence of sequence evolution remains unclear for golden pheasant. Many birds have CYP2J19 gene, but can't accumulate carotenoid in feather, such as the other Galliformes in this study, and zebra finch although its beak can accumulate carotenoid. The independent evolution for feather carotenoid in several birds indicate this ability may be controlled or impacted by multiple genes.

Page 9, line 12: you discuss lineage specific "varied" genes in Chrysolophus. First of all, it is not clear what is meant by varied genes and the significance of this, but I assume this is getting at genetic synapomorphies (substitutions) or something of that nature. First of all, I'm unsure if such a metric is relevant because it is not clear if you're referring to all substitutions/variations, or simply those that are nonsynynomous, which are usually what researchers discuss. Regardless, you refer to them as being lineage specific and discuss it in the context of Chrysolophus. It is important, however, to note

Powered by Editorial Manager® and ProduXion Manager® from Aries Systems Corporation

that any substitutions present in Chrysolophus may also be shared by at least another six genera (Wang et al. 2013). So any significance gleaned from these analyses should be tempered by the understanding that they might not be specific to Chrysolophus only.
Response: We accepted the reviewer's suggestion. We have re-written some sentences/words. We identified the lineage-specific varied genes based on the protein sequences, which mean the variations were nonsynynomous. According to the the publication (Wang et al. 2013 PLoS One), most mentioned Phasianidae species are not genome available. Therefore, we have added three sequenced Galliformes (Japanese quail, northern bobwhite, and scaled quail) to our genomic comparative analyses. In this way, the lineage-specific varied gene set could be gleaned to a certain extent (Page 9 line 21). Actually, except the Galliformes, we also used 11 other birds as background. These background birds should be enough to identify the conserved genotype among the bird evolution.

Reviewer #2: The manuscript entitled "Comparative genomics and transcriptomics of Chrysolophus provide insights into the evolution of complex plumage coloration" sequenced two pheasant genomes and performed comparative genomics and transcriptomics to identify variations in plumage related genes. Overall, this is a significant and thorough study and should be published. However, the grammar and word choice is poor and the writing style is awkward and needs significant work. In this review, I did not spend time trying to correct these issues. I do, however, point out instances where the poor writing effects the understanding of the results.
Response: Thank you for your recognition of our work and giving us most valuable and helpful advices. We accepted the reviewer's suggestion to improve the style of written English. The language of our revised manuscript has also been edited by American Journal Experts (Order ID J9ZTP83V).

Results and Discussion
Page 8, lines 3-4 "For the multi-copy families……" sentence. Do the authors mean gene families? Are the 31 lineage specific gene families simply being identified by number or are members of the gene families showing evidence of positive selection? Please clarify the meaning of this sentence.
Response: Yes, it means multi-copy gene families which were identified in golden pheasant by hierarchical clustering. This sentence has been clarified (Page 8 line 2). The gene family was regarded as lineage specific if its containing genes were only from one species. The positive selective analysis was based on the one-to-one orthologous genes.

Page 14, While I think it is interesting that they analyzed the relationship of β-keratin copy number and sequencing depth, I think they should also focus on identifying the β-keratin subfamily (feather, claw, keratinocyte, etc.) membership of the genes that were found to be differentially expressed. Also, despite the underrepresentation of β-keratins in sequenced genomes, they should still be able to identify the relative proportions of the β-keratin subfamilies in the pheasant genomes compared to other avian species.
Response: Thanks for your valuable suggestion. The β-keratins were re-annotated to subfamily based on the best hit to the β-keratins of zebra finch according to previous studies (Greenwold MJ and Sawyer RH, J Exp Zool B Mol Dev Evol, 2013). Then we updated the analyses of β-keratins and performed a phylogenetic analysis of β-keratins in six Galliformes (Page 14 line 23, Page 15 line 4).

Methods
The authors need to specify the identity of the tissue used for the comparative transcriptome work. I was only able to find references to the color of the feathers. Were these adult or juvenile feathers? Taken during a molt etc.? Which part of the feather was tissue taken?
Response: Acknowledged and accepted your suggestion. For the feather follicles sampling, we used the previously published method (Poelstra JW et al. 2014, Science), briefly, we plucked their feathers during natural molting, then allowed feathers to grow for two-four weeks, at which stage the previously plucked areas of skin contained densely spaced feather shafts with the first parts of feathers about to or just protruding from the shafts. These methods are present in Additional file 3: Supplementary Notes 2.2.

	Figure 2 legends.<br>(c) is the Venn diagram of gene families? The legend and main text are confusing.<br>(d) What are the "clades" of these species and why were those specific species chosen in those clades? Better genome builds? Please specify.<br>Response: Thank you for your suggestion.<br>(c) Sorry for the confused writing and we have uniformed the writing (Page 8 line 2, Page 34 line 1).<br>(d) Yes, the selected species in each clade because of their better assembly, we have supplemented the description in the Method part (Page 9 line 21, Page 34 line 4).
**Additional Information:**	
**Question**	**Response**
Are you submitting this manuscript to a special series or article collection?	No
**Experimental design and statistics**<br><br>Full details of the experimental design and statistical methods used should be given in the Methods section, as detailed in our Minimum Standards Reporting Checklist. Information essential to interpreting the data presented should be made available in the figure legends.<br><br>Have you included all the information requested in your manuscript?	Yes
**Resources**<br><br>A description of all resources used, including antibodies, cell lines, animals and software tools, with enough information to allow them to be uniquely identified, should be included in the Methods section. Authors are strongly encouraged to cite Research Resource Identifiers (RRIDs) for antibodies, model organisms and tools, where possible.<br><br>Have you included the information requested as detailed in our Minimum Standards Reporting Checklist?	Yes
**Availability of data and materials**<br><br>All datasets and code on which the	Yes

Powered by Editorial Manager® and ProduXion Manager® from Aries Systems Corporation

conclusions of the paper rely must be either included in your submission or deposited in publicly available repositories (where available and ethically appropriate), referencing such data using a unique identifier in the references and in the “Availability of Data and Materials” section of your manuscript.<br><br>Have you have met the above requirement as detected in our Minimum Standards Reporting Checklist?	

Powered by Editorial Manager® and ProduXion Manager® from Aries Systems Corporation

Manuscript
Click here to view linked References
Click here to access/download;Manuscript;revised manuscript-0630.docx

Comparative genomics and transcriptomics of *Chrysolophus* provide insights into the evolution of complex plumage colouration

Guangqi Gao[1,2†], Meng Xu[3†], Yongchun Zuo[1,2†], Yulan Yang[3†], Chunling Bai[1,2†], Junyang Xu[3†], Zhuying Wei[1,2], Jiumeng Min[3], Guanghua Su[1,2], Xianqiang Zhou[3], Jun Guo[4], Yu Hao[4], Guiping Zhang[3], Xukui Yang[3], Xiaomin Xu[3], Randall B Widelitz[5], Cheng-Ming Chuong[5], Chi Zhang[3*], Jun Yin[4*], Guangpeng Li[1,2*]

[1]The State key Laboratory of Reproductive Regulation and Breeding of Grassland Livestock, Inner Mongolia University, Hohhot, 010070, China.

[2]College of Life Science, Inner Mongolia University, Hohhot, 010070, China.

[3]BGI Genomics, BGI-Shenzhen, Shenzhen 518083, China

[4]College of Life Science, Inner Mongolia Agricultural University, Hohhot, 010018, China.

[5]Department of Pathology, Keck School of Medicine, University of Southern California, Los Angeles, CA 90033, USA.

[†]Co-first author

[*]Correspondence: gpengli@imu.edu.cn, zhangchi2@genomics.cn, yinjun@imau.edu.cn.

Abstract

Background: As one of the most recognizable characteristics in birds, plumage colour has a high impact on understanding the evolution and mechanisms of colouration. Feather and skin are ideal tissues to explore the genomics and complexity of colour patterns in vertebrates. Two species of the genus *Chrysolophus*, golden pheasant (*Chrysolophus pictus*) and Lady Amherst's pheasant (*Chrysolophus amherstiae*), exhibit brilliant colours in their plumage, but with extreme phenotypic

differences, making these two species great models to investigate plumage colouration mechanisms in birds.

Results: We sequence and assemble a genome of golden pheasant with high-coverage and annotate 15,552 protein-coding genes. The genome of Lady Amherst's pheasant is sequenced with low coverage. Based on the feather pigment identification, a series of genomic and transcriptomic comparisons are conducted to investigate the complex features of plumage colouration. Through identifying the lineage-specific sequence variations in *Chrysolophus* and golden pheasant, against different backgrounds, we find that four melanogenesis biosynthesis genes and some lipid-related genes might be candidate genomic factors for the evolution of melanin and carotenoid pigmentation, respectively. In addition, a study among 47 birds shows some candidate genes related to carotenoid colouration in a broad range of birds. The transcriptome data further reveal important regulators of the two colourations, particularly one splicing transcript of the microphthalmia-associated transcription factor (MITF) gene for pheomelanin synthesis.

Conclusions: Analysis of the golden pheasant and its sister pheasant genomes, as well as comparison with other avian genomes, are helpful to reveal the underlying regulation of their plumage colouration. The present study provides important genomic information and insights for further studies of avian plumage evolution and diversity.

Keywords: genome, transcriptome, *Chrysolophus*, plumage, colouration

Background

The plumage colours of birds serve functions in crypsis, social signalling and mate choice [1]. Due to the diversity of colours and ease of observation, plumage provides

an ideal model to explore the formation and genomic evolution of colouration patterns in animals. Studies on birds and mammals suggested that the integument colours are regulated by several mechanisms. Melanin, which is produced by neural crest cell-derived melanocytes, is a major contributor to pigmentation in avian feathers and mammalian hairs [2]. Black and brown feathers are derived from the deposition of eumelanin, whereas reddish and light-yellow feathers are due to pheomelanin. Carotenoids are chemicals for vitamin synthesis and act as antioxidants for the immune system [2]. Some birds can use dietary-derived carotenoids to produce yellow, orange and red in their feathers, such as lutein, zeaxanthin, β-cryptoxanthin, and β-carotene [3]. Red colours may also come from other rare pigments, such as porphyrins in black-shouldered kites [4], psittacofulvins in parrots [5], iron oxide in *Gypaetus barbatus* and turacin in *Tauraco macrorhynchus* [6]. In addition, feather colouration may also be a result of specific structures that combine with non-iridescent colours and iridescent metal lustres [2].

Feather complex colouration is likely coordinated through multiple genes that regulate diverse mechanisms. The melanogenesis biosynthetic pathway has been elucidated [7, 8], and previous studies have revealed the DNA polymorphisms of several genes that lead to variations in melanin-based colouration [9]. However, some details regulating the switch of eu-/pheomelanin remain unresolved [10]. Some candidate genes for carotenoid-related functions in mammals and invertebrates have been documented, and their homologous genes may also present in birds [11]. However, the production metabolism of carotenoid pigments has not been well characterized. Additionally, the nanostructural colours of feathers are related to keratinization and affected by keratin genes [12, 13]. In birds, keratins belong to a large family with nearly 200 members [14], whose functions should be investigated.

Genome information could provide new perspectives to study the mechanisms of bird colouration. In 2014, the most extensive comparative analysis of avian species at the genome level to date was published, revealing two genes with a negative correlation between colour discriminability and *dN/dS* across birds [15, 16]. However, this work included only 15 genes without distinguishing melanin, carotenoid, or other pigments. Thus, further studies are necessary to investigate the candidate molecular mechanisms of avian plumage colouration.

In the present study, we focused on the plumage colouration issues of golden pheasant (*Chrysolophus pictus*) at the genome and transcriptome levels, and together with its sister species, the Lady Amherst's pheasant (*Chrysolophus amherstiae*). These species are two important organisms for studies of plumage colouration because of their phenotypic differences and close relationship. These two species can even cross-breed to produce fertile offspring under human feeding conditions. In adult male golden pheasant, the crest and rump feathers are both golden-yellow in colour, the belly and upper tail coverts are dark red, the nape feathers are light orange with two black stripes, the mantle is iridescent green, and the tail is black spotted with cinnamon (Figure 1, Figure S1). The golden pheasant is a colourful avian species with distinct brilliant feather colours in adult males, which can be observed with obvious characteristics of melanin and carotenoid pigments. By comparison, adult male Lady Amherst's pheasants have red and yellow feathers exclusively distributed in small parts of the body, including the crest, rump and upper tail coverts, while most of the other body parts are white or black (Figure 1, Figure S1). Carotenoids were present in the yellow back feathers of golden pheasant but it was unclear whether they were present in Lady Amherst's pheasant [17]. In the present study, we sequenced the genome and transcriptome of these two pheasants and identified the melanin and

carotenoid pigments in plumages of the two pheasant species by using high-performance liquid chromatography (HPLC) and Raman spectroscopy (RS) methods. Then, we conducted a comprehensive comparative analysis with 51 other sequenced avian references [15, 18, 19] at a suitable level to investigate the evolution of the plumage colouring of golden pheasant or *Chrysolophus*.

Results and Discussion

Genome assembly and annotation

The genomic DNA of golden pheasant was extracted by using blood genomic DNA from a male adult from Foping National Nature Reserve in Shaanxi and fed in Jilin, China. A series of paired-end libraries with different insert sizes were constructed and sequenced by using the Illumina Hiseq 2000 platform (Table S1). The *de novo* assembly size was 1.029 Gb, with a contig N50 size of 34.4 kb and a scaffold N50 size of 1.55 Mb (Table 1). Assembly quality was assessed by aligning the total small insert size reads (170 ~ 800 bp) to the assembly. These reads covered 99.92% of the genome, and 99.17% of the alignment could be mapped by more than 10 reads (Table S2; Figure S2). In addition, the assembly covered more than 95.71% of the transcriptome-assembled transcripts (102,426 out of 107,012, Table S3), indicating the high quality of the golden pheasant assembly.

To obtain a global view of potential specific elements in golden pheasant, 93.9% of the assembly was linked to pseudo-chromosomes by using turkey chromosomes as a reference (Figure 2a). The genomic DNA from a male Lady Amherst's pheasant was sequenced with relatively low coverage (approximately 43×). We identified 7.26 million SNPs and 0.45 million InDels (1-5 bp per InDel, total 0.83 Mb length) (Table S4) in Lady Amherst's pheasant by using the assembly of golden pheasant as a reference, indicating that the divergence between these two pheasants was

approximately 0.84%. Moreover, the golden pheasant genome was used as a reference to align the transcriptome sequences from these two species. The average mapping rates of golden pheasant and Lady Amherst's pheasant are 85.82% and 81.83%, respectively. These results imply a close relationship between these two species.

Combining the homology-based and transcriptome-assisted methods, 15,552 protein-coding genes were identified in the assembly of golden pheasant, of which 98.69% of the genes were homologous to public databases (SwissProt, Nr, and KEGG) (Table S5), and 89.43% of the genes were supported by transcriptome sequences (RPKM > 1 in at least one sample). Moreover, repetitive elements (REs) comprised approximately 10.93% of the golden pheasant genome, with the chicken repeat 1 (CR1) elements being the most abundant class (83.14% of REs; 0.093 Gb), which was similar to that for chicken (Table S6). The expanded satellite DNAs in the golden pheasant genome were 5.5- and 18.2-fold that of the chicken and zebra finch genome, respectively (Figure S3; Table S7). There were no lineage-specific REs identified in golden pheasant, but a similar evolutionary trend of DNA/CMC and DNA/MULE transposable elements (TEs) were found between the golden pheasant and turkey (Table S7). Increasing evidence has suggested that TEs might play a role as candidate gene expression regulators, especially in the modulation of abutting gene expression [20-22]. Thus, genes within 2 kb up- and downstream of these TEs were examined. The flanking genes of the satellite DNAs, CMC, and MULE could be enriched in sodium-potassium exchange ATPase activity (GO: 0005391, Adjust *P*-value = 0.02295), cell development (GO: 0048468, Adjust *P*-value = 1.93E-08), and kidney development (GO: 0001822, Adjust *P*-value = 0.00128), respectively (Figure S4). Functional enrichment showed that the specific or expanded REs may be involved in the adaptive evolution of golden pheasant or turkey.

Evolution analysis within Galliformes

The phylogenetic placement is a critical background for many comparative genomic analyses. To assess the phylogenetic position of the golden pheasant in Galliformes, a phylogenetic tree was constructed with five other sequenced Galliformes (chicken [23], turkey [24], Japanese quail [18], northern bobwhite and scaled quail [19]), the sequenced Anseriformes (duck [25]) which is closest to Galliformes, and a model species (zebra finch [26]) as an outgroup. The phylogenetic analysis of 48 avian concluded that protein-coding genes might reflect life history traits more than phylogeny topology would [16]. Therefore, we constructed the phylogeny tree using 996,755 4-fold degenerate sites (4D sites, from 6,538 one-to-one orthologous genes) that do not affect the amino acids coding and are typically considered to be less subject to selective pressure. The result showed that the golden pheasant is taxonomically closer to turkey than to chicken (Figure 2b, Figure S5). The relationship was consistent with the above REs analysis that golden pheasant and turkey had the similar divergence distribution (Figure S3) and shared some common specific REs, which belong to non-coding regions (Table S7). This phylogeny was also uncontroversial with a previous study which was based on six nuclear intron sequences and two mitochondrial regions [27]. Furthermore, the divergence time of the golden pheasant and turkey was estimated approximately 13 million years ago by using MCMCTree (Figure 2b).

Sequence divergences and/or gene duplications have been proposed as important mechanisms in the course of evolution [28]. Identifying these variations may provide clues for the next investigations. Positive Darwinian selection is a universal strategy to identify candidates of adaptive evolution at the DNA sequence level. For the 6,538

one-to-one orthologous genes in eight birds, 676 positive selected genes were identified in golden pheasant by using branch site model (Table S8 and S9). For the multi-copy gene families, 241 lineage specific gene families were identified in golden pheasant (Figure 2c) by hierarchical clustering. Additionally, we identified 132 expanded and 18 contracted gene families through a maximum likelihood framework (Table S10 and S11). It is noteworthy that cytochrome P450 family 2 subfamily D member 6 (*CYP*2*D*6) was duplicated to three copies in golden pheasant genome (Figure 2d, Figure S6), which was just one copy in all the 48 birds [16]. Although this gene is multiple copies in northern bobwhite or scaled quail, it should be independently events for the duplication in the two Odontophoridae species and golden pheasant respectively based on the phylogeny (Figure 2d). The CYP enzymes were considered as good candidates for carotenoid ketolases [29]. Recently, a comparative analysis among 65 bird genomes revealed the *CYP*2*J*19 gene, which belonged to the same clan as that of *CYP*2*D*, was a carotenoid ketolase functional in synthesizing red carotenoids from yellow carotenoids [30], and other two population studies revealed the *CYP*2*J*19 was associated with red carotenoid-based colouration phenotypes in zebra finches and canaries [29, 31]. Compared with other non-carotenoid Galliformes, copy number and protein sequence of *CYP*2*J*19 in golden pheasant unchanged (the sequence is more similar to the turkey). However, the expression of *CYP*2*J*19 in the orange nape of golden pheasant was significantly higher than other coloured feathers. It could be suggested that *CYP*2*J*19 might play roles at transcriptional level in *Chrysolophus*. Additionally, *CYP*2*D*6 had the maximum allelic polymorphism among the CYP family in humans [32], and was responsible for approximately 25% of the metabolism of known drugs [33], indicating the wide range functions of *CYP*2*D* gene. The expanded *CYP*2*D*6 genes in golden

pheasant may benefit the metabolism or biotransformation of some foreign chemicals and could be a candidate evolution factor for carotenoid deposition in its feathers.

Lineage-specific variations and alternative splicing of melanin genes in *Chrysolophus*

Melanin is the most common and widespread pigment in avian feathers and yields black, grey, brown, rufous and buff shades and patterns [2]. Both *Chrysolophus* species possessed darker eumelanic and brighter pheomelanic colours in their integument plumage, particularly the most impressive bright red and yellow feathers in male individuals (Figure 1). A previous investigation concluded that human hairs with six different colours, varying from black to brown to red, all contained both eumelanin and pheomelanin but that their proportions determined the visual colours. The eumelanin content and proportions were the highest in black hairs, while red hairs contained comparable levels of eumelanin and pheomelanin [34]. The present HPLC results also showed that feathers with different colours from golden pheasant and Lady Amherst's pheasant varied according to the ratio of eu-/pheomelanin (Figure S7). This finding could indicate that the clear feather colours of the two pheasant species might result from the relatively extreme mixture ratio of eu-/pheomelanin. Based on this information, we focused on the genetic regulations of the eu-/pheomelanin switch in *Chrysolophus* birds from both genomic and transcriptomic perspectives.

We identified the lineage-specific varied genes in *Chrysolophus* by comparing with the five other Galliformes species and 11 more birds with high quality of genome build which belong to 11 different clades in the phylogeny tree of the 48 birds [16]. Four melanogenesis-associated genes have specific mutated sites in *Chrysolophus* species, including attractin (ATRN), endothelin receptor B (EDNRB), KIT proto-oncogene tyrosine-protein kinase (KIT), and agouti signalling protein (ASIP)

(Figure 3a). ATRN has at least 8 sites under positive selection, with >1 (BEB test [35], P > 0.98), which could prevent the formation of the "Kelch repeat type 1" domain (PF01344) based on the InterProScan annotation [36] (Figure S8). EDNRB has a three-amino acid deletion in the "G protein-coupled receptor, rhodopsin-like" domain (PF00001, Figure S9), and KIT has a two-amino acid deletion in the C-terminal region, which are conserved in other birds and even in green anole (Figure S10). In the *ASIP* gene, a single nucleotide is inserted after the initiation codon at exon 2A, which may impact 50% kind of ASIP isoforms by disabling this initiation codon or causing a frameshift resulting in a premature transcription termination at the 13th cordon (Figure 3b). Melanogenesis is under multiple levels of complex regulation, mainly through the transcriptional and post-transcriptional regulation of *MITF* gene, which can stimulate the transcription of genes that function in producing melanin [37-40]. The classic transcriptional regulator of *MITF* is the melanocortin-1 receptor (MC1R) with its ligands, alpha-melanocyte-stimulating hormone (α-MSH) and ASIP. ASIP can competitively antagonize α-MSH to bind MC1R, and ATRN is an obligatory accessory receptor for ASIP that enhances ASIP-Mc1R binding [37]. From another aspect, KIT can mediate the phosphorylation of MITF protein at Ser73 through the mitogen activated protein kinase (MAPK) pathway and trigger short-lived MITF activation as well as ubiquitin-dependent proteolysis [38, 39]. Moreover, EDNRB stimulation not only activates MITF expression but also elicits MAPK-mediated MITF phosphorylation [40]. As located in the upstream of melanogenesis pathway, variations of these four genes may amplify the biosynthesis or switches of eumelanin and pheomelanin through a signalling cascade [38, 39], resulting in a more extreme mixture ratio of eu-/pheomelanin in *Chrysolophus*.

Gene variations can alter plumage colour traits among different birds, however, the

diversity of colours and patterning present in one individual may be due to gene expression or alternative splicing [41]. Two promoters of the *ASIP* gene, the proximal hair cycle-specific promoter and the distal ventral-specific promoter, have been identified in mice and rabbits [42, 43]. Recent reports identified three conserved classes of ASIP mRNA variants that are specifically expressed in the dorsal and ventral feather follicles of chickens [44, 45]. We sequenced the RNA of feather follicles from different body parts in two pheasants and identified at least 10 *ASIP* mRNA isoforms generated by alternative splicing (Table S12), in which *ASIP*-1A isoforms are highly expressed in red-pheomelanin feathers, while *ASIP*-1F isoforms are abundant in yellow-pheomelanin feathers (Figure 3c). MITF, another central regulatory element of the melanogenesis pathway, regulates at least 11 melanogenesis genes directly or non-directly through feedback loops [46], and exhibits a complex alternative splicing pattern in *Chrysolophus* feather follicles. The *MITF* consists of at least 13 exons and two ORFs which are translated from exon-1B and exon-1M (Figure 3d; Table S13). We demonstrated herein that *MITF-M* isoforms are preferentially expressed in pheomelanin-containing feathers (Fold change=3.80, Adjust *P*-value=1.35E-11, Figure 3d). *MITF-M* has been thought to be specifically expressed by melanocytes, but its expression has been identified in the retinal pigment epithelium [47]. This result indicates that the *MITF*-1M isoform may be a key factor to regulate pheomelanin synthesis in the feather follicles of *Chrysolophus*.

Carotenoid utilization in *Chrysolophus* plumage

Carotenoids, a class of organic fat-soluble compound, are synthesized by plants, bacteria or fungi and utilized by animals through their diets [48]. Depending on the chemical structure, these pigments typically appear yellow, orange or red in avian plumage [2]. In the present study, both pheasant species have yellow to red plumage,

but carotenoids were only found in the golden pheasant. Raman spectroscopy (RS) [49] showed carotenoid bands in golden pheasant feathers but not in Lady Amherst's pheasant feathers (Figure S11a and b). Further identification by HPLC revealed that these carotenoids included lutein and zeaxanthin (Figure 4a, Figure S12, Table S14). The two other sequenced Galliformes, chicken and turkey, also do not accumulate carotenoids in feathers. It is likely that golden pheasant acquired this new ability. Thus, the variations after its speciation from the ancestral species, but conserved in non-feather-carotenoid birds, may contain the clues related to the new phenotype of feather carotenoids. In the 48 published avian genomes [15], four birds (rifleman, carmine bee-eater, white-tailed tropicbird and American flamingo) have been revealed the presence of carotenoids in their feathers, while 39 birds show the absence of carotenoids in a previous study by using HPLC and RS methods [17]. With the Lady Amherst's pheasant and 39 non-feather-carotenoid birds as background, we selected the lineage-specific non-synonymous variations in golden pheasant but conserved in the other 40 birds. Finally, we identified 258 genes containing such variations in golden pheasant (Table S15). KEGG pathway annotation revealed that the top four scored pathways belonged to "lipid metabolism" (Figure 4b). The lineage-specific varied genes also contain another lipid transport gene, apolipoprotein B (*APOB*) which is the main apolipoprotein of chylomicrons and low-density lipoproteins (LDL). The biological functions of lipids include the storage and transportation of fat-soluble vitamins, including carotenoids. The transportation of carotenes requires LDL, and the transportation of xanthophylls requires high-density lipoprotein (HDL) [2]. The evolution of those lipid-related genes may change the storage and transportation of carotenoids in golden pheasant, which may be related to the accumulation of carotenoids in its feathers.

In addition, the five feather-carotenoid birds are from five different clades (Passerimorphae, Coraciimorphae, Phaethontimorphae, Phoenicopterimorphae, and Galliformes), indicating that these birds may have independently acquired this ability. To detect whether some genes experience potential convergent variations in feather-carotenoid birds, we separated the birds into two groups, feather-carotenoid and non-feather-carotenoid, and then performed a whole orthologous gene-wide association study between the two groups. We identified 48 genes containing genotype (at amino acid level) that might be associated with the accumulation of carotenoids in feathers (hypergeometric test, $P < 0.001$, Table S16). One of these genes, *Zyxin* (*ZYX*), is present at cell-cell contact sites and shuttles to the nucleus, where it affects cell fate and growth [50]. ZYX participates in an interaction network with the gamma subfamily of peroxisome proliferator-activated receptor (PPAR-gamma) [51], which is a nuclear hormone receptor, and regulates adipocyte differentiation and lipid metabolism [52, 53]. The 48 genes also included four other lipid-associated genes and three genes that overlapped with the lineage-specific varied genes in golden pheasant (Figure 4b, 4c). These varied genes in golden pheasant and even in more carotenoid birds may be candidate factors of carotenoid deposition in avian plumage, especially lipid related genes. As a kind of lipochrome, carotenoids are circulated in the same way with lipids. They are packaged into chylomicron fractions *in vivo*, enter and transport in bloodstream, where they incorporated with lipoproteins, such as high density lipoprotein (HDL) and low density lipoprotein (LDL) [2] (Figure 4d). Thus, our results indicate that the phenotype of carotenoids deposition in feathers may be controlled or impacted by multiple genes and provide some candidate genes that may associate to this phenotype via a genome-wide comparison.

Transcriptome analysis showed that differentially expressed genes (DEGs) between golden pheasant (carotenoid contained) and Lady Amherst's pheasant (non-carotenoid contained) feathers were enriched in the PPAR signalling pathway (Figure 4e), which mediates the effects of fatty acids and their derivatives [52]. In this pathway, the apolipoprotein gene *APOA*1 was up-regulated in golden pheasant plumage (Figure 4f). In addition, another xanthophyll carotenoid cleavage enzyme gene, *BCO*2, was expressed at a low level in golden pheasant plumage (Figure 4f). APOA1 is the major protein component of HDL [54] , which is the predominant carrier of xanthophylls in plasma [2]. Given the presence of lutein and zeaxanthin, and the expression pattern of *APOA*1, APOA1 may be a carotenoid-binding protein (CBP) in golden pheasant feather follicles. BCO2 enzyme can cleave xanthophyll carotenoids at 9-10 or 9'-10' carbon-carbon double bonds [55]. A nonsense mutation or inefficiency of BCO2 results in the abnormal accumulation of carotenoids in livestock adipose tissue [56, 57], primate retina [58], chicken skin [59] and golden-winged warbler feathers [60]. Based on these results, we could hypothesize a process that after transportation into feather follicles, carotenoids bind to APOA1, while the expression of *BCO*2 affects carotenoid deposition (Figure 4d).

Connections of β-keratin in plumage colouration and genome quality

Beta-keratins are major components of plumage, and evolution of the β-keratin multigene family may contribute to the novel characters of feathers [14, 61]. In the present study, a total of 66 β-keratin genes were identified in the golden pheasant assembly, including 42 feather β-keratins, 9 scale β-keratins, 6 claw β-keratins, and 9 keratinocyte β-keratins. The feather β-keratin occupied the largest proportion in golden pheasant, while the number of claw β-keratins was the least (Table S17). The significantly higher expressed genes in feathers were enriched in β-keratins (59 out of

827, Adjust *P*-value=1.23E-61, Table S18). The differentially expressed genes in various colour feathers (white vs iridescent green, white vs red, white vs yellow, iridescent green vs yellow, and iridescent green vs red) were also enriched in β-keratins (Table S19). Compared with white feathers, common DEGs in the three other coloured feathers included 12 β-keratins which were comprised by one claw β-keratin, three feather β-keratins, three scale β-keratins and five keratinocyte β-keratins. In addition, common DEGs between carotenoid contained and non-carotenoid contained feathers included three feather β-keratins. These findings suggest that some of the β-keratins may be related to feather colours. The proportions of the four β-keratin subfamilies to the total number of β-keratins were considered to be associated with avian lifestyles in a previous report [14]. In our investigation, to further detect the relationship between β-keratin and feather colour at the genomic level, we compared the copy number variations of β-keratins in golden pheasant and 51 other avian species. However, no obvious rules have been found between feather colour and copy numbers or subfamily proportions compared to other avian species. Nevertheless, the copy numbers of the β-keratin gene were positively correlated with the quality of the assemblies. The coefficient of determination (R2) between β-keratin copy numbers of β-keratin and contig N50 of each genome assembly was 0.77 (*P*-value=1.99E-16, Pearson's test, Figure S13, S14). We constructed a phylogenetic tree for the β-keratins of six Galliformes and found many clades only contained β-keratins from one species and with small divergence (Figure S15). This phylogeny implied there were independent duplication events after the speciation, resulting the young paralogs with high similarity which may increase the difficulty of the assembly. In the golden pheasant assembly, we found two such instances that a feather keratin protein had three alignments in the golden pheasant assembly with sequencing depth

of 886, and another feather keratin protein had one alignment with a sequencing depth of 957, which were 9-10 times of the mean sequencing depth (92.5) of the whole assembly (Table S20). This indicated that there might be nine and ten copies of these two β-keratins, but that only three and one copies were assembled, respectively, because of the high similarity among different copies. Therefore, it is likely that the copy number of β-keratins was underestimated in most sequenced birds because of the incomplete genome assembly, particular for the recently duplicated copies. As a whole, the DEGs indicate that the β-keratins should be related to feather development, but the further genomics comparison is limited because of the underestimation of the actual copy number. As the assembly level increases following the upgrade of sequencing technologies in the future, particularly the long-read sequencing technologies, the keratins warrant further comprehensive comparative analysis.

Conclusions

In the present study, we provided a genome assembly for the golden pheasant and sequenced a genome of Lady Amherst's pheasant with low coverage. Combined with transcriptome analyses, as well as 51 other birds with available genomes, we studied the plumage colouration in *Chrysolophus*. For melanin pigmentation, through identifying the lineage specific variations in *Chrysolophus*, four melanogenesis genes might be associated with the evolution of eumelanin/pheomelanin regulations in the two pheasants. Additionally, the RNA-seq data showed that the alternative splicing of *ASIP* and *MITF* were consistent with pigment composition in red and yellow feathers of *Chrysolophus*, particularly the *MITF*-1M transcript. For carotenoid pigmentation, we first identified genes that recently varied in golden pheasant but were conserved in the other 40 non-feather-carotenoid birds, and the results indicated that the evolution

of lipid related genes might be highly related to the carotenoids consumption in golden pheasant. Second, by a whole orthologous gene wide association study between the sequenced feather-carotenoid and non-feather-carotenoid birds, we identified 48 candidate genes that contain some lipid-related genes directly or indirectly, which may be associated with the carotenoid deposition in a broad range of avian plumage. In addition, the DEGs between the two pheasants were also enriched in some lipid-related pathways. It could be proposed that extraordinarily complex plumage patterns are not only encoded by the genome but also produced by the mechanisms underlying multi-layered plumage colouring (Figure S16). As a whole, the present genome comparative results provide some insight into the evolution of colour pigmentation, and the transcriptome results show some potential newly regulatory mechanism. However, although the colour is easily observed, the visual estimation may not be accurate because of the complex colouration in feathers. The phenotypes quantified by chemical or physical methods should be more accurate and better for further analysis. However, the quantification for a wide range of birds is not sufficient thus far, particularly for the eumelanin and pheomelanin, which limits the genomic comparison of plumage colouration in a broad range of avian species. Good models can provide much information, and because colouration is an external feature with close relationships, the golden pheasant and its sister pheasant should be adequate models to investigate the evolution and regulation of plumage colouration.

Methods

Genome sequencing and *de novo* assembly

The genomic DNA from blood samples of a male golden pheasant was sequenced on Illumina Hiseq 2000 platform. A series of paired-end sequencing libraries with insert sizes of 170 bp, 500 bp, 800 bp, 2 kb, 5 kb, 10 kb and 20 kb was constructed,

sequenced and assembled using SOAPdenovo [62]. Contigs were constructed by adopting the de Bruijn graph-based algorithm from the clean data short-insert reads (~98.4-fold). Scaffolds were constructed from short reads and long mate-paired information (~138.06-fold).

Taking advantage of the close evolutionary relationship between golden pheasant and turkey, the turkey genome was used as a reference and linked the assembled genome of golden pheasant to construct pseudochromosomes. The genome of golden pheasant was aligned to the genome of turkey using LASTZ (http://www.bx.psu.edu/miller_lab/dist/README.lastz-1.02.00/README.lastz-1.02.00a.html). More details about the method are described in the study of Chinese rhesus macaques genome [63].

Genome annotation

Homology-based and RNA-seq combined data were used to annotate coding genes golden pheasant. For the homology-based prediction, protein sequences of *Gallus gallus*, *Meleagris gallopavo* and *Taeniopygia guttata* were downloaded from Ensembl (release 74) and mapped onto the golden pheasant genome using TblastN [64]. Secondly, high-scoring segment pairs (HSPs) segments were concatenated between the same pair of proteins by Solar. Thirdly, homologous genome sequences were aligned against the matching proteins using Genewise [65] to define accurate gene models. Finally, redundancy was filtered based on the score of the Genewise.

The RNA-seq data are good supplement for gene annotation because most of the homology alignments have no intact ORFs. Almost 100G RNA-seq data from 25 samples were used and assembled them into transcripts as follows. Firstly the reads were mapped to the golden pheasant genome using Tophat (version 2.0.8) [66]. Secondly, Cufflinks [67] was used to assemble transcripts. Thirdly, the longest ORF

from six kinds of phase was selected. Finally, the Genewise's results were extended using the transcripts ORFs as the strategy of Ensembl gene annotation system [68].

Gene functions were assigned according to the best match of the alignment to the public databases, including Swiss-Prot, KEGG and NCBI NR protein databases. Gene Ontology was annotated by Blast2GO based on the alignment with NCBI NR database. The motifs and domains in protein sequences were annotated using InterProScan [36] by searching publicly available databases, including Pfam, PRINTS, PANTHER, PROSITE, ProDom, and SMART.

Tandem repeat searching was carried out using Tandem Repeats Finder [69]. Transposable elements (TEs) in the genome were predicted by a combination of homology-based and *de novo* approaches. For the homology-based prediction, RepeatProteinMask and RepeatMasker [70] against Repbase (http://www.girinst.org/repbase/) [71] were used with default parameters. For the *de novo* approach, RepeatModeler and LTR-FINDER [72] were used to build the *de novo* repeat library, and then RepeatMasker was used to find TEs in the genome using the *de novo* repeat library. For the comparative analysis, the TEs of chicken, turkey and zebra finch were annotated using the same pipeline to avoid the influence of different release of Repbase database or different prediction pipeline.

Transcriptome sequencing

A total of 22 libraries of different organizations or different colour feathers from golden pheasants and Lady Amherst's pheasants (detailed descriptions see Additional file 1) were constructed by using the Illumina TruSeq RNA sample preparation kit according to manufacturer's instructions. The libraries (insertion size ~200 bp) were sequenced 90 bp at each end by using Illumina Hiseq 2000 platform. We achieved 48~83 million reads per library (Table S21). RNA reads were mapped by Tophat

(version 2.0.8) with parameter "-p 6 --b2-very-sensitive --solexa1.3-quals --segment-length 30 --segment-mismatches 2 --read-edit-dist 4 --read-mismatches 4 -r 20 --mate-std-dev 20 --library-type fr-unstranded". Then we quantitated the gene expression level by using unique mapped reads and normalized by using per kilobase of transcript per million mapped reads (RPKM) [73]. For alternative splicing analysis, we quantitated and normalized the junctions by using per million mapped reads (RPM). For detecting DEGs between different individual samples, we used a method described by Chen et al [74]. In the present study, we defined DEGs by using two criteria: a) the RPKM must have at least a two-fold difference; and b) the false discovery rate (FDR) must be less than 0.001. For detecting DEGs between different groups, which can contains multiple samples, we used Noiseq [75] with a cutoff Probability ≥ 0.8. The differentially expressed junctions are identified by DEGseq [76] with MA-plot-based method with a random sampling model.

Phylogenetic analysis and gene family analyses

Treefam pipeline [77] was used to determine orthologous groups among eight birds (golden pheasant, chicken, turkey, Japanese quail, northern bobwhite, scaled quail, zebra finch, and duck). The detailed steps were performed: 1) protein sequences were mapped by BLASTP and to identify potential homologous genes; 2) the raw BlastP results were refined by using Solar, in which the HSPs were conjoined; 3) similarity between protein sequences were evaluated by using bit-score, followed by clustering protein sequences into gene families by using hcluster_sg, a hierarchical clustering algorithm in the TreeFam pipeline (version 0.50) with the parameters of "-w 5 -s 0.33 -m 100000". The identified 6,538 one-to-one orthologous genes among eight species were used to construct the phylogenetic tree. Alignment was performed by using MUSCLE for the protein sequences and then guided to align the corresponding

coding sequences (CDS). A total of 996,755 fourfold degenerate (4D) synonymous sites were obtained and used in the phylogenomic construction. The phylogenome was constructed by using RAxML (version 8.1.19) [78] with the “GTRGAMMA” model. The Bayesian relaxed-molecular clock (BRMC) method, implemented in the MCMCTree program [79] , was used to estimate the divergence time between golden pheasant and other species. Three calibration time points based on Jarvis’s analysis [16], chicken-turkey (28~29 Mya), chicken-duck (65~67 Mya) and chicken-zebra finch (88~90 Mya), were used as constrains in the MCMCTree estimation.

Positively selected genes and gene family evolution

For the 6,538 one-to-one orthologous paired genes (from the TreeFam pipeline as above described) in the eight avian species, the selected positive genes in golden pheasant were investigated. The protein sequences of orthologues were aligned by using the Muscle [80] software with default parameters. Then, the protein alignment was employed as a guide for aligning CDS. All positions with gaps in the alignments were also removed. Positive selection analysis was conducted by using the refined branch-site model [35], which is implemented in the Codeml program of the PAML package (version 4) [79]. *P*-values were computed by using the Chi-square statistics adjusted by the false discovery rate (FDR) method to enable multiple testing and the cut-off was used as 0.01. Further, the selected positive sites were retained by the homology prediction and RNA transcripts to avoid false positive results from the assembly error or different splicing transcripts.

For the multi-copy families, using the gene family results and timed-tree generated in above as inputs, we studied the expansion and contraction of gene families using the CAFE (Computational Analysis of gene Family Evolution, version 2.1) [81] which inferred the dynamics of gene family under a stochastic birth and death model.

The filtering cutoff used the Viterbi *P*-value ≤ 0.01.

SNP and InDel detection in Lady Amherst's pheasant

A total of 46.65 Gb paired-end data (read lengths 100 bp) of the Lady Amherst's pheasant were sequenced from a library with an insert size of 500 bp, and 44.88 Gb high-quality data were generated. All short reads were aligned twice to the golden pheasant genome using SOAP2 (version 2.22) [82]. The first alignment was conducted with an insert size limit of "20 ~ 1000 bp". To reduce the false pair-end alignment, the second alignment was with insert size limit "Median – 3*left-SD (standard deviation) ~ Median + 3*right-SD". Based on the alignment, the single nucleotide polymorphism (SNP) calling was performed by using SOAPsnp [83] , which uses a Bayesian model by carefully considering the character of the Solexa sequencing data and experimental factors. Potential SNPs that met the following criteria were filtered: 1) quality score <20 (on the Phred scale); and 2) the total map depth of this location <5 or >120. Based on the pair-end alignment, the 1-5 bp insertion/deletion variations were identified. To minimize the alignment error, the following set of criteria were applied to the alignment: 1) only one gap, maximum 5 bp, was allowed in a single read; 2) if one read in a pair had a gap in the alignment, the other end had to be gap-free, and the orientation and distance had to meet the parameters of the library; 3) no gap was allowed within 5 bp of the ends of a read; 4) no mismatch was allowed within the gap-containing read; and 5) the total map depth of this location≥5 and ≤120.

By using the assembly of golden pheasant as reference, we obtain the putative gene sequences of Lady Amherst's pheasant by changing the assembly of golden pheasant at the homozygous SNP and InDel detections. The sites with map depth <5 or >120 are replaced by "N".

Lineage specific varied genes in *Chrysolophus*

In the studies of 48 birds as background [15, 16], they used chicken genome as a reference and identified syntenic orthologous genes between chicken and the other 47 birds respectively. At last, they merged the orthologous relationship using the chicken as bridge and got 8295 1:1 syntenic orthologous genes among 48 birds (http://gigadb.org/dataset/view/id/101000#). In the present study, the orthologous genes pairs between the golden pheasant and chicken were identified through the reciprocal best hit (RBH) and gene synteny relationship. The orthologous genes between golden pheasant and chicken were merged to the orthologous genes of 48 birds, forming orthologous set 1 (OS1) of 52 birds. Furthermore, the genes of 49 birds were clustered by using the TreeFam pipeline and identified 595 single copy orthologous families beyond the OS1. Finally, 8,890 orthologous genes of the 49 birds were obtained by merging the OS1 and the TreeFam single-copy families. We also identified syntenic orthologous genes between Japanese quail and chicken, northern bobwhite and chicken, scaled quail and chicken, respectively.

After identifying the orthologous gene pairs, we used 5 other Galliformes (chicken, turkey, Japanese quail, northern bobwhite and scaled quail) and 11 other birds (duck, zebra finch, carmine bee-eater, bald eagle, little egret, emperor penguin, hoatzin, Anna's hummingbird, common cuckoo, pigeon, and common ostrich) from 11 different clades according to the phylogenetic analysis of 48 birds as background. The orthologous protein sequences of golden pheasant, Lady Amherst's pheasant (putative gene sequences as above described), and the 16 birds were aligned using Muscle. The alignments were compared site by site. We selected the gene containing the site which was same in the 16 birds but specific in *Chrysolophus*. To avoid false-positive results from different splicing transcripts, only the results supported by both the homology

prediction and RNA transcripts were retained.

Carotenoids accumulation related genes

Previous investigations have displayed the avian species that expressed carotenoid in their feathers or not [17]. These referenced species overlapped with the genomes published for 48 birds [15, 16] , resulting in four carotenoid-containing and 39 non-carotenoid-containing species with constructed assemblies. Based on these studies, comparative analyses were performed to explore the carotenoid accumulation related candidate genes in golden pheasant by using the following two strategies. 1) Given the close relationship and the difference of carotenoid utilization between the two *Chrysolophus* species, lineage-specific varied genes in golden pheasant as well as conserved in Lady Amherst's pheasant and the other 39 non-carotenoid birds were selected. A multi-sequences alignment was conducted by using MUSCLE to select the genes containing pheasant-specific sites, which is common in Lady Amherst's and the other 39 birds. Finally, the total of 258 recent varied genes were annotated to KEGG pathways and scored by the following methods: i) if pathway A has total number of N(a) genes in golden pheasant and there are number n(a) genes in the 258 recent varied genes, then the score of pathway A, S(A) += n(a)/N(a); and ii) if pathway B has number of O(ab) genes shared with pathway A and has total number of N(b) genes in golden pheasant, then there are number of e(b) genes in pathway B, except the members shared with pathway A, and the S(A) += e(b)/N(b)*O(ab)/N(a).

Otherwise, we referred to the population re-sequencing analysis strategy, such as that in Hilma Holm's research [84], and divided 45 published birds and golden pheasant into carotenoid and non-carotenoid groups. The two uncertain birds with bright yellow or orange or red feathers (golden-collared manakin and bar-tailed trogon) were classified into carotenoid experiential. The genotypes of the carotenoid birds

were examined for randomness among all species by using a hypergeometric site by site test with a *P*-value < 0.001.

Keratin family analysis

To avoid bias from different prediction methods applied in different bird genomes, we download protein sequences of keratin genes of chicken from NCBI, and then mapped against golden pheasant and other 52 avian genomes by using the same pipeline. Homology-based gene prediction was obtained by using the gene prediction pipeline mentioned above, except the threshold alignment rate was greater than 50%. Domain annotation was performed by using InterProScan, and only the results with domain of IPR003461 (avian keratin), IPR002957 (Type I keratin), or IPR003054 (Type II keratin) were retained. The correlation between the copy number of β-keratin and the assembly quality (contig N50) refer to the studies of the 48 birds (part of "Correlation between average substitution rates and number of species within different avian orders" and "Colour Discriminability") [15]. The subfamilies of β-keratins were classified based on the best hit to the β-keratins of zebra finch which had been classified by Greenwold. We also performed another version of this study by using the keratin gene numbers from evolutionary research of the keratins in the 48 birds [14].

Pigments identification

Both melanin and carotenoid pigments in feathers were examined in two ways, methods of spectrum and chromatogram. Raman spectroscopy was carried out through a Labram HR1800 spectrometer (HORIBA JobinYvon, France), referring the strategy of Galvan [85] and Thomas [49] for melanin and carotenoid detection, respectively. High-performance liquid chromatography was carried out through an SIL-20A HPLC system equipped with an SPD-20A UV/Vis detector (Shimadzu, Japan), referring the strategy of McGraw [86] and Wakamatsu [34] for melanin and

carotenoid detection, respectively. More details of pigment identification are described in Supplementary Notes 1.

Additional files

Additional file 1: This doc file contains the supplementary figures: S1–S20.

Additional file 2: This xls file contains the supplementary tables: S1-S24.

Additional file 3: This doc file contains supplementary notes of pigments identification, animal sampling and transcriptome analysis.

ACKNOWLEDGEMENTS

We owe many thanks to Dr. Kazumasa Wakamatsu from Fujita Health University for providing the TTCA and PTCA standards. We are indebted to Cai Li and Hailin Pan from China National GeneBank for giving advices in genome analysis. We are also grateful to Tianyuan Wang from Yuanfeng wild animal farm (Jilin province, China) for taking care of the experimental birds. Many thanks to the people whose names are not included in the author list, but did some contribution to this project.

FUNDING

This research was partly funded by the State Key Development Program for Basic Research of China, 973 Program (2012CB22306), the Open Project of Key Development Program for Basic Research of Inner Mongolia Autonomous Region, National Natural Science Foundation of China (30960244), State Key Laboratory of Agricultural Genomics (No. 2011DQ782025).

DATA AVAILABILITY

Genome assembly has been deposited in GenBank. The *Chrysolophus pictus* genome assembly has been deposited under the accession number SAMN02980944. The

datasets (assembly and annotation files) supporting the results of this article have been deposited in the GigaScience database [87].

Author's contributions

GPL, JY and CZ conceived the study. JY, GQG, YCZ, JG and XKY prepared the samples. MX, JMM, YLY HMC and CZ performed genome sequencing, assembly and annotation. GPL and CZ supervised genome sequencing, assembly and annotation. MX, GQG, YLY, JMM, XQZ, XMX and JYX performed genome analyses. GQG, MX, CLB and GHS carried out the transcriptome analyses. GQG, ZYW and YH carried out carotenoids and eu-/pheomelanins analysis. RBW, GPZ and CMC discussed the data. All authors contributed to data interpretation. GPL, GQG and MX wrote the paper with significant contributions from YCZ, CLB, JY, CMC and CZ.

Competing interests

The authors declare no competing financial interests.

Ethics approval and consent to participate

This study was approved by the Institutional Animal Care and Use Committee of the Inner Mongolia University.

References

1. Hill G and McGraw K. Bird coloration Vol. 2. Function and Evolution. Massachusetts: Harvard University Press, Cambridge; 2006.
2. Hill G and McGraw K. Bird coloration Vol. 1. Mechanisms and measurements. Massachusetts: Harvard University Press, Cambridge; 2006.
3. Prum RO, LaFountain AM, Berro J, Stoddard MC and Frank HA. Molecular diversity, metabolic transformation, and evolution of carotenoid feather pigments in cotingas (Aves: Cotingidae). J Comp Physiol B. 2012;182 8:1095-116. doi:10.1007/s00360-012-0677-4.
4. Negro JJ, Bortolotti GR, Mateo R and Garcia IM. Porphyrins and pheomelanins contribute to the reddish juvenal plumage of black-shouldered kites. Comp Biochem Physiol B Biochem Mol Biol. 2009;153 3:296-9. doi:10.1016/j.cbpb.2009.03.013.
5. McGraw KJ and Nogare MC. Distribution of unique red feather pigments in parrots. Biol Lett. 2005;1 1:38-43. doi:10.1098/rsbl.2004.0269.

6. Toral GM, Figuerola J and Negro JJ. Multiple ways to become red: pigment identification in red feathers using spectrometry. Comp Biochem Physiol B Biochem Mol Biol. 2008;150 2:147-52. doi:10.1016/j.cbpb.2008.02.006.
7. Slominski A, Tobin DJ, Shibahara S and Wortsman J. Melanin pigmentation in mammalian skin and its hormonal regulation. Physiol Rev. 2004;84 4:1155-228. doi:10.1152/physrev.00044.2003.
8. Schiaffino MV. Signaling pathways in melanosome biogenesis and pathology. Int J Biochem Cell Biol. 2010;42 7:1094-104. doi:10.1016/j.biocel.2010.03.023.
9. Roulin A and Ducrest AL. Genetics of colouration in birds. Semin Cell Dev Biol. 2013;24 6-7:594-608. doi:10.1016/j.semcdb.2013.05.005.
10. Simon JD, Peles D, Wakamatsu K and Ito S. Current challenges in understanding melanogenesis: bridging chemistry, biological control, morphology, and function. Pigment Cell Melanoma Res. 2009;22 5:563-79. doi:10.1111/j.1755-148X.2009.00610.x.
11. Walsh N, Dale J, McGraw KJ, Pointer MA and Mundy NI. Candidate genes for carotenoid coloration in vertebrates and their expression profiles in the carotenoid-containing plumage and bill of a wild bird. Proc Biol Sci. 2012;279 1726:58-66. doi:10.1098/rspb.2011.0765.
12. Maia R, Macedo RH and Shawkey MD. Nanostructural self-assembly of iridescent feather barbules through depletion attraction of melanosomes during keratinization. J R Soc Interface. 2012;9 69:734-43. doi:10.1098/rsif.2011.0456.
13. Ng CS, Wu P, Foley J, Foley A, McDonald ML, Juan WT, et al. The chicken frizzle feather is due to an alpha-keratin (KRT75) mutation that causes a defective rachis. PLoS Genet. 2012;8 7:e1002748. doi:10.1371/journal.pgen.1002748.
14. Greenwold MJ, Bao W, Jarvis ED, Hu H, Li C, Gilbert MT, et al. Dynamic evolution of the alpha (alpha) and beta (beta) keratins has accompanied integument diversification and the adaptation of birds into novel lifestyles. BMC Evol Biol. 2014;14:249. doi:10.1186/s12862-014-0249-1.
15. Zhang G, Li C, Li Q, Li B, Larkin DM, Lee C, et al. Comparative genomics reveals insights into avian genome evolution and adaptation. Science. 2014;346 6215:1311-20. doi:10.1126/science.1251385.
16. Jarvis ED, Mirarab S, Aberer AJ, Li B, Houde P, Li C, et al. Whole-genome analyses resolve early branches in the tree of life of modern birds. Science. 2014;346 6215:1320-31. doi:10.1126/science.1253451.
17. Thomas DB, McGraw KJ, Butler MW, Carrano MT, Madden O and James HF. Ancient origins and multiple appearances of carotenoid-pigmented feathers in birds. Proc Biol Sci. 2014;281 1788:20140806. doi:10.1098/rspb.2014.0806.
18. Kawahara-Miki R, Sano S, Nunome M, Shimmura T, Kuwayama T, Takahashi S, et al. Next-generation sequencing reveals genomic features in the Japanese quail. Genomics. 2013;101 6:345-53. doi:10.1016/j.ygeno.2013.03.006.
19. Oldeschulte DL, Halley YA, Wilson ML, Bhattarai EK, Brashear W, Hill J, et al. Annotated Draft Genome Assemblies for the Northern Bobwhite (Colinus virginianus) and the Scaled Quail (Callipepla squamata) Reveal Disparate Estimates of Modern Genome Diversity and Historic Effective Population Size. G3 (Bethesda). 2017;7 9:3047-58. doi:10.1534/g3.117.043083.
20. Marino-Ramirez L, Lewis KC, Landsman D and Jordan IK. Transposable elements donate lineage-specific regulatory sequences to host genomes. Cytogenet Genome Res. 2005;110

1-4:333-41. doi:10.1159/000084965.

21. Naito K, Zhang F, Tsukiyama T, Saito H, Hancock CN, Richardson AO, et al. Unexpected consequences of a sudden and massive transposon amplification on rice gene expression. Nature. 2009;461 7267:1130-4. doi:10.1038/nature08479.

22. Bolger A, Scossa F, Bolger ME, Lanz C, Maumus F, Tohge T, et al. The genome of the stress-tolerant wild tomato species Solanum pennellii. Nat Genet. 2014;46 9:1034-8. doi:10.1038/ng.3046.

23. Wallis JW, Aerts J, Groenen MA, Crooijmans RP, Layman D, Graves TA, et al. A physical map of the chicken genome. Nature. 2004;432 7018:761-4. doi:10.1038/nature03030.

24. Dalloul RA, Long JA, Zimin AV, Aslam L, Beal K, Blomberg Le A, et al. Multi-platform next-generation sequencing of the domestic turkey (Meleagris gallopavo): genome assembly and analysis. PLoS Biol. 2010;8 9 doi:10.1371/journal.pbio.1000475.

25. Huang Y, Li Y, Burt DW, Chen H, Zhang Y, Qian W, et al. The duck genome and transcriptome provide insight into an avian influenza virus reservoir species. Nat Genet. 2013;45 7:776-83. doi:10.1038/ng.2657.

26. Warren WC, Clayton DF, Ellegren H, Arnold AP, Hillier LW, Kunstner A, et al. The genome of a songbird. Nature. 2010;464 7289:757-62. doi:10.1038/nature08819.

27. Wang N, Kimball RT, Braun EL, Liang B and Zhang Z. Assessing phylogenetic relationships among galliformes: a multigene phylogeny with expanded taxon sampling in Phasianidae. PLoS One. 2013;8 5:e64312. doi:10.1371/journal.pone.0064312.

28. Kondrashov FA. Gene duplication as a mechanism of genomic adaptation to a changing environment. Proc Biol Sci. 2012;279 1749:5048-57. doi:10.1098/rspb.2012.1108.

29. Mundy NI, Stapley J, Bennison C, Tucker R, Twyman H, Kim KW, et al. Red Carotenoid Coloration in the Zebra Finch Is Controlled by a Cytochrome P450 Gene Cluster. Curr Biol. 2016;26 11:1435-40. doi:10.1016/j.cub.2016.04.047.

30. Emerling CA. Independent pseudogenization of CYP2J19 in penguins, owls and kiwis implicates gene in red carotenoid synthesis. Mol Phylogenet Evol. 2018;118:47-53. doi:10.1016/j.ympev.2017.09.016.

31. Lopes RJ, Johnson JD, Toomey MB, Ferreira MS, Araujo PM, Melo-Ferreira J, et al. Genetic Basis for Red Coloration in Birds. Curr Biol. 2016;26 11:1427-34. doi:10.1016/j.cub.2016.03.076.

32. von Schantz T, Bensch S, Grahn M, Hasselquist D and Wittzell H. Good genes, oxidative stress and condition-dependent sexual signals. Proc Biol Sci. 1999;266 1414:1-12. doi:10.1098/rspb.1999.0597.

33. Ingelman-Sundberg M. Genetic polymorphisms of cytochrome P450 2D6 (CYP2D6): clinical consequences, evolutionary aspects and functional diversity. Pharmacogenomics J. 2005;5 1:6-13. doi:10.1038/sj.tpj.6500285.

34. Ito S, Nakanishi Y, Valenzuela RK, Brilliant MH, Kolbe L and Wakamatsu K. Usefulness of alkaline hydrogen peroxide oxidation to analyze eumelanin and pheomelanin in various tissue samples: application to chemical analysis of human hair melanins. Pigm Cell Melanoma R. 2011;24 4:605-13. doi:10.1111/j.1755-148X.2011.00864.x.

35. Zhang J, Nielsen R and Yang Z. Evaluation of an improved branch-site likelihood method for detecting positive selection at the molecular level. Mol Biol Evol. 2005;22 12:2472-9. doi:10.1093/molbev/msi237.

36. Jones P, Binns D, Chang HY, Fraser M, Li W, McAnulla C, et al. InterProScan 5: genome-scale protein function classification. Bioinformatics. 2014;30 9:1236-40. doi:10.1093/bioinformatics/btu031.

37. Hida T, Wakamatsu K, Sviderskaya EV, Donkin AJ, Montoliu L, Lynn Lamoreux M, et al. Agouti protein, mahogunin, and attractin in pheomelanogenesis and melanoblast-like alteration of melanocytes: a cAMP-independent pathway. Pigment Cell Melanoma Res. 2009;22 5:623-34. doi:10.1111/j.1755-148X.2009.00582.x.

38. Levy C, Khaled M and Fisher DE. MITF: master regulator of melanocyte development and melanoma oncogene. Trends Mol Med. 2006;12 9:406-14. doi:10.1016/j.molmed.2006.07.008.

39. Wu M, Hemesath TJ, Takemoto CM, Horstmann MA, Wells AG, Price ER, et al. c-Kit triggers dual phosphorylations, which couple activation and degradation of the essential melanocyte factor Mi. Genes Dev. 2000;14 3:301-12.

40. Sato-Jin K, Nishimura EK, Akasaka E, Huber W, Nakano H, Miller A, et al. Epistatic connections between microphthalmia-associated transcription factor and endothelin signaling in Waardenburg syndrome and other pigmentary disorders. FASEB J. 2008;22 4:1155-68. doi:10.1096/fj.07-9080com.

41. Moroy T and Heyd F. The impact of alternative splicing in vivo: mouse models show the way. RNA. 2007;13 8:1155-71. doi:10.1261/rna.554607.

42. Vrieling H, Duhl DM, Millar SE, Miller KA and Barsh GS. Differences in dorsal and ventral pigmentation result from regional expression of the mouse agouti gene. Proc Natl Acad Sci U S A. 1994;91 12:5667-71.

43. Fontanesi L, Forestier L, Allain D, Scotti E, Beretti F, Deretz-Picoulet S, et al. Characterization of the rabbit agouti signaling protein (ASIP) gene: transcripts and phylogenetic analyses and identification of the causative mutation of the nonagouti black coat colour. Genomics. 2010;95 3:166-75. doi:10.1016/j.ygeno.2009.11.003.

44. Yoshihara C, Fukao A, Ando K, Tashiro Y, Taniuchi S, Takahashi S, et al. Elaborate color patterns of individual chicken feathers may be formed by the agouti signaling protein. Gen Comp Endocrinol. 2012;175 3:495-9. doi:10.1016/j.ygcen.2011.12.009.

45. Oribe E, Fukao A, Yoshihara C, Mendori M, Rosal KG, Takahashi S, et al. Conserved distal promoter of the agouti signaling protein (ASIP) gene controls sexual dichromatism in chickens. Gen Comp Endocrinol. 2012;177 2:231-7. doi:10.1016/j.ygcen.2012.04.016.

46. Poelstra JW, Vijay N, Bossu CM, Lantz H, Ryll B, Muller I, et al. The genomic landscape underlying phenotypic integrity in the face of gene flow in crows. Science. 2014;344 6190:1410-4. doi:10.1126/science.1253226.

47. Maruotti J, Thein T, Zack DJ and Esumi N. MITF-M, a 'melanocyte-specific' isoform, is expressed in the adult retinal pigment epithelium. Pigment Cell Melanoma Res. 2012;25 5:641-4. doi:10.1111/j.1755-148X.2012.01033.x.

48. Toews DP, Hofmeister NR and Taylor SA. The Evolution and Genetics of Carotenoid Processing in Animals. Trends Genet. 2017;33 3:171-82. doi:10.1016/j.tig.2017.01.002.

49. Thomas DB, McGraw KJ, James HF and Madden O. Non-destructive descriptions of carotenoids in feathers using Raman spectroscopy. Anal Methods-Uk. 2014;6 5:1301-8. doi:10.1039/c3ay41870g.

50. Marie H, Pratt SJ, Betson M, Epple H, Kittler JT, Meek L, et al. The LIM protein Ajuba is

recruited to cadherin-dependent cell junctions through an association with alpha-catenin. J Biol Chem. 2003;278 2:1220-8. doi:10.1074/jbc.M205391200.

51. Li B and Trueb B. Analysis of the alpha-actinin/zyxin interaction. J Biol Chem. 2001;276 36:33328-35. doi:10.1074/jbc.M100789200.

52. Hihi AK, Michalik L and Wahli W. PPARs: transcriptional effectors of fatty acids and their derivatives. Cell Mol Life Sci. 2002;59 5:790-8.

53. Savage DB. PPAR gamma as a metabolic regulator: insights from genomics and pharmacology. Expert Rev Mol Med. 2005;7 1:1-16. doi:10.1017/S1462399405008793.

54. Poelstra JW, Ellegren H and Wolf JBW. An extensive candidate gene approach to speciation: diversity, divergence and linkage disequilibrium in candidate pigmentation genes across the European crow hybrid zone. Heredity. 2013;111 6:467-73. doi:10.1038/hdy.2013.68.

55. Mein JR, Dolnikowski GG, Ernst H, Russell RM and Wang XD. Enzymatic formation of apo-carotenoids from the xanthophyll carotenoids lutein, zeaxanthin and beta-cryptoxanthin by ferret carotene-9',10'-monooxygenase. Arch Biochem Biophys. 2011;506 1:109-21. doi:10.1016/j.abb.2010.11.005.

56. Vage DI and Boman IA. A nonsense mutation in the beta-carotene oxygenase 2 (BCO2) gene is tightly associated with accumulation of carotenoids in adipose tissue in sheep (Ovis aries). Bmc Genet. 2010;11 doi:Artn 10
10.1186/1471-2156-11-10.

57. Tian R, Pitchford WS, Morris CA, Cullen NG and Bottema CDK. Genetic variation in the beta, beta-carotene-9 ', 10 '-dioxygenase gene and association with fat colour in bovine adipose tissue and milk. Anim Genet. 2010;41 3:253-9. doi:10.1111/j.1365-2052.2009.01990.x.

58. Li BX, Vachali PP, Gorusupudi A, Shen ZQ, Sharifzadeh H, Besch BM, et al. Inactivity of human beta,beta-carotene-9 ', 10 '-dioxygenase (BCO2) underlies retinal accumulation of the human macular carotenoid pigment. P Natl Acad Sci USA. 2014;111 28:10173-8. doi:10.1073/pnas.1402526111.

59. Eriksson J, Larson G, Gunnarsson U, Bed'hom B, Tixier-Boichard M, Stromstedt L, et al. Identification of the Yellow skin gene reveals a hybrid origin of the domestic chicken. Plos Genetics. 2008;4 2 doi:ARTN e1000010
10.1371/journal.pgen.1000010.

60. Toews DP, Taylor SA, Vallender R, Brelsford A, Butcher BG, Messer PW, et al. Plumage Genes and Little Else Distinguish the Genomes of Hybridizing Warblers. Curr Biol. 2016;26 17:2313-8. doi:10.1016/j.cub.2016.06.034.

61. Greenwold MJ and Sawyer RH. Genomic organization and molecular phylogenies of the beta (beta) keratin multigene family in the chicken (Gallus gallus) and zebra finch (Taeniopygia guttata): implications for feather evolution. BMC Evol Biol. 2010;10:148. doi:10.1186/1471-2148-10-148.

62. Li R, Zhu H, Ruan J, Qian W, Fang X, Shi Z, et al. De novo assembly of human genomes with massively parallel short read sequencing. Genome Res. 2010;20 2:265-72. doi:10.1101/gr.097261.109.

63. Yan G, Zhang G, Fang X, Zhang Y, Li C, Ling F, et al. Genome sequencing and comparison of two nonhuman primate animal models, the cynomolgus and Chinese rhesus macaques. Nat Biotechnol. 2011;29 11:1019-23. doi:10.1038/nbt.1992.

64. Kent WJ. BLAT - The BLAST-like alignment tool. Genome Res. 2002;12 4:656-64. doi:Doi

10.1101/Gr.229202.
65. Birney E, Clamp M and Durbin R. GeneWise and genomewise. Genome Res. 2004;14 5:988-95. doi:Doi 10.1101/Gr.1865504.
66. Trapnell C, Pachter L and Salzberg SL. TopHat: discovering splice junctions with RNA-Seq. Bioinformatics. 2009;25 9:1105-11. doi:DOI 10.1093/bioinformatics/btp120.
67. Trapnell C, Williams BA, Pertea G, Mortazavi A, Kwan G, van Baren MJ, et al. Transcript assembly and quantification by RNA-Seq reveals unannotated transcripts and isoform switching during cell differentiation. Nat Biotechnol. 2010;28 5:511-U174. doi:Doi 10.1038/Nbt.1621.
68. Curwen V, Eyras E, Andrews TD, Clarke L, Mongin E, Searle SM, et al. The Ensembl automatic gene annotation system. Genome Res. 2004;14 5:942-50. doi:10.1101/gr.1858004.
69. Benson G. Tandem repeats finder: a program to analyze DNA sequences. Nucleic Acids Research. 1999;27 2:573-80. doi:DOI 10.1093/nar/27.2.573.
70. Chen N. Using RepeatMasker to identify repetitive elements in genomic sequences. Curr Protoc Bioinformatics. 2004;Chapter 4:Unit 4 10. doi:10.1002/0471250953.bi0410s05.
71. Jurka J, Kapitonov VV, Pavlicek A, Klonowski P, Kohany O and Walichiewicz J. Repbase Update, a database of eukaryotic repetitive elements. Cytogenet Genome Res. 2005;110 1-4:462-7. doi:10.1159/000084979.
72. Xu Z and Wang H. LTR_FINDER: an efficient tool for the prediction of full-length LTR retrotransposons. Nucleic Acids Res. 2007;35 Web Server issue:W265-8. doi:10.1093/nar/gkm286.
73. Mortazavi A, Williams BA, McCue K, Schaeffer L and Wold B. Mapping and quantifying mammalian transcriptomes by RNA-Seq. Nat Methods. 2008;5 7:621-8. doi:10.1038/nmeth.1226.
74. Chen S, Yang P, Jiang F, Wei Y, Ma Z and Kang L. De novo analysis of transcriptome dynamics in the migratory locust during the development of phase traits. PLoS One. 2010;5 12:e15633. doi:10.1371/journal.pone.0015633.
75. Tarazona S, Garcia-Alcalde F, Dopazo J, Ferrer A and Conesa A. Differential expression in RNA-seq: a matter of depth. Genome Res. 2011;21 12:2213-23. doi:10.1101/gr.124321.111.
76. Wang L, Feng Z, Wang X, Wang X and Zhang X. DEGseq: an R package for identifying differentially expressed genes from RNA-seq data. Bioinformatics. 2010;26 1:136-8. doi:10.1093/bioinformatics/btp612.
77. Li H, Coghlan A, Ruan J, Coin LJ, Heriche JK, Osmotherly L, et al. TreeFam: a curated database of phylogenetic trees of animal gene families. Nucleic Acids Res. 2006;34 Database issue:D572-80. doi:10.1093/nar/gkj118.
78. Stamatakis A. RAxML-VI-HPC: maximum likelihood-based phylogenetic analyses with thousands of taxa and mixed models. Bioinformatics. 2006;22 21:2688-90. doi:10.1093/bioinformatics/btl446.
79. Yang Z. PAML 4: phylogenetic analysis by maximum likelihood. Mol Biol Evol. 2007;24 8:1586-91. doi:10.1093/molbev/msm088.
80. Wheeler TJ and Kececioglu JD. Multiple alignment by aligning alignments. Bioinformatics. 2007;23 13:i559-68. doi:10.1093/bioinformatics/btm226.
81. De Bie T, Cristianini N, Demuth JP and Hahn MW. CAFE: a computational tool for the study of gene family evolution. Bioinformatics. 2006;22 10:1269-71.

doi:10.1093/bioinformatics/btl097.
82. Li R, Yu C, Li Y, Lam TW, Yiu SM, Kristiansen K, et al. SOAP2: an improved ultrafast tool for short read alignment. Bioinformatics. 2009;25 15:1966-7. doi:10.1093/bioinformatics/btp336.
83. Li R, Li Y, Fang X, Yang H, Wang J, Kristiansen K, et al. SNP detection for massively parallel whole-genome resequencing. Genome Res. 2009;19 6:1124-32. doi:10.1101/gr.088013.108.
84. Holm H, Gudbjartsson DF, Sulem P, Masson G, Helgadottir HT, Zanon C, et al. A rare variant in MYH6 is associated with high risk of sick sinus syndrome. Nat Genet. 2011;43 4:316-20. doi:10.1038/ng.781.
85. Galvan I, Jorge A, Ito K, Tabuchi K, Solano F and Wakamatsu K. Raman spectroscopy as a non-invasive technique for the quantification of melanins in feathers and hairs. Pigment Cell Melanoma Res. 2013;26 6:917-23. doi:10.1111/pcmr.12140.
86. McGraw KJ, Hill GE, Stradi R and Parker RS. The effect of dietary carotenoid access on sexual dichromatism and plumage pigment composition in the American goldfinch. Comp Biochem Physiol B Biochem Mol Biol. 2002;131 2:261-9.
87. Gao GQ, Xu M, Zuo YC. Supporting data for "Title of your manuscript". GigaScience Database. 2017. http://dx.doi.orgxxxxxxxxxxxx

Figure legends

Figure 1 Profile of golden pheasant (upper right) and Lady Amherst's pheasant (upper left) and their feathers from different body parts (lower part). Both male species (near) are more colourful than females (far). The female feathers are represented by the napes.

Figure 2 Comparative genomic analyses among the golden pheasant and other avian species. (a) Global view of the golden pheasant genome using the pseudochromosomes. **(b)** The Maximum Likelihood phylogenetic relationships of the golden pheasant in Galliformes. The tree was constructed based on 996,755 bp 4-fold degenerate sites, from 6,538 single-copy orthologous genes among six sequenced Galliformes genomes (golden pheasant, chicken, turkey, Japanese quail, northern bobwhite and scaled quail) the sequenced Anseriformes (duck) and the zebra finch (as

outgroup). **(c)** Venn diagram of the shared orthologous gene families among the Galliformes species. **(d)** The maximum likelihood phylogeny tree of CYP2D genes in 17 avian species. The background species are selected based on Galliformes species and Jarvis's phylogeny for the 48 avian genomes, of which 11 birds with high quality of genome build from ten different clades are selected in this analysis.

Figure 3 The variation and alternative splicing of some regulator genes in the eu-/pheomelanin synthesis metabolism. **(a)** The pathway of Eu-/pheomelanin synthesis metabolism. The lineage specific varied genes in *Chrysolophus* are marked by red star. The significant higher expressed genes in feathers with green, red, and yellow colour, are marked by the colourful rectangular respectively, all use the white feathers (A-F-Nape and A-F-Belly) as control. **(b)** The single nucleotide insertion in the ASIP gene of the *Chrysolophus*. A base of adenine inserts after the initiation codon of the ORF at exon 2A. This insertion was verified in another five *Chrysolophus* individuals (lower part). "SN", sample name; "ST", sequencing type, R, RNA sequencing, D, DNA sequencing; "Ta/To", the number of reads support the shown genotype/the number of total mapped reads. **(c)** The RNA alternative splicing of *ASIP* gene. The upper section is the alternative splicing models of *ASIP*. Rectangles represent exons, and curves represent junctions between the exons. The size scale ratio between exons and introns is 1:10. The lower section is expression histogram of the junctions. RPM (Reads per million mapped reads) was used to normalize expression levels. The colour of the column matches the accepter exon colour. The colour of the footstone matches the donor exon colour. **(d)** The RNA alternative splicing of *MITF* gene. Descriptions are same with Fig. 3c. The description of sample name: "P-", golden pheasant; "A-", Lady Amherst's pheasant; "-F-", feather; "-S-",

skin.

Figure 4 The comparative analysis and RNA expression of the carotenoid accumulation in feather. (a) The high performance liquid chromatography (HPLC) analysis of lutein and zeaxanthin in *Chrysolophus* red and yellow feathers. **(b)** The KEGG pathway annotation of the genes which is lineage-specific in golden pheasant but same in other non-feather-carotenoid 40 birds. The scoring standard of each pathway is described in the method. **(c)** The orthologous genes wide association study to the carotenoids accumulation. The coordinates are based on chicken chromosomes. Dashed line indicates the connected sites belonged to the same gene. The green spots are genes that also contain lineage-specific varied sites in golden pheasant. The orange spots are lipid related genes. **(d)** The theoretical process of carotenoids transportation and deposition. **(e)** The KEGG pathway enrichment of the union DEGs between feather follicles of the two pheasants. The "RichFactor" = the number of DEGs in this pathway/the number of gene set in this pathway. More details are described in Additional file 3: Notes 4.2. **(f)** The expression of the *APOA*1 and *BCO*2 gene in the two pheasants.

Table 1 Statistics of assembly and annotation for the golden pheasant genome

Genome characteristics	Data
Assembly features	
Estimate of genome size	1,032,423,981 bp
Total size of assembled scaffolds	1,028,603,357 bp
Scaffold N50	1,547,393 bp
Longest scaffold	18,323,375 bp
Total size of assembled contigs	1,003,285,807 bp
Contig N50	34,356 bp
Longest contig	257,270 bp
GC content (excluding Ns)	40.80%
Annotation features	
Number of gene models	15,552
Mean coding sequence length	1705.27 bp
Mean number of exons per gene	9.94
Mean exon length	171.62 bp
Mean intron length	2397.68 bp
Total size of REs	112,429,773 bp
REs share in genome	10.93%

*RE, repetitive elements.

 Click here to access/download;Figure;revised Figures.doc

Figures

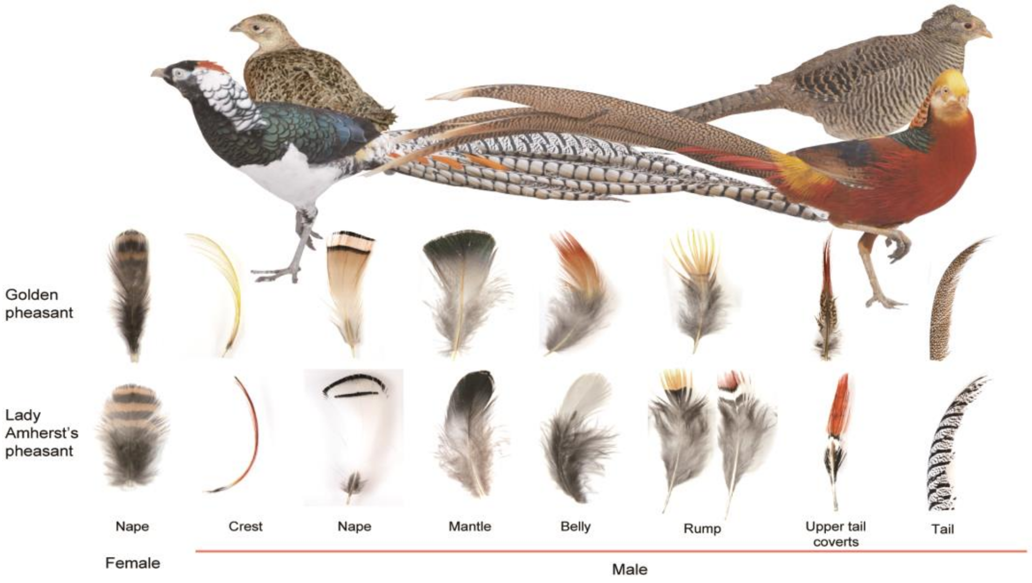

Figure 1 Profile of golden pheasant (upper right) and Lady Amherst's pheasant (upper left) and their feathers from different body parts (lower part). Both male species (near) are more colourful than females (far). The female feathers are represented by the napes.

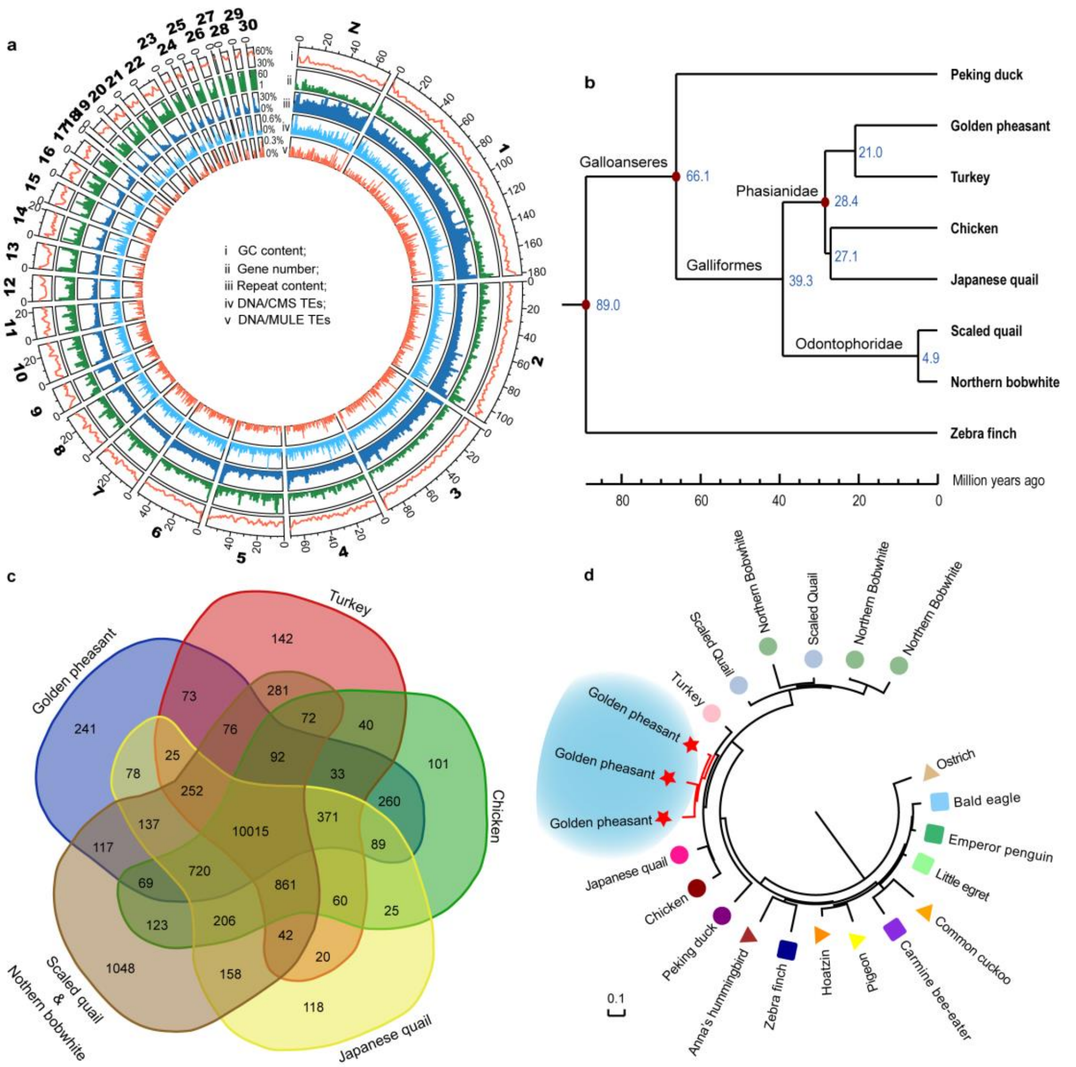

Figure 2 Comparative genomic analyses among the golden pheasant and other avian species. (a) Global view of the golden pheasant genome using the pseudochromosomes. **(b)** The Maximum Likelihood phylogenetic relationships of the golden pheasant in Galliformes. The tree was constructed based on 996,755 bp 4-fold degenerate sites, from 6,538 single-copy orthologous genes among six sequenced Galliformes genomes (golden pheasant, chicken, turkey, Japanese quail, northern bobwhite and scaled quail) the sequenced Anseriformes (duck) and the zebra finch (as outgroup). **(c)** Venn diagram of the shared orthologous gene families among the Galliformes species. **(d)** The maximum likelihood phylogeny tree of CYP2D genes in 17 avian species. The background species are selected based on Galliformes species

and Jarvis's phylogeny for the 48 avian genomes, of which 11 birds with high quality of genome build from ten different clades are selected in this analysis.

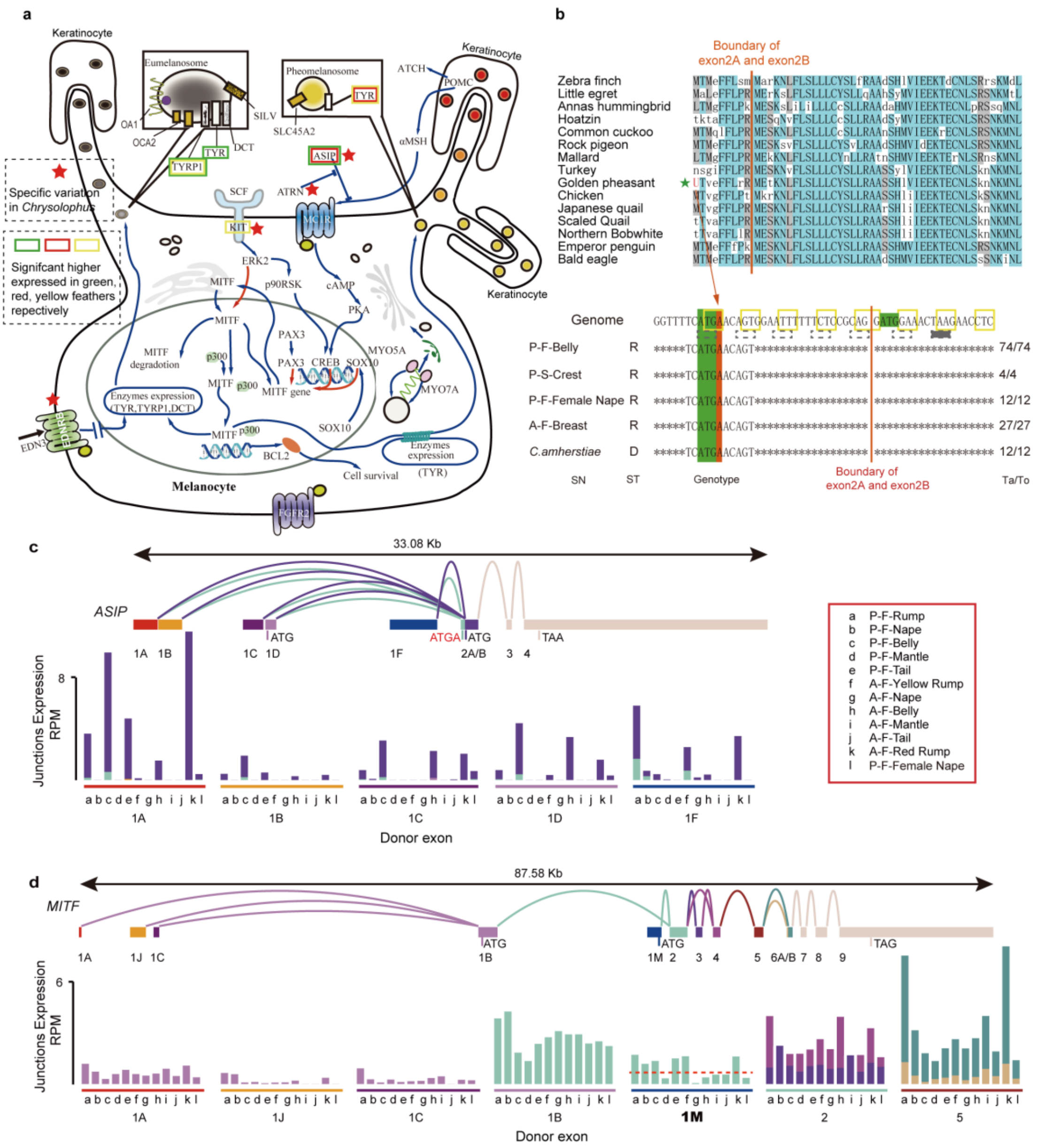

Figure 3 The variation and alternative splicing of some regulator genes in the eu-/pheomelanin synthesis metabolism. (a) The pathway of Eu-/pheomelanin synthesis metabolism. The lineage specific varied genes in *Chrysolophus* are marked by red star. The significant higher expressed genes in feathers with green, red, and yellow colour, are marked by the colourful rectangular respectively, all use the white feathers (A-F-Nape and A-F-Belly) as control. **(b)** The single nucleotide insertion in the ASIP gene of the *Chrysolophus*. A base of adenine inserts after the initiation codon of the ORF at exon 2A. This insertion was verified in another five *Chrysolophus*

individuals (lower part). “SN”, sample name; “ST”, sequencing type, R, RNA sequencing, D, DNA sequencing; “Ta/To”, the number of reads support the shown genotype/the number of total mapped reads. **(c)** The RNA alternative splicing of *ASIP* gene. The upper section is the alternative splicing models of *ASIP*. Rectangles represent exons, and curves represent junctions between the exons. The size scale ratio between exons and introns is 1:10. The lower section is expression histogram of the junctions. RPM (Reads per million mapped reads) was used to normalize expression levels. The colour of the column matches the accepter exon colour. The colour of the footstone matches the donor exon colour. **(d)** The RNA alternative splicing of *MITF* gene. Descriptions are same with Fig. 3c. The description of sample name: “P-”, golden pheasant; “A-”, Lady Amherst’s pheasant; “-F-”, feather; “-S-”, skin.

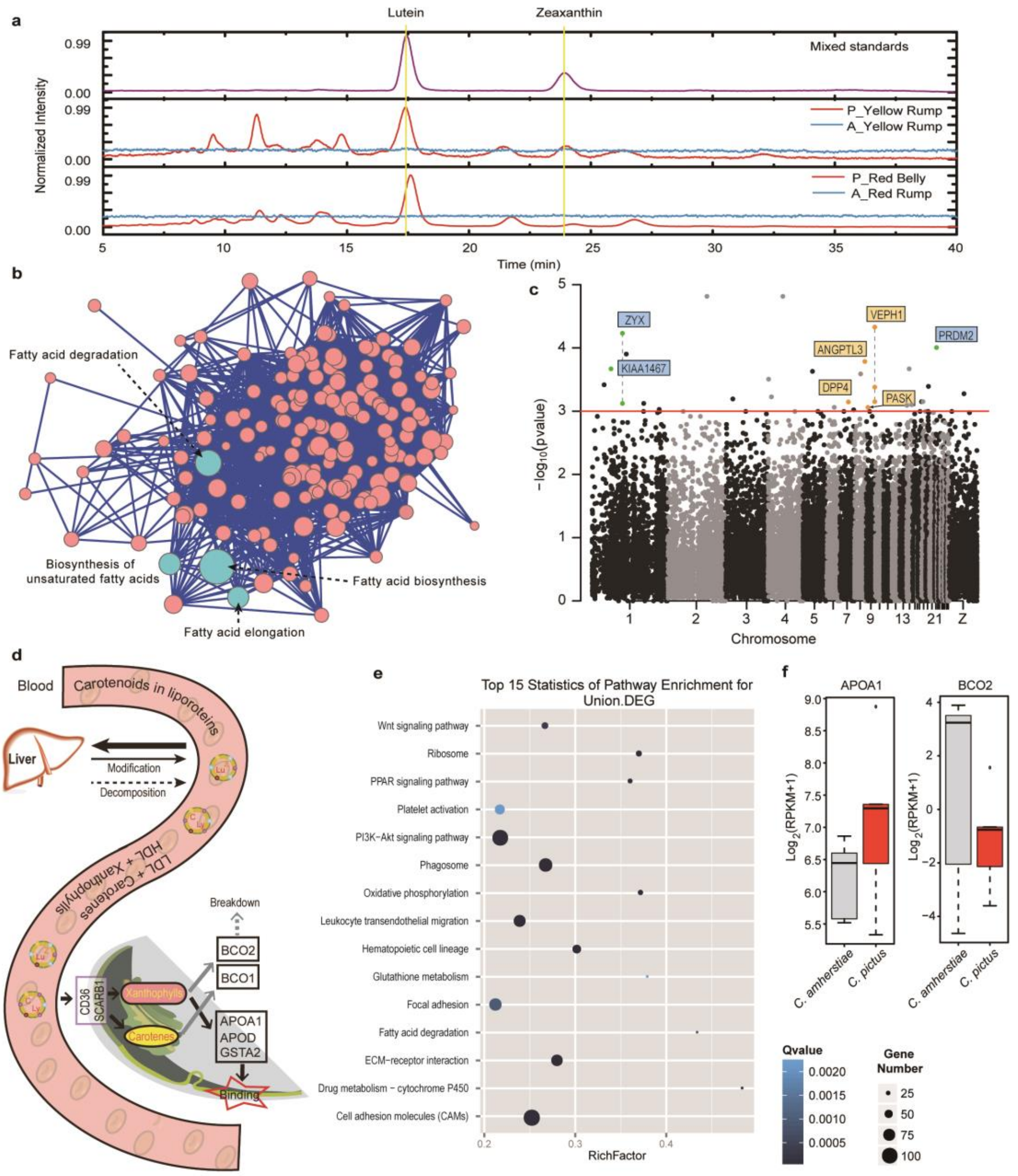

Figure 4 The comparative analysis and RNA expression of the carotenoid accumulation in feather. (a) The high performance liquid chromatography (HPLC) analysis of lutein and zeaxanthin in *Chrysolophus* red and yellow feathers. **(b)** The KEGG pathway annotation of the genes which is lineage-specific in golden pheasant but same in other non-feather-carotenoid 40 birds. The scoring standard of each pathway is described in the method. **(c)** The orthologous genes wide association study to the carotenoids accumulation. The coordinates are based on chicken chromosomes. Dashed line indicates the connected sites belonged to the same gene. The green spots

are genes that also contain lineage-specific varied sites in golden pheasant. The orange spots are lipid related genes. **(d)** The theoretical process of carotenoids transportation and deposition. **(e)** The KEGG pathway enrichment of the union DEGs between feather follicles of the two pheasants. The “RichFactor” = the number of DEGs in this pathway/the number of gene set in this pathway. More details are described in Additional file 3: Notes 4.2. **(f)** The expression of the *APOA*1 and *BCO*2 gene in the two pheasants.

Click here to access/download
Supplementary Material
revised-Additional file 1 Figures.docx

Click here to access/download
Supplementary Material
revised-Additional file 3 Notes.docx

Click here to access/download
Supplementary Material
revised-Additional file 2 Tables.xls
