# Supplementary material for: Comparative genomics and transcriptomics of Chrysolophus provide insights into the evolution of complex plumage coloration
Source: Gigascience. 2018 Sep 6;7(10):giy113. doi: 10.1093/gigascience/giy113 (PMC6204425; doi:10.1093/gigascience/giy113)
Supplement: Supplemental Files [file giy113_supplemental_files.zip › revised-Additional file 1 Figures.docx]

**Additional file 1：Supplementary Figures (****Figure S1-S20)**


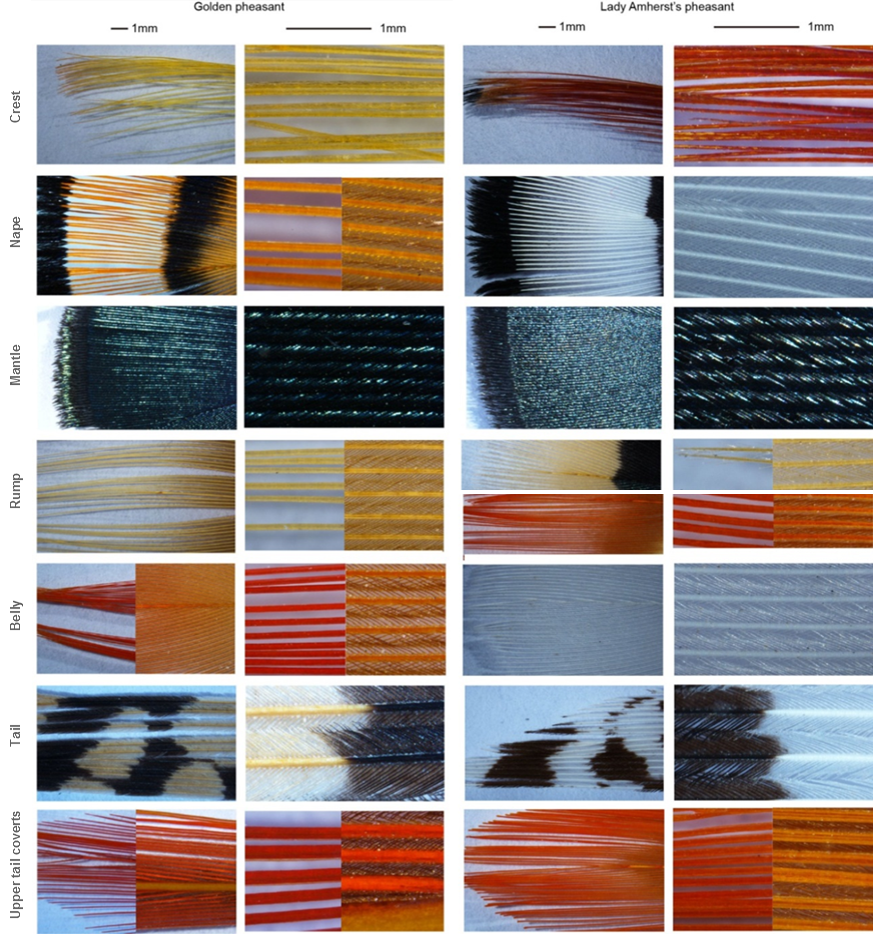


**Figure S1** **Photographs of** ***Chrysolophus* feathers distal parts.** Feathers vary in formation of barbules in the open pennaceous portion. Pictures separated in two parts show the distal and proximal parts, respectively.


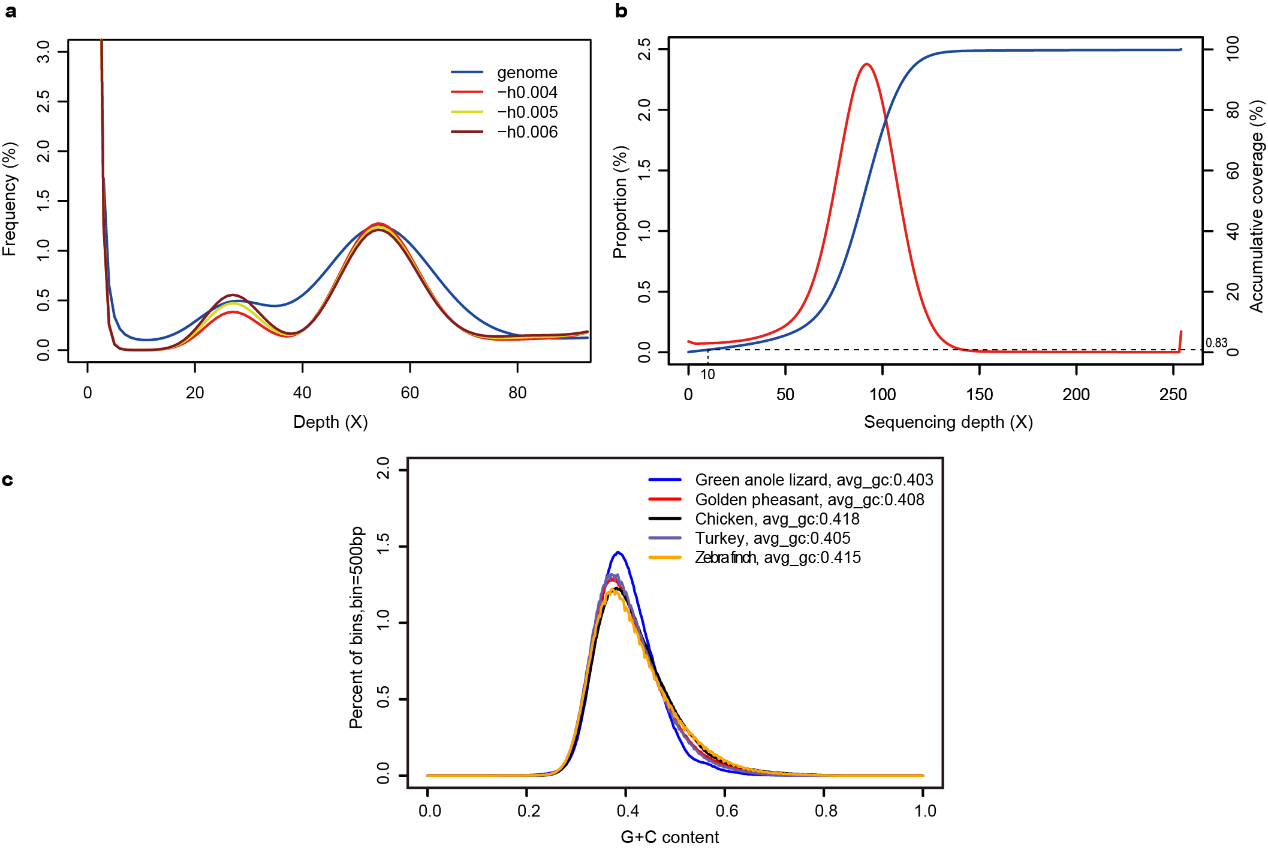


**Figure S2** **Genome assembly of golden pheasant.** **(a)** Genome heterozygosity simulation. The x-axis shows kmer depth and the y-axis shows k-mer frequency. Legends of h0.004, h0.005 and h0.006 mean the heterozygosis rates as 0.4%, 0.5% and 0.6%, respectively. **(b)** Sequencing depth distribution. The x-axis shows sequencing depth of bases. The left y-axis shows the proportion of corresponding genome base sequencing depth (red) and the right y-axis shows the accumulative proportions (blue). **(c)** A comparison of the GC contents of five species. The x-axis indicates GC content and the y-axis indicates the proportion of the bin number divided by the total windows.


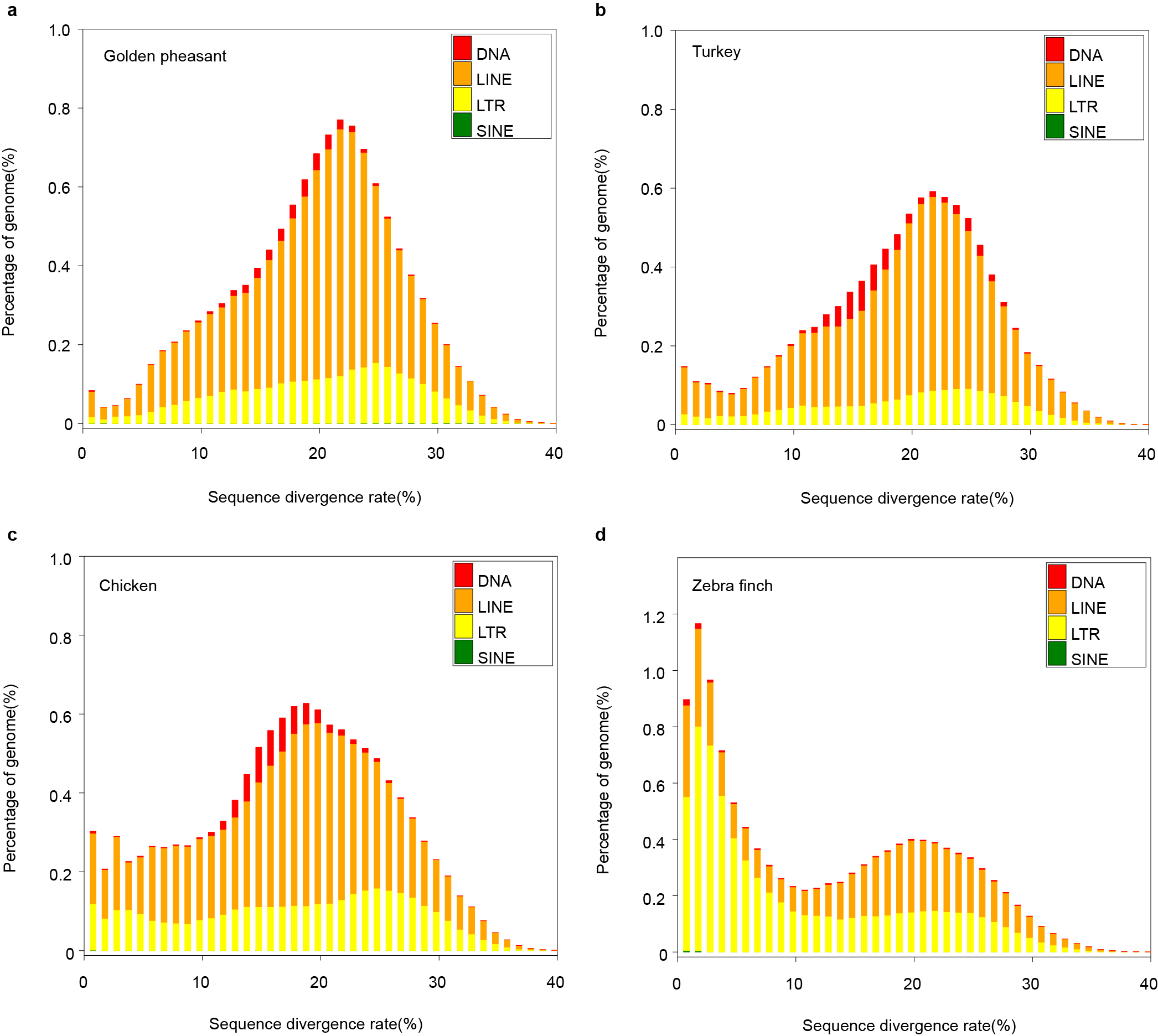


**Figure S3** **Divergence distribution of classified** **transposable element families.** Genomic portion of repeat at different given divergence is from RepeatMasker which is against the *de novo* library of each birds respectively.

.


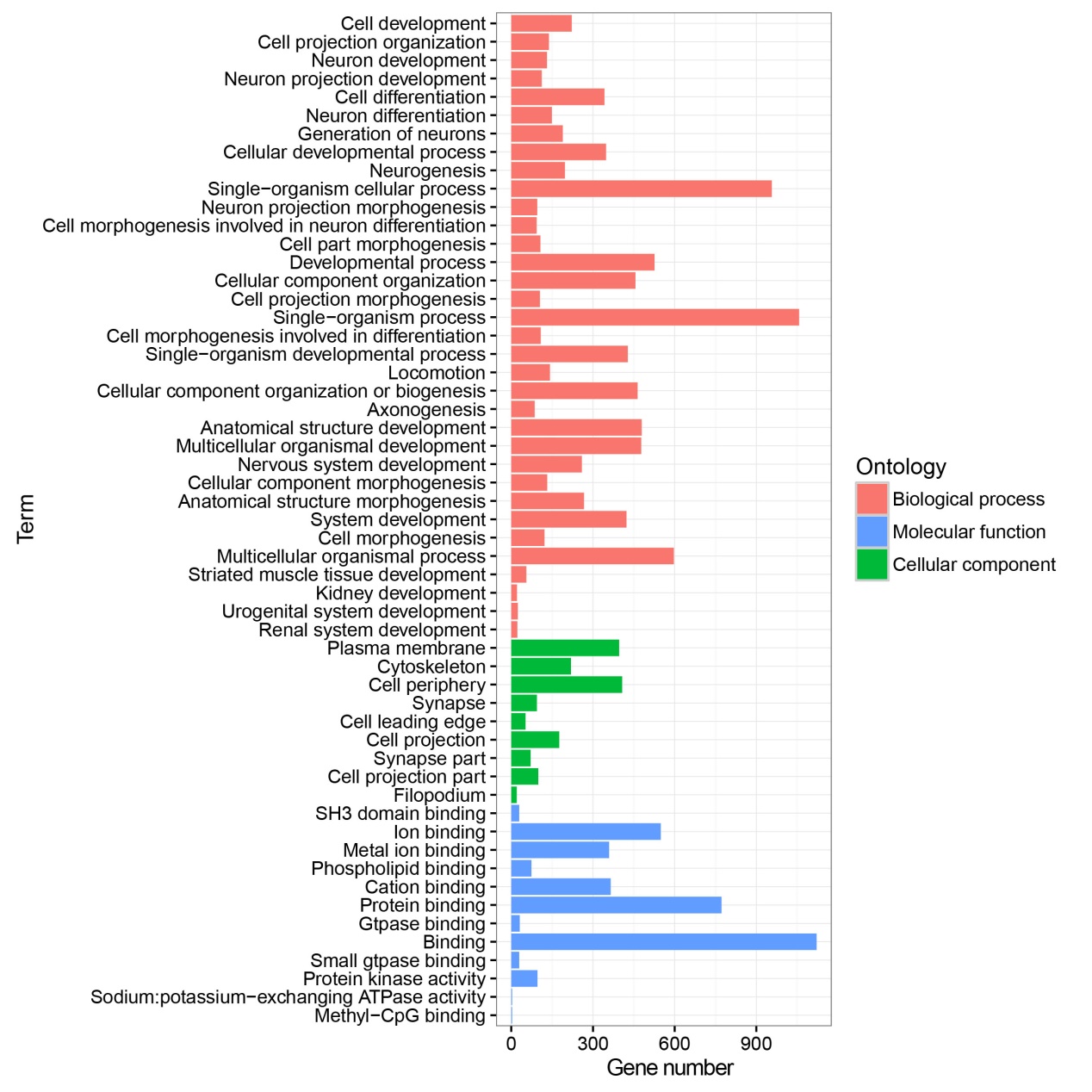


**Figure S4 GO enrichment of flanking genes within 2kb up/downstream to the DNA/CMC, DNA/MULE, and Satellites.**


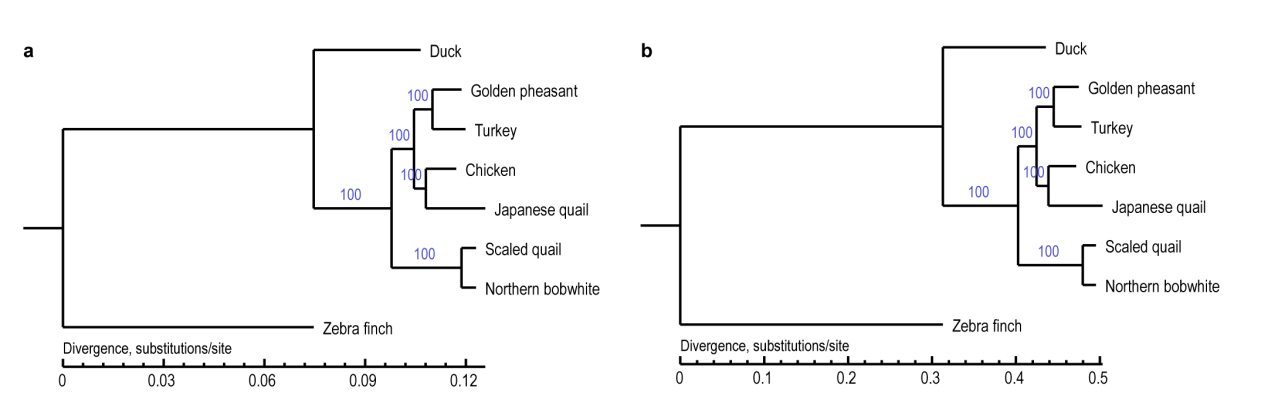


**Figure S5 The phylogenetic analysis of golden pheasant and four other birds. (a)** The maximum likelihood phylogeny tree of five birds base on CDS sequences. **(b)** The maximum likelihood phylogeny tree of five birds based on 4D sites.


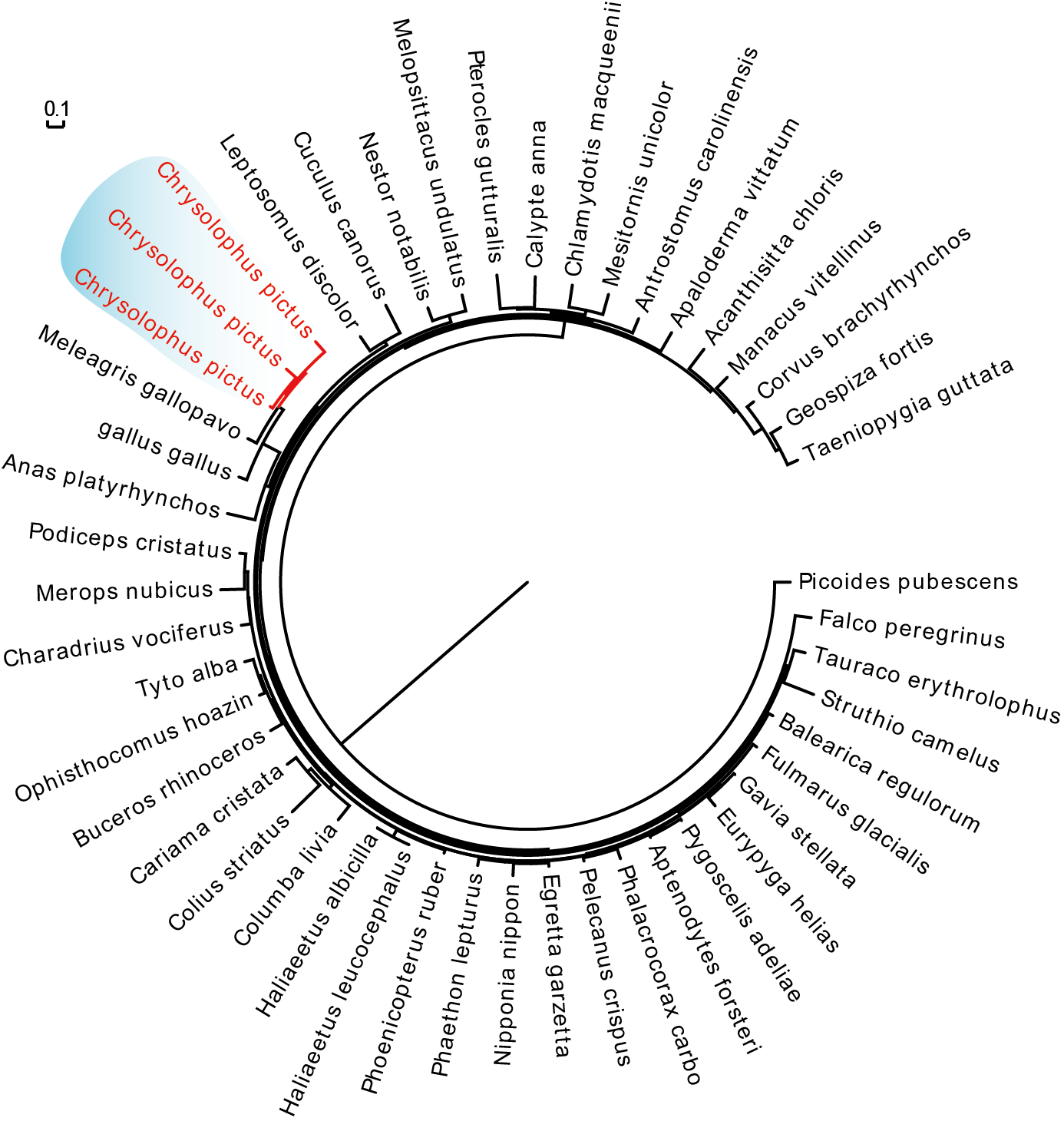


**Figure S6 ML phylogenetic tree of protein sequences of CYP2D6 genes in golden pheasant and other 48 birds.** The expanded cytochrome P450 family gains two more CYP2D copies in golden pheasant.


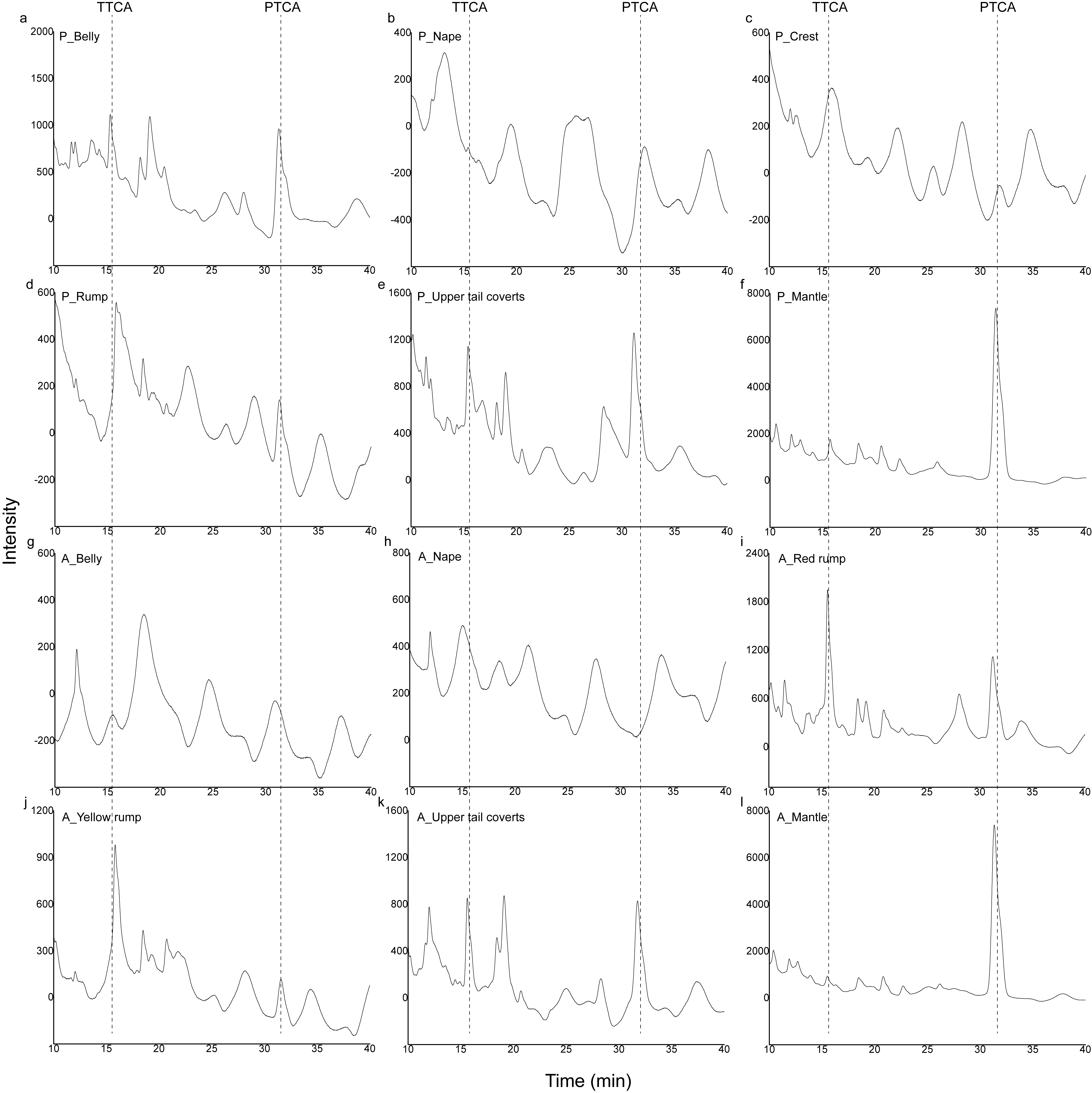


**Figure S7** **HPLC for pigment identification in different feathers.** **(a)** Photograph of feather samples treated with the thermochemical method. The upper coloured phases are caused by carotenoids which are transferred to hexane: TBME from aqueous pyridine. Red and yellow rump feathers remain the initial colour after extraction, and the aqueous pyridine phases are still colourful in these samples. Negative-control tube contained no feathers. **(b)** Reversed-phase HPLC chromatograms of lutein and zeaxanthin from different feathers. P, golden pheasant; A, Lady Amherst’s pheasant. **(c)** Reversed-phase HPLC chromatograms of TTCA and PTCA from different feather.

**
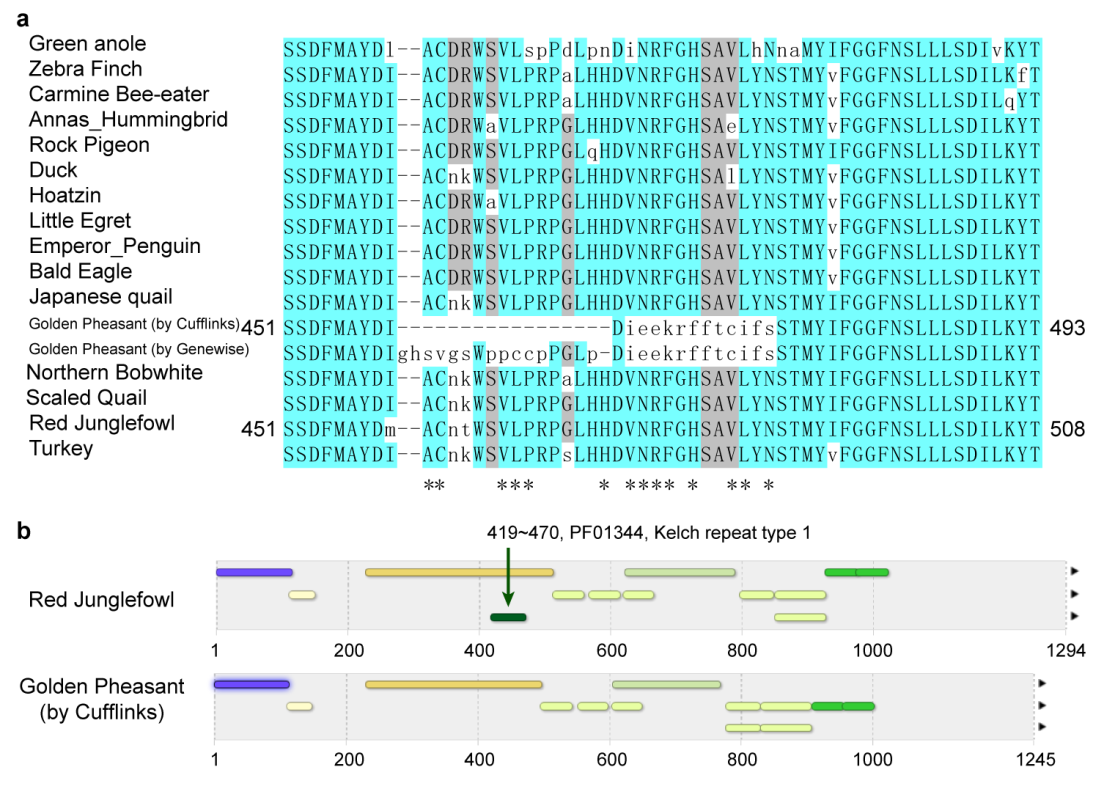
Figure S8 Lineage-specific variations of ATRN gene in *Chrysolophus*.** **(a)** The alignment of protein sequences between golden pheasant and other species. Two strategies of protein sequence alignment (by Genewise) and RNA-based prediction (by Cufflinks) support the variation. The asterisks marked at the bottom indicate the sites under positive selection (BEB test, P > 0.98) in *Chrysolophus*. **(b)** The domain prediction of ATRN protein sequences of chicken and golden pheasant.

**
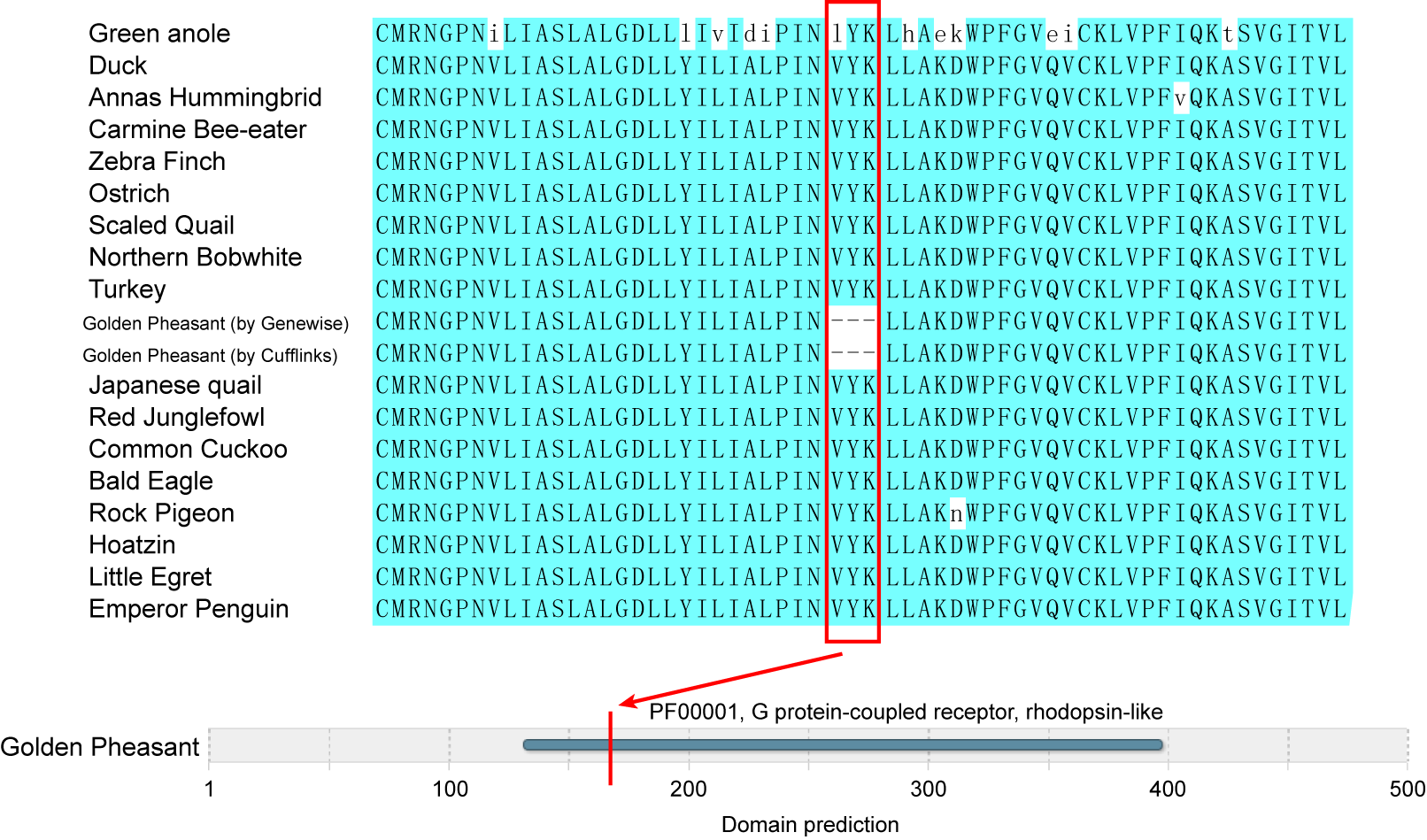
**

**Fig S9 Lineage-specific amino acid deletions of EDNRB gene in** *Chrysolophus.* **(a)** The alignment of protein sequences between golden pheasant and other species. Two strategies of protein sequence alignment (by Genewise) and RNA-based prediction (by Cufflinks) support the deletion. **(b)** The domain prediction of EDNRB protein sequences of chicken and golden pheasant.


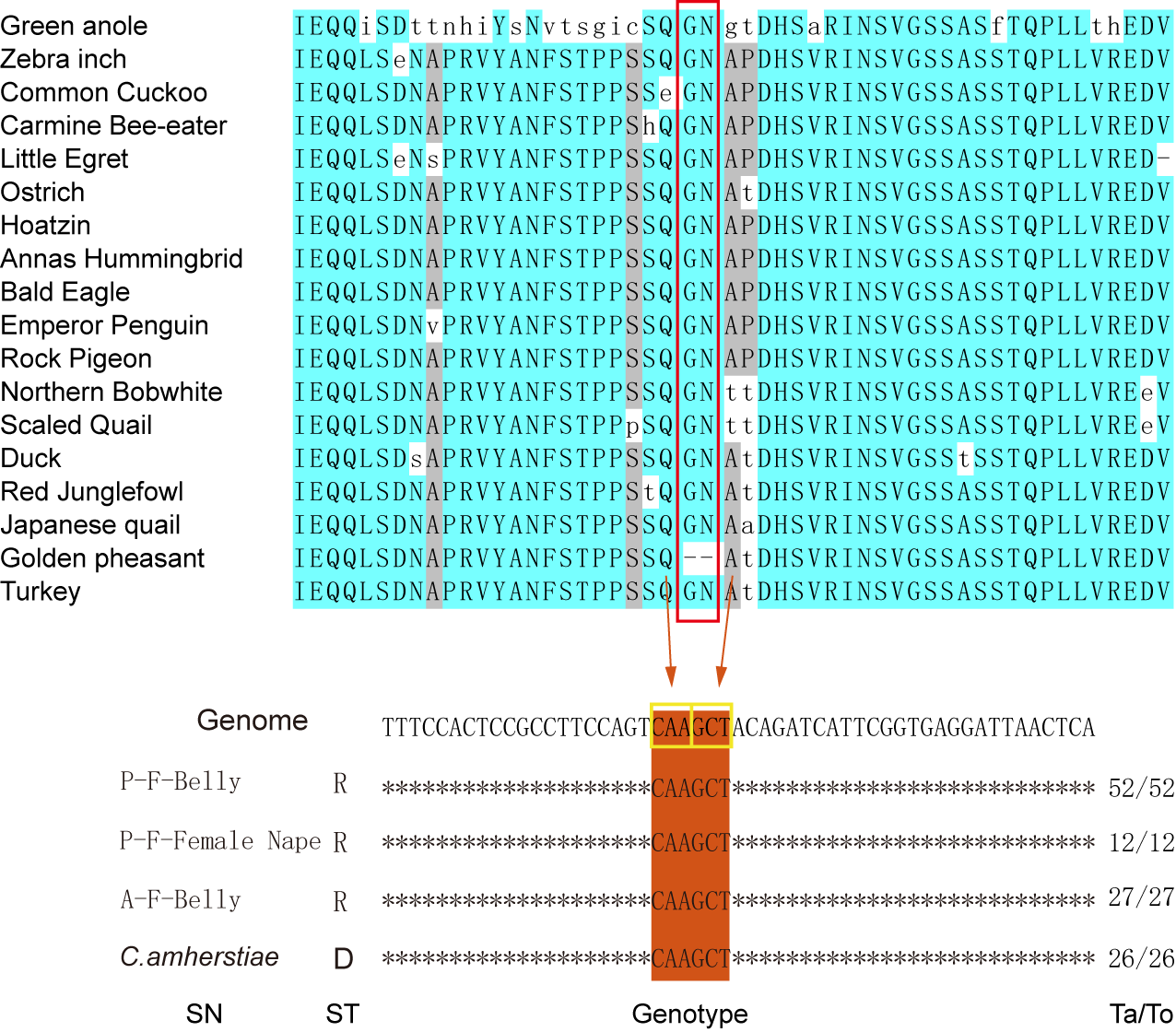


**Figure S10 Comparison of C-terminal of KIT protein-coding genes.** The upper part is the alignment of protein sequences among golden pheasant and other species. It shows that a specific amino acids deletion in golden pheasant which is conserved in other 13 birds and green anole. This deletion was verified in another four *Chrysolophus* individuals (lower part). “SN”, sample name; “ST”, sequencing type, R, RNA sequencing, D, DNA sequencing; “Ta/To”, the number of reads support the shown genotype/the number of total mapped reads. Number of reads supporting and mapping to the loci point are same imply that this deletion is homozygous.


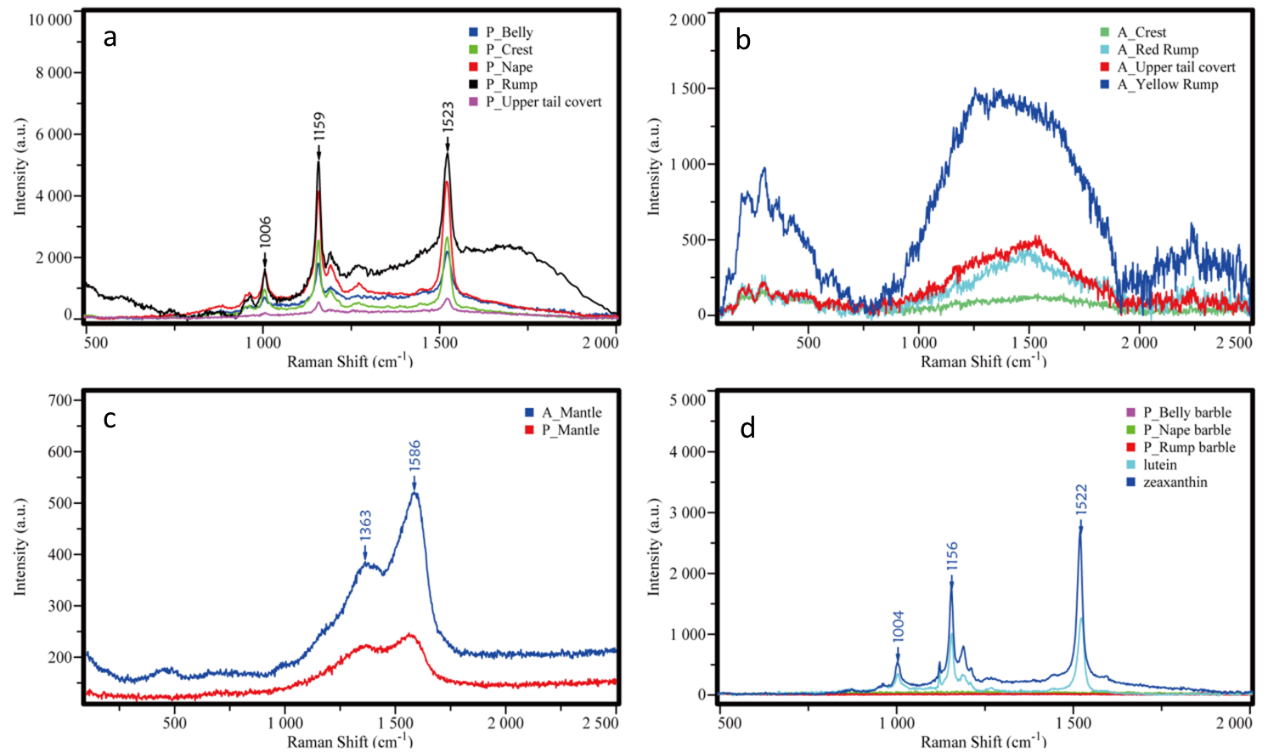


**Figure S11** **Raman spectra for pigment identification in different feathers.** **(a)** Carotenoids in male golden pheasant feathers. **(b)** Pheomelanin but not carotenoid in male Lady Amherst's pheasant feathers. **(c)** Eumelanin in black mantle feathers from male golden pheasant feathers and Lady Amherst's pheasant. **(d)** Absence of carotenoids in the barbules of golden pheasant yellow to red feathers. P: golden pheasant; A: Lady Amherst's pheasant.


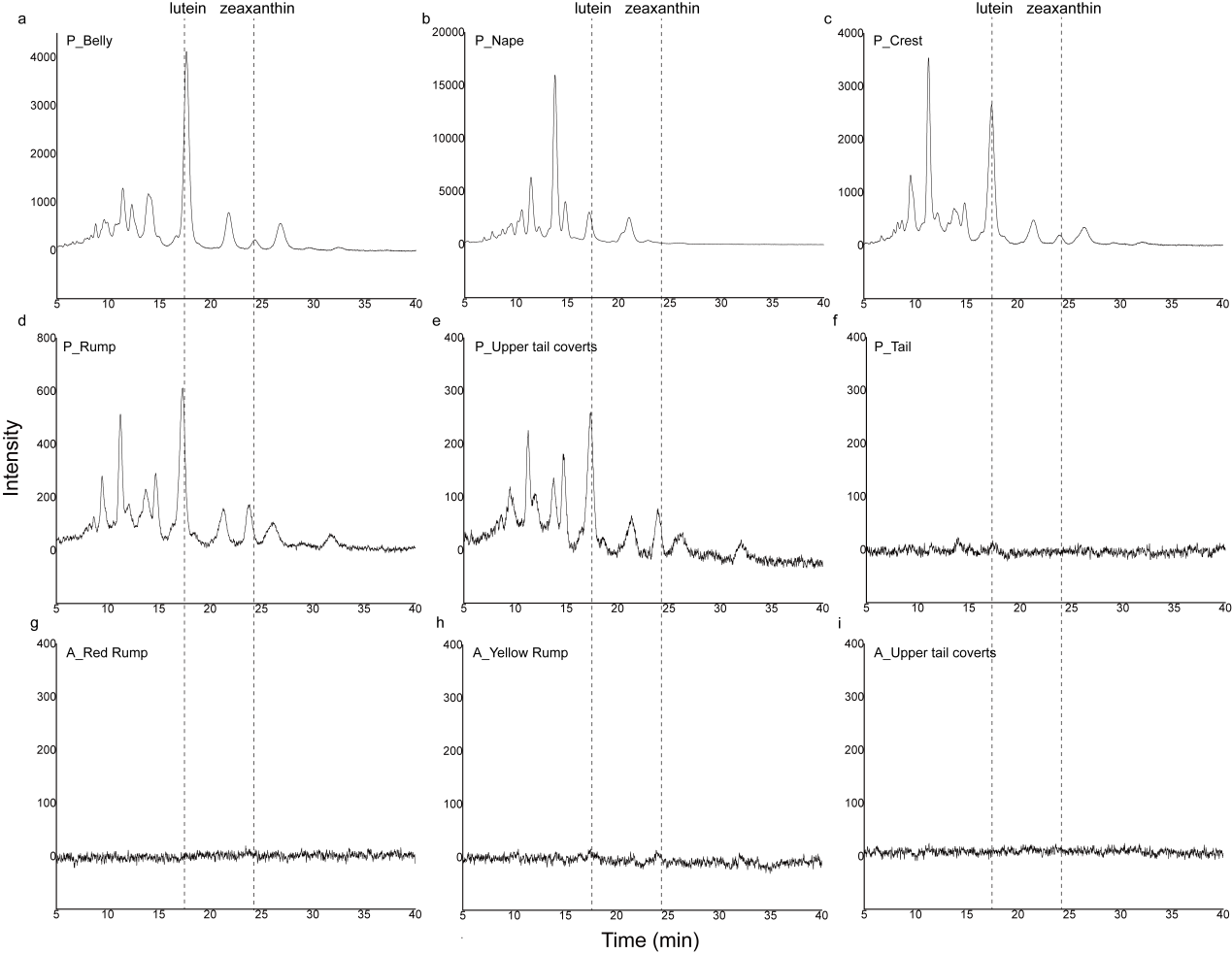


**Figure S12** **HPLC for pigment identification in different feathers.** **(a)** Photograph of feather samples treated with the thermochemical method. The upper coloured phases are caused by carotenoids which are transferred to hexane: TBME from aqueous pyridine. Red and yellow rump feathers remain the initial colour after extraction, and the aqueous pyridine phases are still colourful in these samples. Negative-control tube contained no feathers. **(b)** Reversed-phase HPLC chromatograms of lutein and zeaxanthin from different feathers. P, *pictus*; A, *amherstiae*.


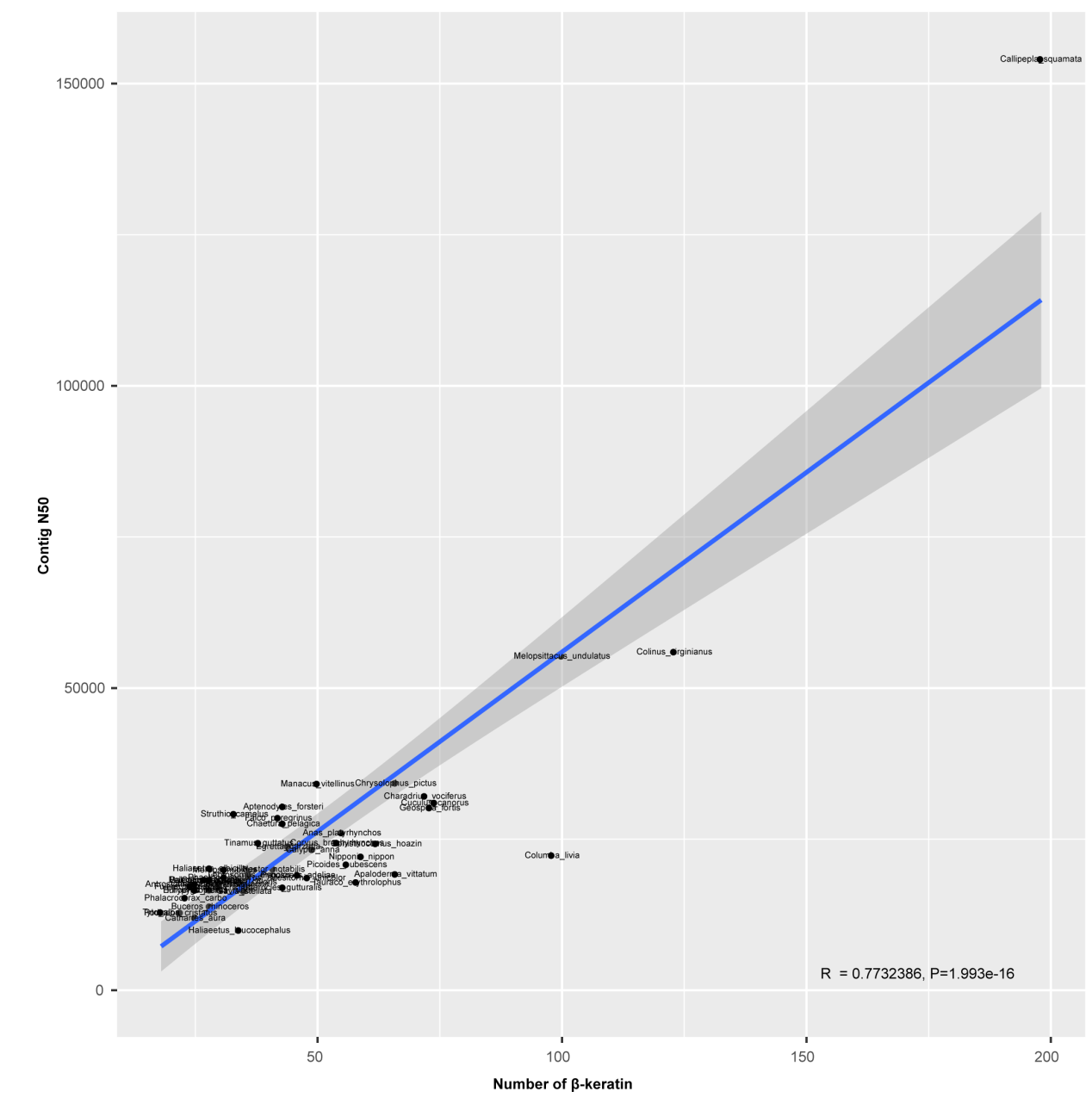


**Figure S13 The correlation between contig N50 and number of predicted β-keratin genes within golden pheasant and other 45 birds.** The prediction of β-keratin of all these birds are using a same pipeline (tblastn + solar + genewise) against the same database. The chicken, turkey, and zebra finch are not included because of sequencing by Sanger technology. The fit line was derived from least square regression analysis, and the confidence interval was estimated using “stat_smooth” in R.


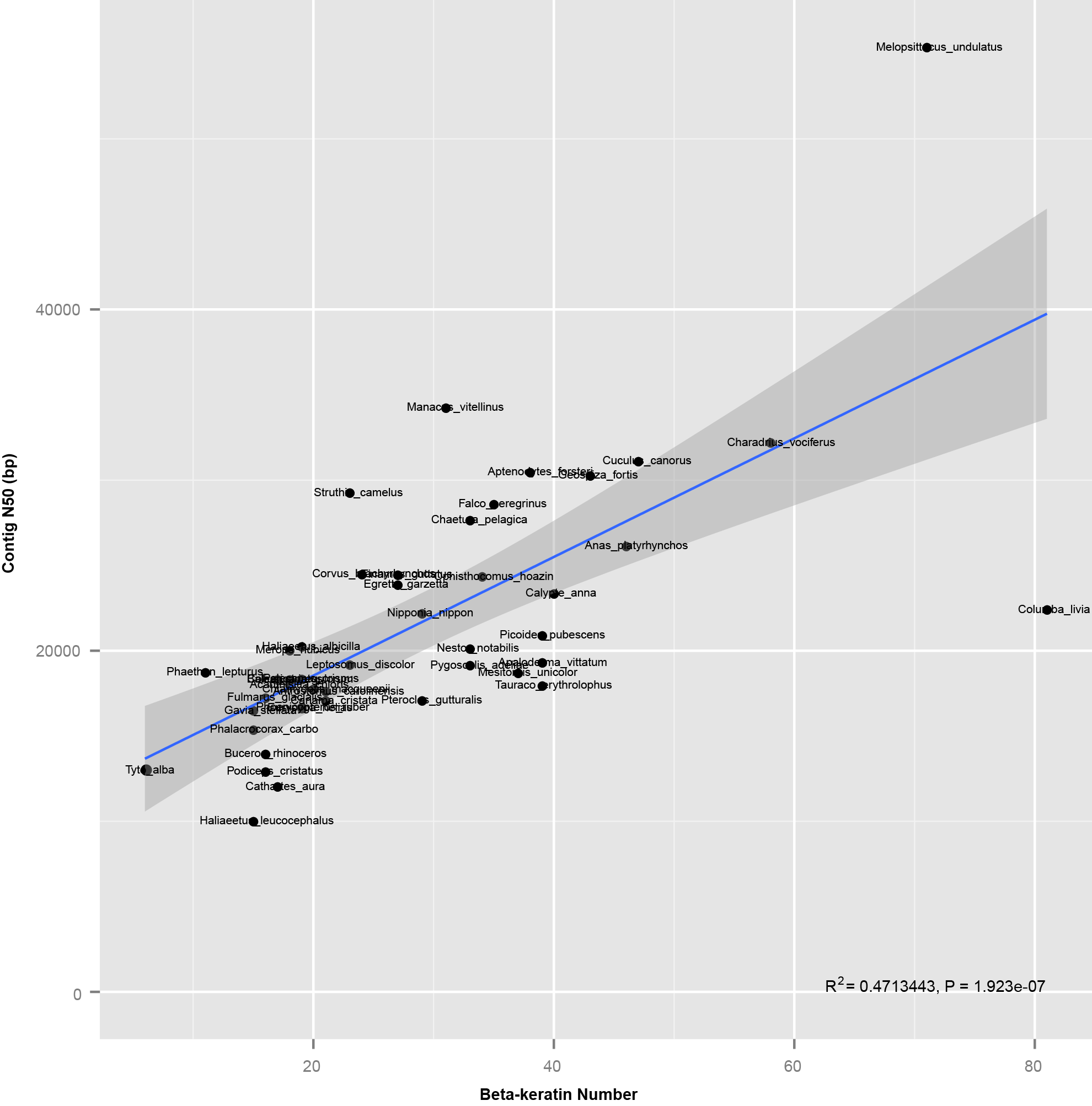


**Figure S14 The correlation between contig N50 and number of completed β-keratin genes within 45 sequenced birds.** The completed β-keratins of the 45 birds are from Greenwold’s study [1]. The chicken, turkey, and zebra finch are not included because of sequencing by Sanger technology. The fit line was derived from least square regression analysis, and the confidence interval was estimated using “stat_smooth” in R.


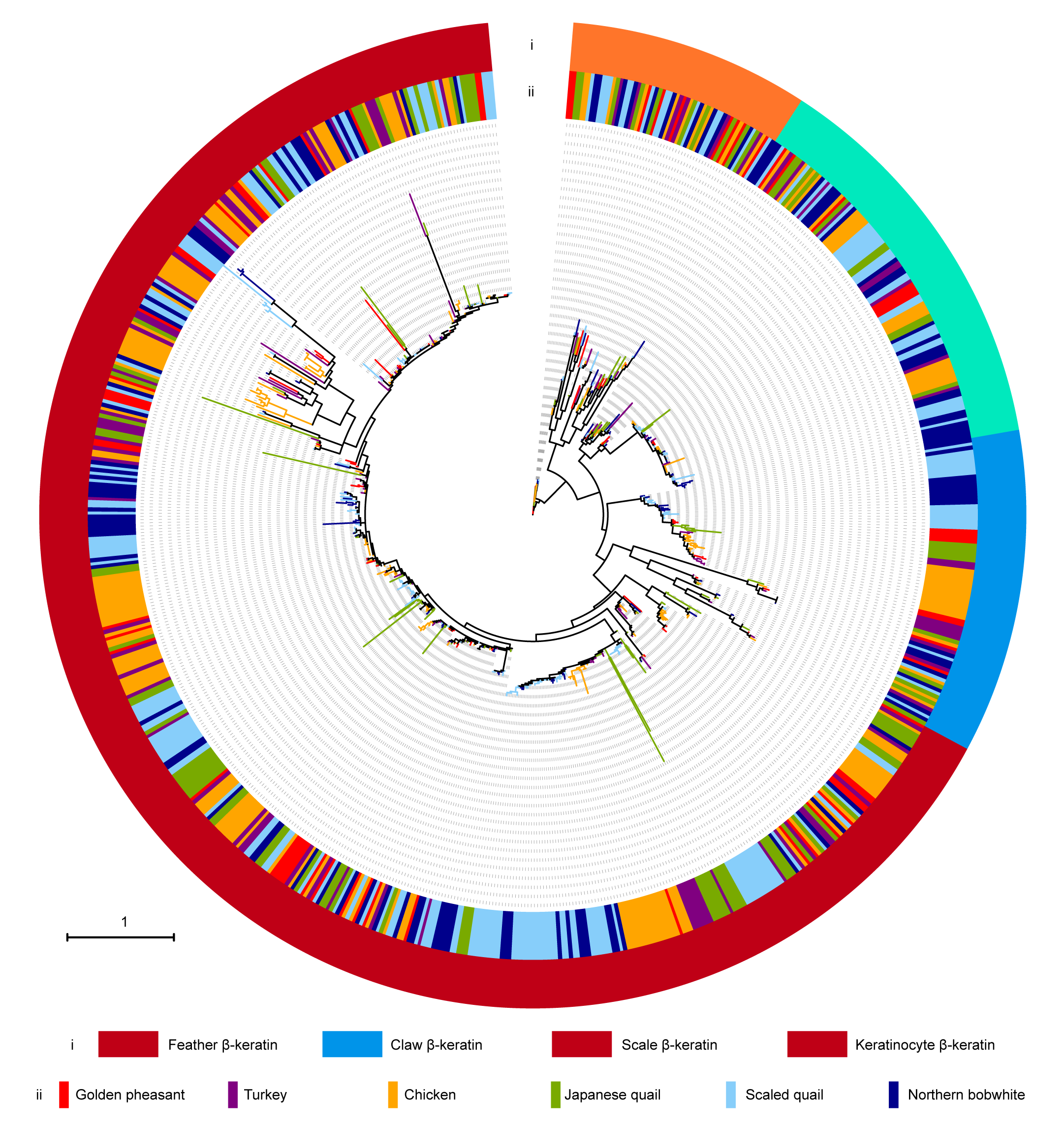


**Figure S15 The phylogeny tree of β-keratin genes in 6 birds of Galliformes.** The tree was constructed using Raxml with parameter “-p 12345 -m PROTGAMMAAUTO -x 12345 -f a -# 100 –o “keratinocyte β-keratins”. The “keratinocyte β-keratin” was the gene ID of all keratinocyte β-keratins in six avian species connected by commas. Using keratinocyte β-keratin as root was based on the Greenwold’s study [1].


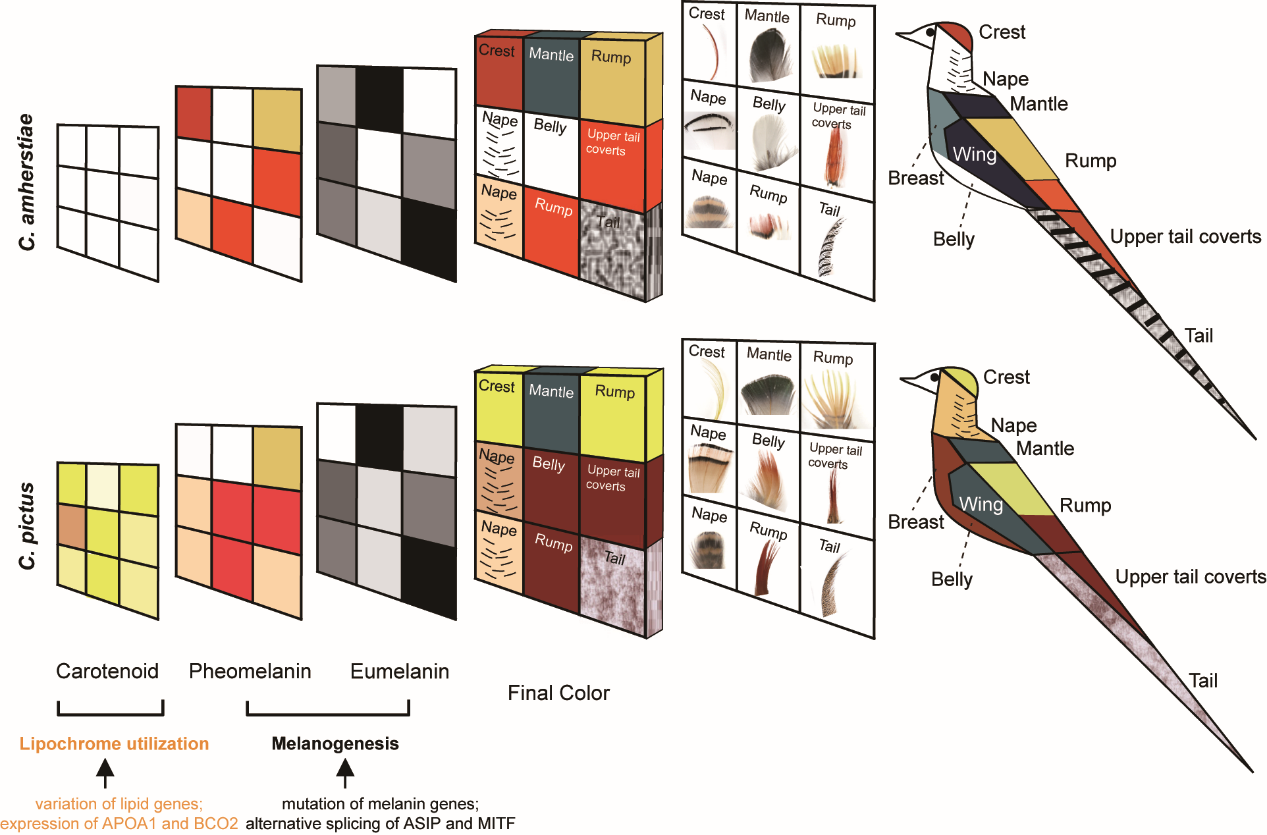


**Figure S16 A suggested combinatorial model for complex colouring in *Chrysolophus*.** This schematic process is a collection of filters that one looks through to assemble the final colour in each feather in Lady Amherst's pheasant (top) and the golden pheasant (bottom). Each of the 9 squares represents a different feather tract.


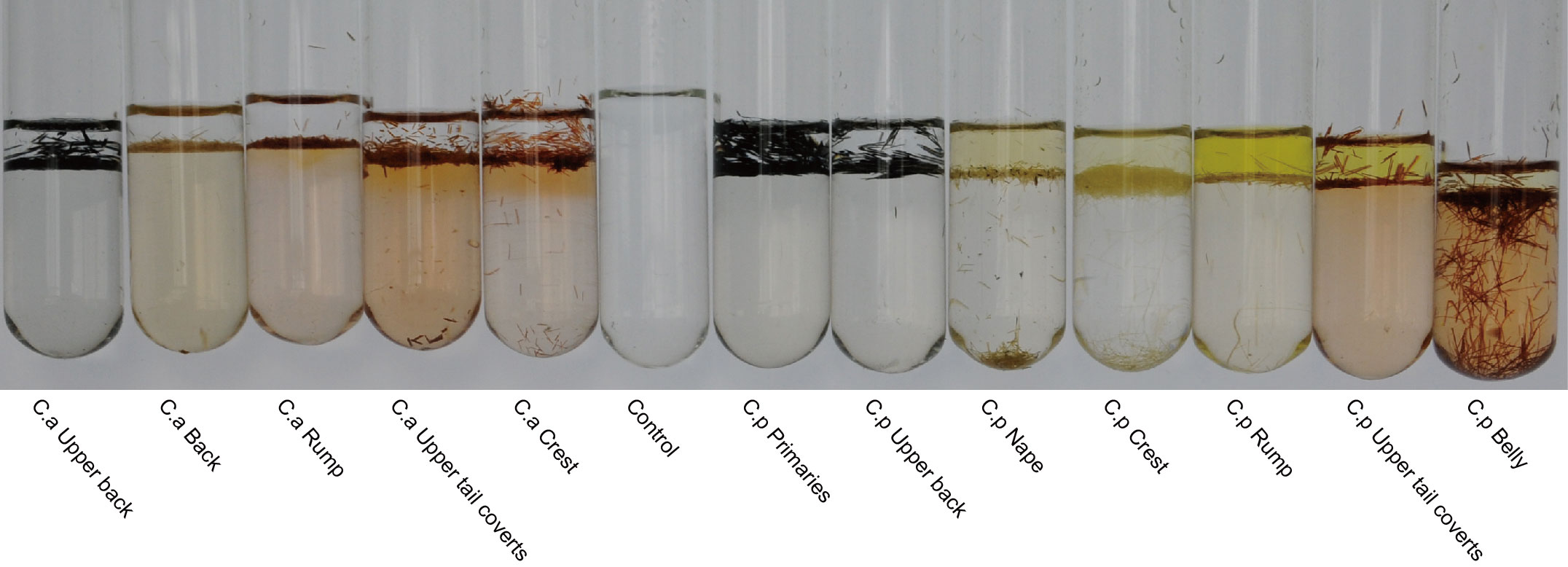


**Figure S17 Photograph of feather samples treated with the thermochemical method.** The upper coloured phases are caused by carotenoids which are transferred to hexane: TBME from aqueous pyridine. Red and yellow rump feathers remain the initial colour after extraction, and the aqueous pyridine phases are still colourful in these samples. Negative-control tube contained no feathers.


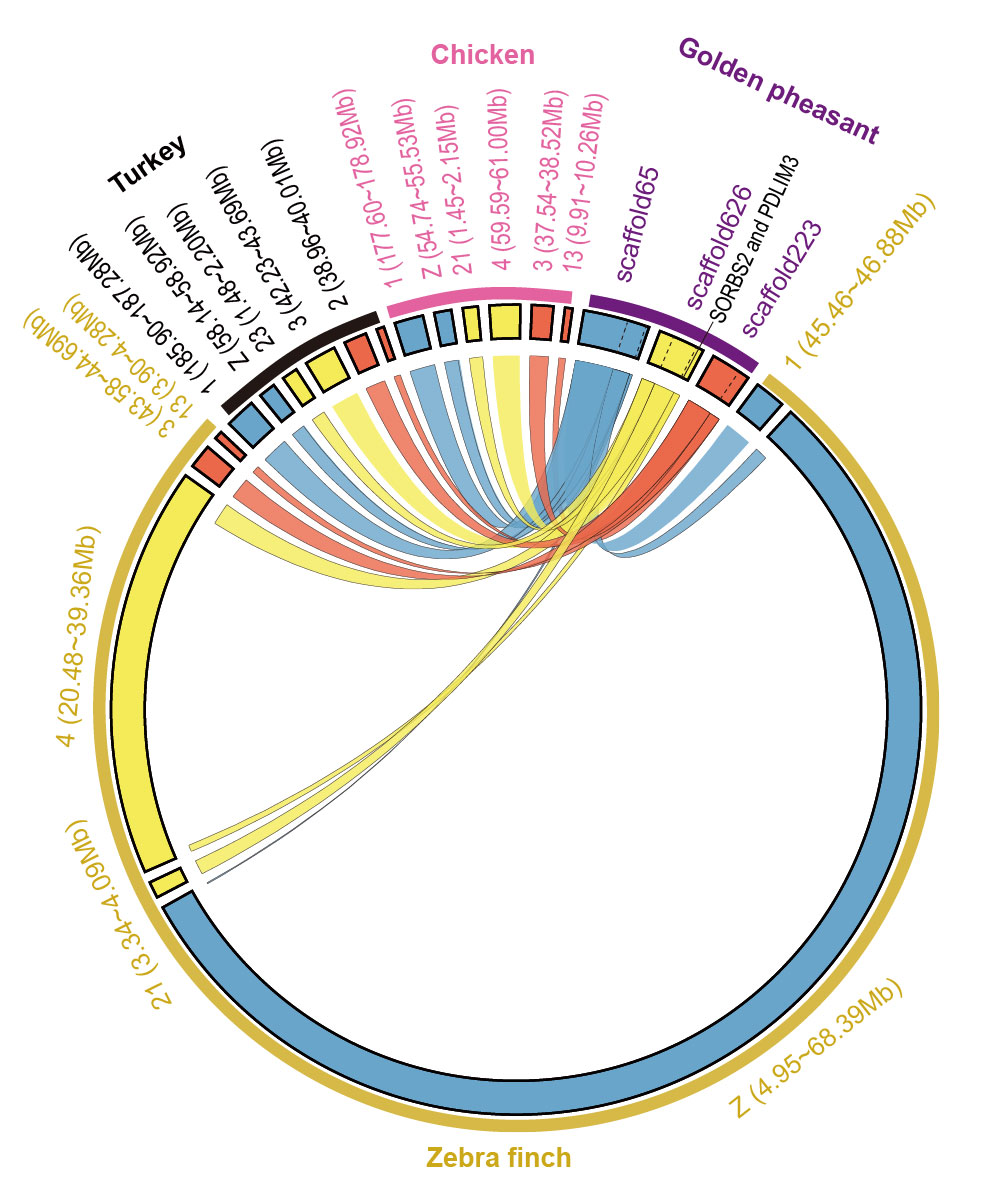


**Figure S18 Chromosomal rearrangement in three large blocks between the golden pheasant and other species.** Solid curves on the outside indicate the different species. Bars in the middle indicate chromosomes/scaffolds. The dashed lines represent the breakpoints.


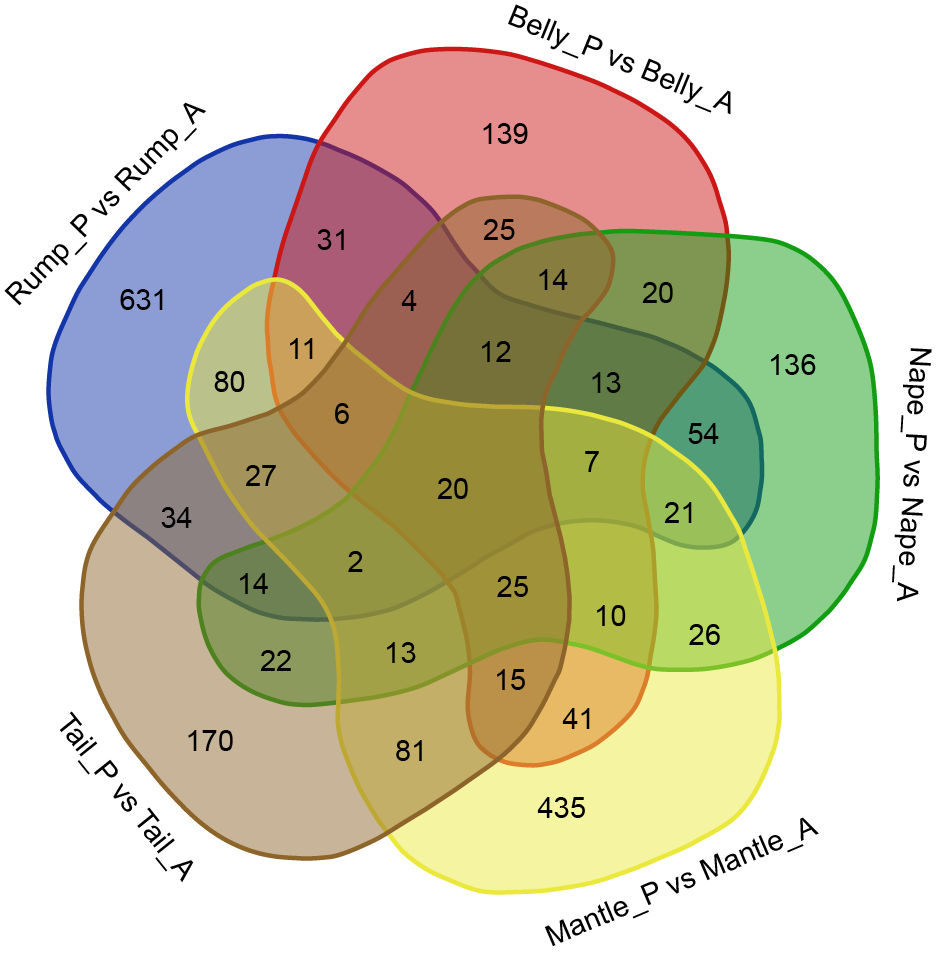


**Figure S19 Venn diagram of differentially expressed genes overlapped by five groups.** Each group of DEGs is calculated in feathers from the same body part of golden pheasant (P) and Lady Amherst’s pheasant (A). More details are described in Supplementary Notes 4.2.


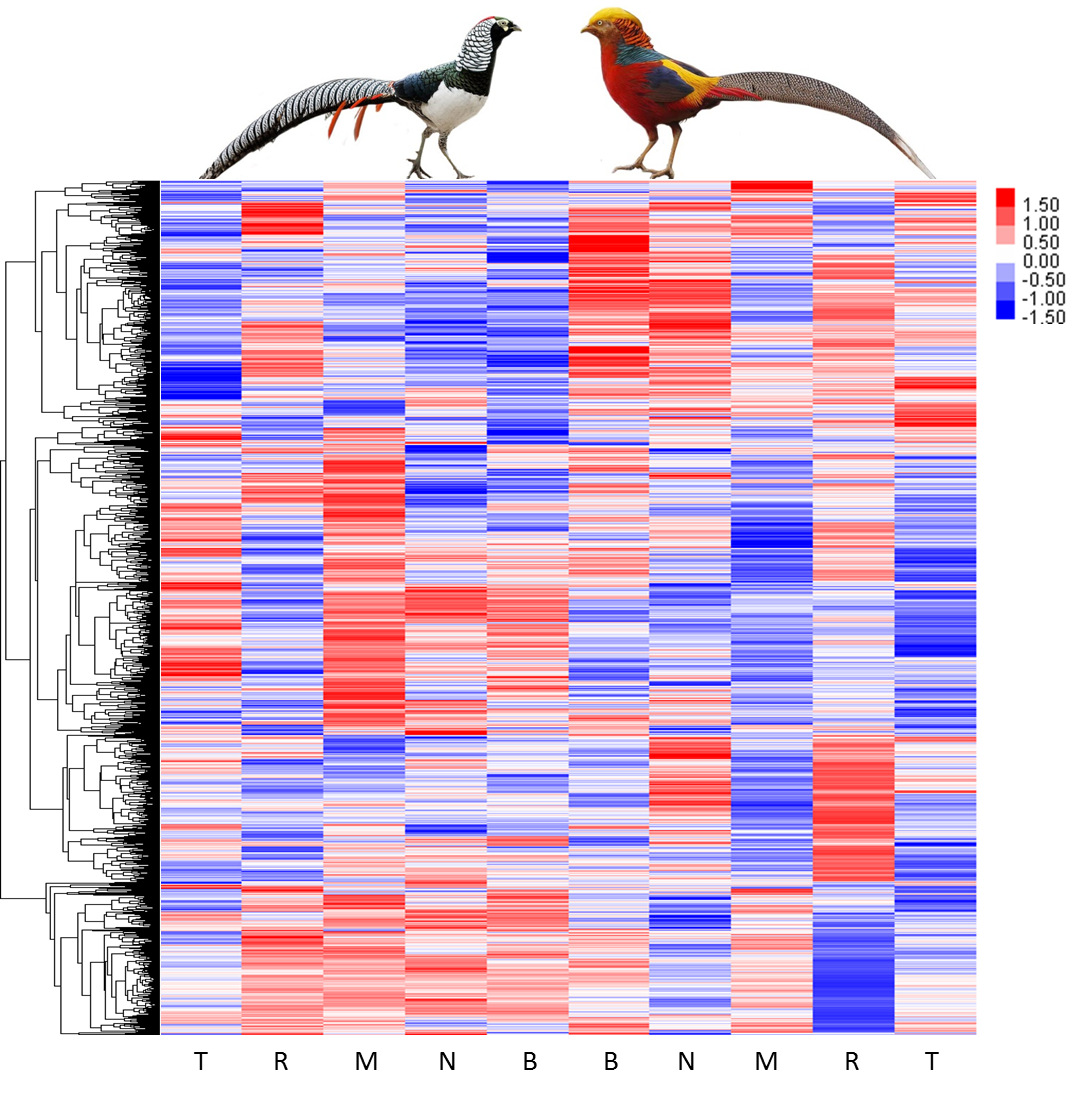


**Figure S20 Gene-wise hierarchical clustering heat map of all different expressed genes between golden pheasant and Lady Amherst’s pheasant feathers.** All DEGs are combined by each parallel compared group of two species. The bold capital letters indicate feather samples from different body parts (T: tail, R: rump, M: mantle, N: nape, B: belly). More details are described in Supplementary Notes 4.2.

1. Greenwold MJ, Bao W, Jarvis ED, Hu H, Li C, Gilbert MT, Zhang G, Sawyer RH: **Dynamic evolution of the alpha (alpha) and beta (beta) keratins has accompanied integument diversification and the adaptation of birds into novel lifestyles.** *BMC Evol Biol* 2014, **14:**249.
