# Supplementary material for: Comparative genomics and transcriptomics of Chrysolophus provide insights into the evolution of complex plumage coloration
Source: Gigascience. 2018 Sep 6;7(10):giy113. doi: 10.1093/gigascience/giy113 (PMC6204425; doi:10.1093/gigascience/giy113)
Supplement: Supplemental Files [file giy113_supplemental_files.zip › revised-Additional file 3 Notes.docx]

**Additional file 3: Supplementary Notes**

**1.** **Plumage pigments identification**

The most common pigments of plumage are melanins and carotenoids. In animals, melanins are genetic synthetized in melanocytes which yield colour of black, grey, reddish and buff, whereas dietary derived carotenoids exhibit red, orange and yellow hues. McGraw has recorded 24 different carotenoids biochemically characterized from roughly 150 avian species up to 2006 [1], and 6 more novel methoxy-carotenoids were identified from plumage of the Pompadour Cotinga (*Xipholena punicea*) [2].The family-level distribution of plumage carotenoids confirmed their presence in 95 of 236 extant bird families [3].

**1.1 Raman spectroscopy**

Raman spectroscopy as a non-destructive method was used to preliminarily examine pigments contained in feathers from both golden pheasant and Lady Amherst's pheasant. All samples were analyzed in a Labram HR1800 spectrometer (HORIBA JobinYvon, France) which used an excitation laser source at 514 nm of 3 mW or 0.3 mW (black barbs) power. The single spectra were obtained using a 100 × confocal objective and a grating of 600 lines/mm. Data were analyzed by LabSpec 5.

We used Raman spectroscopy for the identifications because this method is effective and non-destructive and prevalently used in pigment determinations [4-6]. From the peaks of Raman shifts, which appeared at 1500-1535 cm^-1^(identified as ν[C=C]), 1145-1165 cm^-1^(identified as ν[C-C]) and 1000-1010 cm^-1^(identified as δ [CH_2_]) [4], we can consider the presence of carotenoid in golden pheasant feathers but not in Lady Amherst's pheasant whose spectra of red or yellow feathers featured pheomelanin instead (Figure S11a and b**)**[6]. Besides, eumelanin can be shown by the Raman bands of black feathers from both species (Figure S11c) [7].

**1.2 High-performance liquid chromatography**

Carotenoid pigments in feathers were extracted using previously published method [8, 9]. Briefly, prior to the thermochemical procedures, each type of coloured feather was soaked in ethanol and hexanes in turn so that surface lipids were removed. Pigmented barbs were trimmed into pieces, weighed and placed into a 10ml glass tube which can be capped tightly, and then 1ml acidified pyridine was infused to cover the pieces. The tube was filled with argon gas and placed in a 368K water bath for 3 h. After cooling to room temperature, 2 ml pure water and 1ml hexane: *tert*-butyl methyl ether (1:1) was added into the tube in order to separate the carotenoid pigments from the solution. The mixture was vigorously shaken for 2 min and centrifuged at 3000 rpm for 5 min. The supernatant was transferred to a clean tube, dried under nitrogen gas, and stored at 193 K for subsequent analysis. HPLC analysis was used to identify and quantify carotenoids. Dried pigment was dissolved in HPLC mobile phase (methanol/acetonitrile, 50:50 v/v, +0.05% triethylamine), filtered through a nylon syringe filter and injected into an SIL-20A HPLC system (Shimadzu, Japan) equipped with a YMC C-30 Carotenoid column (5 μm, 4.6 mm × 250 mm; YMC, Japan) and an SPD-20A UV/Vis detector (Shimadzu, Japan). An isocratic system at a flow rate of 1.5 ml/min for 40 min was programmed and data were collected at 450 nm [4, 8].

Melanin pigments were characterized as previously described [10, 11]. The resulting oxidation products of both eumelanin (pyrrole-2,3,5-tricarboxylicacid, PTCA) and pheomelanin (thiazole-2,4,5-tricarboxylic acid, TTCA) were analyzed via HPLC. Feather samples were first treated under alkaline H_2_O_2_ conditions. In brief, 20 mg barbs were weighed and ground into powder in liquid nitrogen. After warming to room temperature, 2 ml pure water was infused to the powder and 200 μl suspensions were placed into a 10ml glass tube, to which 750 μl 1 mol/ l K_2_CO_3_ and 50 μl 30% H_2_O_2_ were added. After the mixtures were shaken at 298K for 20 h, 100 μl 10% Na_2_SO_3_ and 280 μl 6 mol/l HCl were used to decompose the residual H_2_O_2_ and acidify the solution, respectively. The reaction products then were centrifuged at 4000 g for 1 min and an aliquot of each supernatant was injected into the same HPLC system except equipped with a Wondasil C18 column (5 μm, 4.6 mm × 250 mm; GL Sciences, Japan). TTCA and PTCA were analyzed with a mobile phase of 0.1 mol/l potassium phosphate buffer (pH 2.1) / methanol, 99:1 (v/v) at 318K at a flow rate of 0.7 ml/ min, and data were collected at 269 nm.

Pigments were identified by comparing the retention times (tR) to standards of Lutein (Sigma, X6250-1MG) and zeaxanthin (ChromaDex, ASB-00026505-005), while TTCA and PTCA were provided by Dr. Kazumasa Wakamatsu (Fujita Health University School of Health Sciences, Japan). The examined compounds were quantified using standard curves. HPLC data were analyzed with OriginPro 9.0.

Further analysis of carotenoids was needed for identification and qualification by HPLC. Two common dietary carotenoids, lutein and zeaxanthin were used as standards in our study. Data showed that both chemicals did exist in the distal part of yellow to red golden pheasant feathers but were absent in Lady Amherst's pheasant feathers (Figure S12).

During the process of carotenoid extraction, we found the red golden pheasant belly feathers remained the initial colour even after the thermochemical procedures, and the aqueous phase was still coloured even after treatment with multiple extractions (Figure S17). The phenomenon also appeared in rump yellow feathers. It is possible that two types of pigment confer colours on these tissues [9]. Therefore, we suggested that pheomelanin mainly produced red or yellow colours in the corresponding feathers because these two colours were not exclusively carotenoid-based [12]. On the contrary, aqueous phases of crest golden feathers and nape orange feathers were colourless after extractions, and the once-pigmented tissues nearly lost their colour entirely, except the black stripes of nape feathers. This indicates that carotenoids should be the only pigment in these feathers. Notably, the hexane: TBME phase of nape samples were orange in colour whereas others were yellow including those extracted from red feathers. This orange pigment may be due to other carotenoids rather than lutein or zeaxanthin.

It is known that the ratio of TTCA ⁄PTCA can be used to estimate the relative contents of pheomelaninin mixed melanogenesis. PTCA measures the DHICA- eumelanin units while TTCA examines benzothiazole units derived from a late stage of pheomelanin production [10, 13]. The result of eu- /pheomelanin analysis by HPLC indicated that the ratio of TTCA ⁄ PTCA was the lowest in black mantle feathers, moderate in reddish feathers and highest in light yellow rump feathers (Table S14).

**2. Animal and sample**

**2.1 Animal husbandry**

A total of 6 birds were used in this study, including 3 male and 1 female golden pheasants and 2 Lady Amherst's pheasants, which were provided by Foping wild life protection station (Foping National Nature Reserve, Shaanxi province, China) from 2011 to 2013, and housed in Yuanfeng wild animal farm (Jilin province, China). Male pheasants were focused on the analysis of genome/transcriptome and plumage colouration, while the female individual was used as a negative control.

After the pheasants obtained, they were housed in cages and free access to water and commercial food, including grains, protein-rich foods, vegetables and fruits.

**2.2 Tissue sampling**

Feather follicles and other integument or internal tissues were sampled from two years old pheasants in the gene expression study. For the feather follicles sampling, we used the previously published method [14], briefly, we plucked their feathers during natural molting, then allowed feathers to grow for two-four weeks, at which stage the previously plucked areas of skin contained densely spaced feather shafts with the first parts of feathers about to or just protruding from the shafts. Each sample contained 5-10 feather follicles, 4 samples (nape, belly, mantle and rump) were from one male golden pheasant and 1 sample (tail) were from another individual, while the equivalent samples were obtained from two Lady Amherst's pheasants ; in addition, we collected nape feather follicles from one adult female golden pheasant. For the skin tissues, we sacrificed one male golden pheasant to gain 5 samples of skin tissues with growing feather follicles (crest, nape, belly, mantle and rump). For internal tissues, 4 samples (brain, liver, heart, and muscle) were donated by the same sacrificed golden pheasant. All samples were submerged into liquid nitrogen immediately and then stored at 213K.

**3. Genome rearrangements**

We identified the syntenic blocks between golden pheasant and chicken, golden pheasant and turkey respectively based on the orthologous gene pairs. We aligned protein sequences of the two species to each other by BLASTP and combined HSPs with the Solar firstly. Then we identified reciprocal best hit (RBH) as orthologous gene pairs. Thirdly, we placed best-hit gene pairs on their chromosomes/scaffolds according to golden pheasant, and sorted them in order. We identified that it was a chromosome structure variation if there is conflict in the order between the two species. If the golden pheasant was different with both chicken and turkey and the chicken is same with turkey, we identified that it was a golden pheasant specific chromosome rearrangement variation.

Chromosomal rearrangement is one of the important mechanisms for genome evolution [15]. We compared genome synteny among Galliformes by aligning the orthologous gene pairs. We screened the non-syntenic blocks which contained specific gene order in golden pheasant genome and found 41 intrachromosomal 14 and interchromosomal rearrangements (Table S22-24). In the intrachromosomal rearrangements, 15 colour genes and 24 HOX genes were involved, with *NF1*, *TIMP3*, *DOCK7* and *HESX1* localizing adjacent to the breakpoints (Table S22). Of the interchromosomal rearrangements, there are three golden pheasant scaffolds (scaffold 65, scaffold 223, scaffold 626) contain more than ten genes (27, 14, 43 genes, respectively) (Table S24), these scaffolds are in synteny with different chromosomes of both chicken and turkey genomes (Figure S18).

4. **Transcriptome analysis**

**4.1 RNA-seq**

Libraries of different organizations or different colour feathers from golden pheasants and Lady Amherst’s pheasants were constructed using the Illumina TruSeq RNA sample preparation kit according to manufacturer’s instructions. The libraries (insertion size ~200 bp) were sequenced 90 bp at each end using Illumina Hiseq 2000 platform. We achieved 48~83 million reads per library (Table S28). RNA reads were mapped by Tophat and subsequently analyzed with in-house Perl scripts. We quantitated the gene expression level using unique mapped reads and normalized using per kilobase of transcript per million mapped reads (RPKM) [16]. For detecting differentially expressed genes (DEGs) between different individual samples, we used a method described by Chen et al [17]. In this study, we defined DEGs using two criterions: a) RPKM is at least two-fold difference; b) the false discovery rate (FDR) is less than 0.001. For detecting DEGs between different groups which can contain multiple samples, we used Noiseq [18] with cut off Probability ≥ 0.8. For an alternative splicing analysis, RPM (reads per million mapped reads) was used to calculate the expression of the splice site. The differentially expressed junctions are identified by DEGseq[19] with MA-plot-based method with Random Sampling model.

**4.2 Colour-specific expressed genes**

Colour is one of the most studied topics to investigate avian plumage evolution and diversity, however, the colouration mechanisms remain to be fully elucidated [20]. In our study, comparative analyses were performed at a further transcriptome level to investigate the molecular mechanisms of melanin formation and carotenoid deposition in golden pheasant and Lady Amherst’s pheasant. To identify gene expression divergence in feathers with various colours, we compared the transcriptomes of belly, nape, mantle, yellow rump and tail feather follicles between golden pheasant and Lady Amherst’s pheasant. The expressed genes of the five samples in each species were grouped as a whole, representing the entire feathers of species. By comparing the groups we found only 20 genes were differentially expressed (Slc23a1, TYRP1, CXCL8, SST, CXCL8, CCL5, TGM4, APOA1, GJB6, AQP9, TTR, AVR2, gag-pro, Cxcl14, EXFABP, MLKL, cldnd, gag-pro-pol, CA4 and Psca), indicating the overall gene expression divergence between species was very low. Differentially expressed genes (DEGs) were then examined between parallel tissues (belly vs. belly, nape vs. nape, mantle vs. mantle, yellow rump vs. yellow rump, and tail vs. tail). We found 327 DEGs between bellies, 314 between napes, 708 between mantles, 827 between yellow rumps and 424 between tails, with a P-value cut-off for FDR<0.001 and a fold change of 2 (Figure S19). Combined, this analysis identified 1862 differentially expressed genes which were used in the comparable analysis between golden pheasant and Lady Amherst’s pheasant (Figure S20). Focusing on genes with colour-specific expression, 13 melanogenesis genes (ADCY9, ASIP, EDNRB, Gnaq, KIT, LEF1, PRKCA, TCF7L1, TCF7L2, TYR, TYRP1, WNT11, WNT16) and eight solute carrier family members (SLC15A1,SLC16A6, SLC1A4, SLC23A1, SLC24A4, SLC27A3, SLC45A2, SLC7A5) were expressed differentially. Within this list of genes, EDNRB, KIT, WNT16, SLC45A2 are highly expressed in yellow or red feathers, indicating their functions in pheomelanin synthesis, while TYRP1 and WNT11 are expressed highly in eumelanin contained feathers.

**Supplementary References**

1. Hill G and McGraw K. Bird coloration Vol. 1. Mechanisms and measurements. Massachusetts: Harvard University Press, Cambridge; 2006.

2. LaFountain AM, Kaligotla S, Cawley S, Riedl KM, Schwartz SJ, Frank HA, et al. Novel methoxy-carotenoids from the burgundy-colored plumage of the Pompadour Cotinga Xipholena punicea. Arch Biochem Biophys. 2010;504 1:142-53. doi:10.1016/j.abb.2010.08.006.

3. Thomas DB, McGraw KJ, Butler MW, Carrano MT, Madden O and James HF. Ancient origins and multiple appearances of carotenoid-pigmented feathers in birds. Proc Biol Sci. 2014;281 1788:20140806. doi:10.1098/rspb.2014.0806.

4. Thomas DB, McGraw KJ, James HF and Madden O. Non-destructive descriptions of carotenoids in feathers using Raman spectroscopy. Anal Methods-Uk. 2014;6 5:1301-8. doi:10.1039/c3ay41870g.

5. Thomas DB, McGoverin CM, McGraw KJ, James HF and Madden O. Vibrational spectroscopic analyses of unique yellow feather pigments (spheniscins) in penguins. J R Soc Interface. 2013;10 83:20121065. doi:10.1098/rsif.2012.1065.

6. Galvan I, Jorge A, Solano F and Wakamatsu K. Vibrational characterization of pheomelanin and trichochrome F by Raman spectroscopy. Spectrochim Acta A Mol Biomol Spectrosc. 2013;110:55-9. doi:10.1016/j.saa.2013.03.027.

7. Galvan I, Jorge A, Ito K, Tabuchi K, Solano F and Wakamatsu K. Raman spectroscopy as a non-invasive technique for the quantification of melanins in feathers and hairs. Pigment Cell Melanoma Res. 2013;26 6:917-23. doi:10.1111/pcmr.12140.

8. McGraw KJ, Hill GE, Stradi R and Parker RS. The effect of dietary carotenoid access on sexual dichromatism and plumage pigment composition in the American goldfinch. Comp Biochem Physiol B Biochem Mol Biol. 2002;131 2:261-9.

9. McGraw KJ, Hudon J, Hill GE and Parker RS. A simple and inexpensive chemical test for behavioral ecologists to determine the presence of carotenoid pigments in animal tissues. Behav Ecol Sociobiol. 2005;57 4:391-7. doi:10.1007/s00265-004-0853-y.

10. Wakamatsu K, Ohtara K and Ito S. Chemical analysis of late stages of pheomelanogenesis: conversion of dihydrobenzothiazine to a benzothiazole structure. Pigment Cell Melanoma Res. 2009;22 4:474-86. doi:10.1111/j.1755-148X.2009.00580.x.

11. Ito S, Nakanishi Y, Valenzuela RK, Brilliant MH, Kolbe L and Wakamatsu K. Usefulness of alkaline hydrogen peroxide oxidation to analyze eumelanin and pheomelanin in various tissue samples: application to chemical analysis of human hair melanins. Pigm Cell Melanoma R. 2011;24 4:605-13. doi:10.1111/j.1755-148X.2011.00864.x.

12. Toral GM, Figuerola J and Negro JJ. Multiple ways to become red: pigment identification in red feathers using spectrometry. Comp Biochem Physiol B Biochem Mol Biol. 2008;150 2:147-52. doi:10.1016/j.cbpb.2008.02.006.

13. Ito S, Wakamatsu K and Ozeki H. Chemical analysis of melanins and its application to the study of the regulation of melanogenesis. Pigment Cell Res. 2000;13 Suppl 8:103-9.

14. Poelstra JW, Vijay N, Hoeppner MP and Wolf JB. Transcriptomics of colour patterning and coloration shifts in crows. Mol Ecol. 2015;24 18:4617-28. doi:10.1111/mec.13353.

15. Sankoff D. Rearrangements and chromosomal evolution. Curr Opin Genet Dev. 2003;13 6:583-7.

16. Mortazavi A, Williams BA, McCue K, Schaeffer L and Wold B. Mapping and quantifying mammalian transcriptomes by RNA-Seq. Nat Methods. 2008;5 7:621-8. doi:10.1038/nmeth.1226.

17. Chen S, Yang P, Jiang F, Wei Y, Ma Z and Kang L. De novo analysis of transcriptome dynamics in the migratory locust during the development of phase traits. PLoS One. 2010;5 12:e15633. doi:10.1371/journal.pone.0015633.

18. Tarazona S, Garcia-Alcalde F, Dopazo J, Ferrer A and Conesa A. Differential expression in RNA-seq: a matter of depth. Genome Res. 2011;21 12:2213-23. doi:10.1101/gr.124321.111.

19. Wang L, Feng Z, Wang X, Wang X and Zhang X. DEGseq: an R package for identifying differentially expressed genes from RNA-seq data. Bioinformatics. 2010;26 1:136-8. doi:10.1093/bioinformatics/btp612.

20. Roulin A and Ducrest AL. Genetics of colouration in birds. Semin Cell Dev Biol. 2013;24 6-7:594-608. doi:10.1016/j.semcdb.2013.05.005.
