# Supplementary material for: Comparative genomics and transcriptomics of Chrysolophus provide insights into the evolution of complex plumage coloration
Source: Gigascience. 2018 Sep 6;7(10):giy113. doi: 10.1093/gigascience/giy113 (PMC6204425; doi:10.1093/gigascience/giy113)
Supplement: Response_to_Reviewer_Comments_Report_(Original_Submission).pdf [file giy113_response_to_reviewer_comments_report_(original_submission).pdf]

Author's Response To Reviewer Comments

Close

Dear editor,
Thank you so much for giving us the opportunity to submit a revised version of our manuscript (The manuscript ID is: GIGA-D-18-00007). Frankly, we wanted to apologize for the delay in submitting the manuscript back to you, as the analyses and rewriting have taken too much time. Now we have carefully considered your feedback and submit this revised manuscript, in which the points from both reviewers are fully addressed.
For your point of comparisons to other recently completed (Phasianidae) genomes, we failed to use the five birds you advised because of no released assembly (willow grouse, rock ptarmigan, and gunnison sage grouse), poor quality assembly (black grouse, scaffold N50 was only 1,029 bp and maximum scaffold was only 11,073 bp), or the URL does not exist (prairie chicken, https://dovetailgenomics.com/wp-content/uploads/2015/12/Dovetail-Case-Study-Prarie-Chicken). We have supplemented three sequenced Galliformes species instead (Japanese quail, northern bobwhite, and scaled quail) to our comparative analyses to update the genomic comparison.
For the comments from Reviewer #1, we have performed expanded studies by adding three other Galliformes genomes in analyses of phylogenetic relationship and specific varied genes in golden pheasant. The study on golden pheasant CYP2J19 gene were supplemented either. For the comments from Reviewer #2, β-keratin subfamilies were re-annotated and analyses of their expressions and proportions were carried out. Besides, journal style was conformed, and DOI link of GigaDB dataset were cited in the "data availability. In the end, we accepted the reviewer's suggestion to improve the style of written English. The language of our revised manuscript has also been edited by American Journal Experts (Order ID J9ZTP83V).
Responses to each of the Reviewers' comments are listed below.

Thank you for your reconsideration.

Sincerely yours,
Meng Xu (On behalf of all the authors)
BGI Genomics, BGI-Shenzhen, Shenzhen 518083, China
Email: xumeng@bgi.com

Reviewer reports:

Reviewer #1: This manuscript used genomics, transcriptomics, spectroscopy and chromatography to investigate the genetic basis for complex coloration in the plumage of Chrysolophus pheasants. Given their striking differences in coloration but close sister relationship, they seem to be an excellent model for understanding pathways leading to divergence of coloration in birds. I do not have any major concerns about this manuscript, but I do have a few suggestions for how to improve it:

General comment 1: Probably the most important issue is that this needs to undergo more thorough editing of its English. It is largely understandable as is, albeit with some confusing passages, but it would benefit from being checked by a fluent English-speaker.
Response: Thanks for your suggestion and sorry for our un-native and poor English. We have revised manuscript carefully. The language of our revised manuscript has also been

edited by American Journal Experts (Order ID J9ZTP83V).

General comment 2: I think this manuscript would benefit greatly from some expansion in the discussion regarding the evolution of coloration in birds. The manuscript currently reads as if it is simply reporting results without diving into how these results relate to the broader issue of the evolution of complex coloration in birds. Here we have an excellent example of closely related species with starkly contrasting coloration patterns, with some possible evidence of differences in gene expression and mutations, but not much is made of this, nor or is it related to the broader literature. Has anyone else looked at gene expression differences related to coloration in birds in a genome-wide scale? What about candidate gene approaches such as that of CYP2J19? Are there any overlaps with gene-specific studies? And more broadly, what might this mean about how the evolution of the same coloration can proceed via similar or divergent pathways? This might help draw more readers to your system and make an effort to dive into these questions in more detail. Of course, do not try to stretch your conclusions more than the data allow, and there is no need to review the entire literature, but I think there should certainly be more extensive exploration of what your data mean in the bigger picture.
Response: Thank you for your recognition of our work and giving us most valuable and helpful advices. Previous investigations on bird coloration in a genome-wide scale include crow and ruff genomes, which are based on Population Genomics (Poelstra JW et al. 2014, Science; Lamichhaney S et al. 2016, Nature Genetics; Küpper C et al. 2016, Nature Genetics). These studies identified fragment recombination in the avian genome whose melanin-based colorations are different. For the studies of coat-color related genes, most of the publications explored the polymorphism of single gene involved in melanogenesis, such as Mc1R (Page 3 line 16). Different expressions of ASIP and MITF were investigated mainly in mammals. It may be because there were less avian genomes published by the end of 2014. Therefore, reports on the mechanism of bird coloration at the genomic level are still limited. On the other hand, vertebrate carotenoid-based coloration is specific to avian, compared to mammals. And the utilization of this diet-derived pigment is hard to investigate at gene level. So it is lack of literature about genetic mechanisms on carotenoid-based coloration in avian species. Our current study is based on de novo sequencing of a single individual, which could provide a reference assembly for the next work of population genomes re-sequencing or the transcriptome sequencing in the future. Besides, we focused on both melanin-based and carotenoid-based colors in golden pheasant, because this species is one of the few Galliformes to express carotenoids in the feather follicles. We have added some literatures in the revised manuscript for the ASIP and MITF genes (Page 11 line 2; Page 11 line 17). This study identified a mechanism involving relatively few elements that can regulate the selective deposition of carotenoids, eumelanin/pheomelanin to produce the vast array of color patterns in avian plumage. This study also provides a platform for explaining how the complex patterns and shades of avian plumage colors are produced by differential and combinatorial use of this multi-layered coloring mechanism involving melanins, carotenoids and β-keratins. It could be proposed that extraordinarily complex plumage patterns are not only encoded by the genome but also produced by the mechanisms underlying multi-layered plumage coloring (Page 17 line 7; revised Additional file 1: Figure S16). We believe these data are conducive to more extensive and systemically explorations on golden pheasant or even on multiple avian species at the genome level in the future. Analyses of CYP2J19 and specific genes in golden pheasant are supplemented in this revised manuscript as you advised. Details of our responses are described below.
Page 7, lines 6-8: while I appreciate the effort to reconstruct the phylogeny of Chrysolophus relative to Meleagris and Gallus, this seems to be uncontroversial with other studies having

supported the Chrysolophus+Meleagris relationship with far better taxon sampling (e.g., Wang et al. 2013 PLoS One). Even though the authors here use many more loci to resolve this relationship, it seems to simply recapitulate a result that was already pretty clear, and it is ultimately not relevant to the focus of this study.
Response: Thanks for your suggestion. We have accepted your advice by adding more species to the comparative genome analyses. According to the species mentioned in the publication (Wang et al. 2013 PLoS One) and genome available avian, we have reconstruct the phylogeny using six Galliformes (golden pheasant, chicken, turkey, Japanese quail, northern bobwhite and scaled quail), the sequenced Anseriformes (duck) which is closest to Galliformes, and a model species (zebra finch) as an outgroup (Page 7 line 4; Figure 2b).

Page 8, lines 15-17: note that the two studies on CYP2J19 only looked at passerines, whereas Emerling 2018 (Molecular Phylogenetics and Evolution) provided evidence that this gene is responsible for the production of red carotenoids more broadly across Aves. I think this is interesting and significant for the broader question of coloration in birds (see General Comment 2 above). For example, why did the Chrysolophus species not co-opt CYP2J19 to create their striking red coloration? This of course is not testable in the current study, and perhaps merely speculative, but I think this is relevant to the broader question of how and why birds convergently evolved similar coloration but often via different mechanisms.
Response: We accepted the reviewer's suggestion. CYP2J19 were comparatively analyzed within the eight avian species mentioned above. We download the protein sequences of CYP2J19(A/B) and CYP2J40 provided by Mundy (Mundy NI et al. 2016, Current Biology, doi:10.1016/j.cub.2016.04.047), and their un- and downstream genes (HOOK1 and NFIA), aligned to the compared genomes using the same pipeline. Through combining the syntenic relationship and alignment identity, we identified the CYP2J19 in the genomes. For copy number, there is only one copy of CYP2J19 in Galliformes and duck genome but two copies in zebra finch. For multiple sequence alignment, CYP2J19 protein sequences are similar in Galliformes. The golden pheasant sequence is more similar to the turkey. The expression of CYP2J19 in the orange nape of golden pheasant was significantly higher than other colored feathers in Chrysolophus. It suggested that CYP2J19 might play roles at transcriptional level in Chrysolophus. These results were supplemented in the last paragraph of "Evolution analysis within Galliformes" of the revised manuscript (Page 8 line 17).
On the other hand, we found the red color of golden pheasant were not red-carotenoid based coloration based on our pigment identification experiments. The extracted lipid pigments from red feathers exhibited visual yellow. The red golden pheasant belly feathers remained the initial color even after the thermochemical procedures, and the aqueous phase was still colored even after treatment with multiple extractions (Figure S17). The aqueous phase of nape orange feathers was colorless after extractions, and the once-pigmented tissues nearly lost their color entirely. It can be indicated that different carotenoids are present in different feathers in golden pheasant (Details are described in Additional file 3: Supplementary Notes 1.2). The different expression of CYP2J19 in golden pheasant feathers may affect the utilization of carotenoids. But the influence of sequence evolution remains unclear for golden pheasant. Many birds have CYP2J19 gene, but can't accumulate carotenoid in feather, such as the other Galliformes in this study, and zebra finch although its beak can accumulate carotenoid. The independent evolution for feather carotenoid in several birds indicate this ability may be controlled or impacted by multiple genes.

Page 9, line 12: you discuss lineage specific "varied" genes in Chrysolophus. First of all, it is not clear what is meant by varied genes and the significance of this, but I assume this is

getting at genetic synapomorphies (substitutions) or something of that nature. First of all, I'm unsure if such a metric is relevant because it is not clear if you're referring to all substitutions/variations, or simply those that are nonsynynomous, which are usually what researchers discuss. Regardless, you refer to them as being lineage specific and discuss it in the context of Chrysolophus. It is important, however, to note that any substitutions present in Chrysolophus may also be shared by at least another six genera (Wang et al. 2013). So any significance gleaned from these analyses should be tempered by the understanding that they might not be specific to Chrysolophus only.
Response: We accepted the reviewer's suggestion. We have re-written some sentences/words. We identified the lineage-specific varied genes based on the protein sequences, which mean the variations were nonsynynomous. According to the the publication (Wang et al. 2013 PLoS One), most mentioned Phasianidae species are not genome available. Therefore, we have added three sequenced Galliformes (Japanese quail, northern bobwhite, and scaled quail) to our genomic comparative analyses. In this way, the lineage-specific varied gene set could be gleaned to a certain extent (Page 9 line 21). Actually, except the Galliformes, we also used 11 other birds as background. These background birds should be enough to identify the conserved genotype among the bird evolution.

Reviewer #2: The manuscript entitled "Comparative genomics and transcriptomics of Chrysolophus provide insights into the evolution of complex plumage coloration" sequenced two pheasant genomes and performed comparative genomics and transcriptomics to identify variations in plumage related genes. Overall, this is a significant and thorough study and should be published. However, the grammar and word choice is poor and the writing style is awkward and needs significant work. In this review, I did not spend time trying to correct these issues. I do, however, point out instances where the poor writing effects the understanding of the results.
Response: Thank you for your recognition of our work and giving us most valuable and helpful advices. We accepted the reviewer's suggestion to improve the style of written English. The language of our revised manuscript has also been edited by American Journal Experts (Order ID J9ZTP83V).

Results and Discussion
Page 8, lines 3-4 "For the multi-copy families……" sentence. Do the authors mean gene families? Are the 31 lineage specific gene families simply being identified by number or are members of the gene families showing evidence of positive selection? Please clarify the meaning of this sentence.
Response: Yes, it means multi-copy gene families which were identified in golden pheasant by hierarchical clustering. This sentence has been clarified (Page 8 line 2). The gene family was regarded as lineage specific if its containing genes were only from one species. The positive selective analysis was based on the one-to-one orthologous genes.

Page 14, While I think it is interesting that they analyzed the relationship of β-keratin copy number and sequencing depth, I think they should also focus on identifying the β-keratin subfamily (feather, claw, keratinocyte, etc.) membership of the genes that were found to be differentially expressed. Also, despite the underrepresentation of β-keratins in sequenced genomes, they should still be able to identify the relative proportions of the β-keratin subfamilies in the pheasant genomes compared to other avian species.
Response: Thanks for your valuable suggestion. The β-keratins were re-annotated to subfamily based on the best hit to the β-keratins of zebra finch according to previous studies

(Greenwold MJ and Sawyer RH, J Exp Zool B Mol Dev Evol, 2013). Then we updated the analyses of β-keratins and performed a phylogenetic analysis of β-keratins in six Galliformes (Page 14 line 23, Page 15 line 4).

Methods

The authors need to specify the identity of the tissue used for the comparative transcriptome work. I was only able to find references to the color of the feathers. Were these adult or juvenile feathers? Taken during a molt etc.? Which part of the feather was tissue taken?

Response: Acknowledged and accepted your suggestion. For the feather follicles sampling, we used the previously published method (Poelstra JW et al. 2014, Science), briefly, we plucked their feathers during natural molting, then allowed feathers to grow for two-four weeks, at which stage the previously plucked areas of skin contained densely spaced feather shafts with the first parts of feathers about to or just protruding from the shafts. These methods are present in Additional file 3: Supplementary Notes 2.2.

Figure 2 legends.

(c) is the Venn diagram of gene families? The legend and main text are confusing.

(d) What are the "clades" of these species and why were those specific species chosen in those clades? Better genome builds? Please specify.

Response: Thank you for your suggestion.

(c) Sorry for the confused writing and we have uniformed the writing (Page 8 line 2, Page 34 line 1).

(d) Yes, the selected species in each clade because of their better assembly, we have supplemented the description in the Method part (Page 9 line 21, Page 34 line 4).

Close
