# Supplementary material for: Comparative genomics and transcriptomics of Chrysolophus provide insights into the evolution of complex plumage coloration
Source: Gigascience. 2018 Sep 6;7(10):giy113. doi: 10.1093/gigascience/giy113 (PMC6204425; doi:10.1093/gigascience/giy113)
Supplement: Response_to_Reviewer_Comments_Report_Revision_1.pdf [file giy113_response_to_reviewer_comments_report_revision_1.pdf]

Author's Response To Reviewer Comments

Close

Dear editor,
Thank you so much for your recognition of our work and giving us the opportunity to submit a further revised version of our revised manuscript (GIGA-D-18-00007R1). In this revised manuscript, we have accepted all of the suggestions from reviewers 2 and removed colorful highlighted sentences. The formats of references are set up according to "GigaScience.ens" by EndNote software. We have uploaded the raw sequencing data to NCBI. The accession numbers of the sequencing data have been mentioned in the section of "DATA AVAILABILITY" in this present revised manuscript.
Thank you for the work you have done for us.
Best wishes,
Sincerely yours,
Meng Xu (On behalf of all the authors)
BGI Genomics, BGI-Shenzhen, Shenzhen 518083, China
Email: xumeng@bgi.com

Reviewer reports:
Reviewer #1: I believe the authors satisfactorily addressed my comments and the manuscript should now be acceptable for publication.
Response: Thank you for your recognition of our work. We wish you all the best in the future.

Reviewer #2: The manuscript entitled "Comparative genomics and transcriptomics of Chrysolophus provide insights into the evolution of complex plumage coloration" is much improved and I only have a few minor suggested edits before publication.
Response: Thank you for your recognition of our work and giving us most professional and precise advices in the concepts descripting and language editing. We have accepted all of your suggestions in this present revised manuscript. Responses to each of the comments are listed below.

Background
Page 3, lines 24-25. In this last sentence, "In birds, keratins….." the inclusion of alpha- and beta-keratins in parentheses following keratins should be included here. Alpha- and beta-keratins, while they have similar names (and generally function), do not share an evolutionary history and are therefore distinct gene families.
Response: Acknowledged and complied. The initial sentence "In birds, keratins belong to a large family with nearly 200 members" has been changed into "In birds, α- and β-keratins families include nearly 200 members".

Results and Discussion
Page 7, line 8. Change avian to birds in the "analysis of 48 avian concluded"
Response: Acknowledged and complied.

Page 7, line 12. Change "that do not affect the amino acids coding…" to "which are sites that do not change the amino acid…."
Response: Acknowledged and complied.

Page 8, lines 7. Change "which was just one copy in all the 48 birds [16]. Although this gene is multiple copies in northern bobwhite or scaled quail, it should be independently events for the duplication in the two Odontophoridae species and golden pheasant respectively based on the phylogeny (Figure 2d)."
to
"whereas only one copy was found in 48 other birds [16]. Although multiple copies of this gene is present in northern bobwhite or scaled quail, it's likely that independent duplication events occurred in the two Odontophoridae species and golden pheasant respectively based on our phylogeny (Figure 2d)."
Response: Acknowledged and complied.

Page 8, line 19. Change "golden pheasant unchanged" to "golden pheasant is conserved".
Response: Acknowledged and complied.

Page 8, lines 21-22. Change " might play roles at" to "might influence coloration at"
Response: Acknowledged and complied.

Page 9, line 1. Change "pheasant may benefit the metabolism" to "pheasant may function in metabolism"
Response: Acknowledged and complied.

Page 9, line 10. Remove the words "evolution factor" from sentence.
Response: Acknowledged and complied.

Page 12, lines 10-11. Change "have been revealed the presence of carotenoids" to "have been found to possess carotenoids"
Response: Acknowledged and complied.

Conclusion
Page 17, line 16. Should "sufficient thus far" be changed to "feasible at this time"?
Response: Acknowledged and complied.

Page 17, lines 17-20. I rewrote this sentence below. I think this retains the meaning of the authors. Regardless, I think this last sentence needs revision. Please review.

"Species models of coloration can provide insight into the evolution and regulation of plumage coloration. Here we present the golden pheasant and its sister pheasant genomes to serve as candidate models for future studies on plumage coloration."
Response: Acknowledged and complied. Thank you for your patience and carefulness in helping us to revise the sentences.

Methods
Page 18-19. Last sentence starting on page 18 needs revision. It's not clear what "six kinds of phase" are here.
Response: Sorry for our inadequate description. The sentence of "the longest ORF from six kinds of phase was selected" has been changed into "we tried all the possible translations for RNA to protein (three phases for plus and minus strand respectively) and selected the longest ORF for each transcript".

Figures
Figure 2d: Jarvis' phylogeny…..needs a citation. This should be citation [16].
Response: Acknowledged and citation added.

Close
