# Supplementary material for: Comparative genomics and transcriptomics of Chrysolophus provide insights into the evolution of complex plumage coloration
Source: Gigascience. 2018 Sep 6;7(10):giy113. doi: 10.1093/gigascience/giy113 (PMC6204425; doi:10.1093/gigascience/giy113)
Supplement: Reviewer_1_Report_(Original_Submission) -- Christopher Emerling — 2/12/2018 Reviewed [file giy113_reviewer_1_report_(original_submission).pdf]

Reviewer Report

Title: **Comparative genomics and transcriptomics of Chrysolophus provide insights into the evolution of complex plumage coloration**

Version: **Original Submission** **Date:** 2/12/2018

Reviewer name: **Christopher Emerling**

Reviewer Comments to Author:

This manuscript used genomics, transcriptomics, spectroscopy and chromatography to investigate the genetic basis for complex coloration in the plumage of Chrysolophus pheasants. Given their striking differences in coloration but close sister relationship, they seem to be an excellent model for understanding pathways leading to divergence of coloration in birds.

I do not have any major concerns about this manuscript, but I do have a few suggestions for how to improve it:

General comment 1: Probably the most important issue is that this needs to undergo more thorough editing of its English. It is largely understandable as is, albeit with some confusing passages, but it would benefit from being checked by a fluent English-speaker.

General comment 2: I think this manuscript would benefit greatly from some expansion in the discussion regarding the evolution of coloration in birds. The manuscript currently reads as if it is simply reporting results without diving into how these results relate to the broader issue of the evolution of complex coloration in birds. Here we have an excellent example of closely related species with starkly contrasting coloration patterns, with some possible evidence of differences in gene expression and mutations, but not much is made of this, nor or is it related to the broader literature. Has anyone else looked at gene expression differences related to coloration in birds in a genome-wide scale? What about candidate gene approaches such as that of CYP2J19? Are there any overlaps with gene-specific studies? And more broadly, what might this mean about how the evolution of the same coloration can proceed via similar or divergent pathways? This might help draw more readers to your system and make an effort to dive into these questions in more detail. Of course, do not try to stretch your conclusions more than the data allow, and there is no need to review the entire literature, but I think there should certainly be more extensive exploration of what your data mean in the bigger picture.

Page 7, lines 6-8: while I appreciate the effort to reconstruct the phylogeny of Chrysolophus relative to Meleagris and Gallus, this seems to be uncontroversial with other studies having supported the Chrysolophus+Meleagris relationship with far better taxon sampling (e.g., Wang et al. 2013 PLoS One). Even though the authors here use many more loci to resolve this relationship, it seems to simply recapitulate a result that was already pretty clear, and it is ultimately not relevant to the focus of this study.

Page 8, lines 15-17: note that the two studies on CYP2J19 only looked at passerines, whereas Emerling 2018 (Molecular Phylogenetics and Evolution) provided evidence that this gene is responsible for the production of red carotenoids more broadly across Aves. I think this is interesting and significant for the broader question of coloration in birds (see General Comment 2 above). For example, why did the Chrysolophus species not co-opt CYP2J19 to create their striking red coloration? This of course is not testable in the current study, and perhaps merely speculative, but I think this is relevant to the broader question of how and why birds convergently evolved similar coloration but often via different mechanisms.

Page 9, line 12: you discuss lineage specific "varied" genes in Chrysolophus. First of all, it is not clear what is meant by varied genes and the significance of this, but I assume this is getting at genetic synapomorphies

(substitutions) or something of that nature. First of all, I'm unsure if such a metric is relevant because it is not clear if you're referring to all substitutions/variations, or simply those that are nonsynynomous, which are usually what researchers discuss. Regardless, you refer to them as being lineage specific and discuss it in the context of Chrysolophus. It is important, however, to note that any substitutions present in Chrysolophus may also be shared by at least another six genera (Wang et al. 2013). So any significance gleaned from these analyses should be tempered by the understanding that they might not be specific to Chrysolophus only.

Methods

Are the methods appropriate to the aims of the study, are they well described, and are necessary controls included? Yes

Conclusions

Are the conclusions adequately supported by the data shown? Yes

Reporting Standards

Does the manuscript adhere to the journal's guidelines on minimum standards of reporting? Yes

Choose an item.

Statistics

Are you able to assess all statistics in the manuscript, including the appropriateness of statistical tests used? Yes, and I have assessed the statistics in my report.

Quality of Written English

Please indicate the quality of language in the manuscript: Needs some language corrections before being published

Declaration of Competing Interests

Please complete a declaration of competing interests, considering the following questions:

- Have you in the past five years received reimbursements, fees, funding, or salary from an organisation that may in any way gain or lose financially from the publication of this manuscript, either now or in the future?
- Do you hold any stocks or shares in an organisation that may in any way gain or lose financially from the publication of this manuscript, either now or in the future?
- Do you hold or are you currently applying for any patents relating to the content of the manuscript?

- Have you received reimbursements, fees, funding, or salary from an organization that holds or has applied for patents relating to the content of the manuscript?
- Do you have any other financial competing interests?
- Do you have any non-financial competing interests in relation to this paper?

If you can answer no to all of the above, write 'I declare that I have no competing interests' below. If your reply is yes to any, please give details below.

I declare that I have no competing interests

I agree to the open peer review policy of the journal. I understand that my name will be included on my report to the authors and, if the manuscript is accepted for publication, my named report including any attachments I upload will be posted on the website along with the authors' responses. I agree for my report to be made available under an Open Access Creative Commons CC-BY license (http://creativecommons.org/licenses/by/4.0/). I understand that any comments which I do not wish to be included in my named report can be included as confidential comments to the editors, which will not be published.

I agree to the open peer review policy of the journal

To further support our reviewers, we have joined with Publons, where you can gain additional credit to further highlight your hard work (see: https://publons.com/journal/530/gigascience). On publication of this paper, your review will be automatically added to Publons, you can then choose whether or not to claim your Publons credit. I understand this statement.

Yes Choose an item.
