# Supplementary material for: Comparative genomics and transcriptomics of Chrysolophus provide insights into the evolution of complex plumage coloration
Source: Gigascience. 2018 Sep 6;7(10):giy113. doi: 10.1093/gigascience/giy113 (PMC6204425; doi:10.1093/gigascience/giy113)
Supplement: Reviewer_2_Report_(Original_Submission) -- Matthew Greenwold — 4/1/2018 Reviewed [file giy113_reviewer_2_report_(original_submission).pdf]

Reviewer Report

Title: Comparative genomics and transcriptomics of Chrysolophus provide insights into the evolution of complex plumage coloration

Version: Original Submission **Date:** 4/1/2018

Reviewer name: Matthew Greenwold

Reviewer Comments to Author:

The manuscript entitled "Comparative genomics and transcriptomics of Chrysolophus provide insights into the evolution of complex plumage coloration" sequenced two pheasant genomes and performed comparative genomics and transcriptomics to identify variations in plumage related genes. Overall, this is a significant and thorough study and should be published. However, the grammar and word choice is poor and the writing style is awkward and needs significant work. In this review, I did not spend time trying to correct these issues. I do, however, point out instances where the poor writing effects the understanding of the results.

Results and Discussion

Page 8, lines 3-4 "For the multi-copy families……" sentence. Do the authors mean gene familes? Are the 31 lineage specific gene families simply being identified by number or are members of the gene families showing evidence of positive selection? Please clarify the meaning of this sentence.

Page 14, While I think it is interesting that they analyzed the relationship of β-keratin copy number and sequencing depth, I think they should also focus on identifying the β-keratin subfamily (feather, claw, keratinocyte, etc.) membership of the genes that were found to be differentially expressed. Also, despite the underrepresentation of β-keratins in sequenced genomes, they should still be able to identify the relative proportions of the β-keratin subfamilies in the pheasant genomes compared to other avian species.

Methods

The authors need to specify the identity of the tissue used for the comparative transcriptome work. I was only able to find references to the color of the feathers. Were these adult or juvenile feathers? Taken during a molt etc.? Which part of the feather was tissue taken?

Figure 2 legends.

(c) is the Venn diagram of gene families? The legend and main text are confusing.

(d) What are the "clades" of these species and why were those specific species chosen in those clades? Better genome builds? Please specify.

Methods

Are the methods appropriate to the aims of the study, are they well described, and are necessary controls included? Yes

Conclusions

Are the conclusions adequately supported by the data shown? Yes

Reporting Standards

Does the manuscript adhere to the journal's guidelines on [minimum standards of reporting?] Yes

Choose an item.

Statistics

Are you able to assess all statistics in the manuscript, including the appropriateness of statistical tests used? Yes, and I have assessed the statistics in my report.

Quality of Written English

Please indicate the quality of language in the manuscript: Not suitable for publication unless extensively edited

Declaration of Competing Interests

Please complete a declaration of competing interests, considering the following questions:

- Have you in the past five years received reimbursements, fees, funding, or salary from an organisation that may in any way gain or lose financially from the publication of this manuscript, either now or in the future?
- Do you hold any stocks or shares in an organisation that may in any way gain or lose financially from the publication of this manuscript, either now or in the future?
- Do you hold or are you currently applying for any patents relating to the content of the manuscript?
- Have you received reimbursements, fees, funding, or salary from an organization that holds or has applied for patents relating to the content of the manuscript?
- Do you have any other financial competing interests?
- Do you have any non-financial competing interests in relation to this paper?

If you can answer no to all of the above, write 'I declare that I have no competing interests' below. If your reply is yes to any, please give details below.

I declare that I have no competing interests

I agree to the open peer review policy of the journal. I understand that my name will be included on my report to the authors and, if the manuscript is accepted for publication, my named report including any attachments I upload will be posted on the website along with the authors' responses. I agree for my report to be made available under an Open Access Creative Commons CC-BY license (http://creativecommons.org/licenses/by/4.0/). I understand that any comments which I do not wish to be included in my named report can be included as confidential comments to the editors, which will not be published.

I agree to the open peer review policy of the journal

To further support our reviewers, we have joined with Publons, where you can gain additional credit to further highlight your hard work (see: https://publons.com/journal/530/gigascience). On publication of this paper, your review will be automatically added to Publons, you can then choose whether or not to claim your Publons credit. I understand this statement.

Yes Choose an item.
