# Supplementary material for: Comparative genomics and transcriptomics of Chrysolophus provide insights into the evolution of complex plumage coloration
Source: Gigascience. 2018 Sep 6;7(10):giy113. doi: 10.1093/gigascience/giy113 (PMC6204425; doi:10.1093/gigascience/giy113)
Supplement: Reviewer_2_Report_Revision_1 -- Matthew Greenwold — 7/18/2018 Reviewed [file giy113_reviewer_2_report_revision_1.pdf]

Reviewer Report

Title: **Comparative genomics and transcriptomics of Chrysolophus provide insights into the evolution of complex plumage coloration**

Version: **Original Submission** **Date:** 7/18/2018

Reviewer name: **Matthew Greenwold**

Reviewer Comments to Author:

The manuscript entitled "Comparative genomics and transcriptomics of Chrysolophus provide insights into the evolution of complex plumage coloration" is much improved and I only have a few minor suggested edits before publication.

Background

Page 3, lines 24-25. In this last sentence, "In birds, keratins….." the inclusion of alpha- and beta-keratins in parentheses following keratins should be included here. Alpha- and beta-keratins, while they have similar names (and generally function), do not share an evolutionary history and are therefore distinct gene families.

Results and Discussion

Page 7, line 8. Change avian to birds in the "analysis of 48 avian concluded"

Page 7, line 12. Change "that do not affect the amino acids coding…" to "which are sites that do not change the amino acid…."

Page 8, lines 7. Change "which was just one copy in all the 48 birds [16]. Although this
gene is multiple copies in northern bobwhite or scaled quail, it should be independently events for the duplication in the two Odontophoridae species and golden pheasant respectively based on the phylogeny (Figure 2d)."
to
"whereas only one copy was found in 48 other birds [16]. Although multiple copies of this gene is present in northern bobwhite or scaled quail, it's likely that independent duplication events occurred in the two Odontophoridae species and golden pheasant respectively based on our phylogeny (Figure 2d)."

Page 8, line 19. Change "golden pheasant unchanged" to "golden pheasant is conserved".

Page 8, lines 21-22. Change " might play roles at" to "might influence coloration at"

Page 9, line 1. Change "pheasant may benefit the metabolism" to "pheasant may function in metabolism"

Page 9, line 10. Remove the words "evolution factor" from sentence.

Page 12, lines 10-11. Change "have been revealed the presence of carotenoids" to "have been found to possess carotenoids"

Conclusion

Page 17, line 16. Should "sufficient thus far" be changed to "feasible at this time"?

Page 17, lines 17-20. I rewrote this sentence below. I think this retains the meaning of the authors. Regardless, I think this last sentence needs revision. Please review.

"Species models of coloration can provide insight into the evolution and regulation of plumage coloration. Here we present the golden pheasant and its sister pheasant genomes to serve as candidate models for future studies on plumage coloration."

Methods

Page 18-19. Last sentence starting on page 18 needs revision. It's not clear what "six kinds of phase" are here.

Figures

Figure 2d: Jarvis' phylogeny…..needs a citation. This should be citation [16].

Methods

Are the methods appropriate to the aims of the study, are they well described, and are necessary controls included? Yes

Conclusions

Are the conclusions adequately supported by the data shown? Choose an item.

Reporting Standards

Does the manuscript adhere to the journal's guidelines on minimum standards of reporting? Choose an item.

Choose an item.

Statistics

Are you able to assess all statistics in the manuscript, including the appropriateness of statistical tests used? Choose an item.

Quality of Written English

Please indicate the quality of language in the manuscript: Choose an item.

Declaration of Competing Interests

Please complete a declaration of competing interests, considering the following questions:

- Have you in the past five years received reimbursements, fees, funding, or salary from an organisation that may in any way gain or lose financially from the publication of this manuscript, either now or in the future?

- Do you hold any stocks or shares in an organisation that may in any way gain or lose financially from the publication of this manuscript, either now or in the future?
- Do you hold or are you currently applying for any patents relating to the content of the manuscript?
- Have you received reimbursements, fees, funding, or salary from an organization that holds or has applied for patents relating to the content of the manuscript?
- Do you have any other financial competing interests?
- Do you have any non-financial competing interests in relation to this paper?

If you can answer no to all of the above, write 'I declare that I have no competing interests' below. If your reply is yes to any, please give details below.

I declare that I have no competing interests

I agree to the open peer review policy of the journal. I understand that my name will be included on my report to the authors and, if the manuscript is accepted for publication, my named report including any attachments I upload will be posted on the website along with the authors' responses. I agree for my report to be made available under an Open Access Creative Commons CC-BY license (http://creativecommons.org/licenses/by/4.0/). I understand that any comments which I do not wish to be included in my named report can be included as confidential comments to the editors, which will not be published.

I agree to the open peer review policy of the journal

To further support our reviewers, we have joined with Publons, where you can gain additional credit to further highlight your hard work (see: https://publons.com/journal/530/gigascience). On publication of this paper, your review will be automatically added to Publons, you can then choose whether or not to claim your Publons credit. I understand this statement.

Yes Choose an item.
